# Broad betacoronavirus neutralization by a stem helix–specific human antibody

**DOI:** 10.1126/science.abj3321

**Published:** 2021-09-03

**Authors:** Dora Pinto, Maximilian M. Sauer, Nadine Czudnochowski, Jun Siong Low, M. Alejandra Tortorici, Michael P. Housley, Julia Noack, Alexandra C. Walls, John E. Bowen, Barbara Guarino, Laura E. Rosen, Julia di Iulio, Josipa Jerak, Hannah Kaiser, Saiful Islam, Stefano Jaconi, Nicole Sprugasci, Katja Culap, Rana Abdelnabi, Caroline Foo, Lotte Coelmont, Istvan Bartha, Siro Bianchi, Chiara Silacci-Fregni, Jessica Bassi, Roberta Marzi, Eneida Vetti, Antonino Cassotta, Alessandro Ceschi, Paolo Ferrari, Pietro E. Cippà, Olivier Giannini, Samuele Ceruti, Christian Garzoni, Agostino Riva, Fabio Benigni, Elisabetta Cameroni, Luca Piccoli, Matteo S. Pizzuto, Megan Smithey, David Hong, Amalio Telenti, Florian A. Lempp, Johan Neyts, Colin Havenar-Daughton, Antonio Lanzavecchia, Federica Sallusto, Gyorgy Snell, Herbert W. Virgin, Martina Beltramello, Davide Corti, David Veesler

**Affiliations:** 1 Humabs Biomed SA, a subsidiary of Vir Biotechnology, 6500 Bellinzona, Switzerland.; 2 Department of Biochemistry, University of Washington, Seattle, WA 98195, USA.; 3 Vir Biotechnology, San Francisco, CA 94158, USA.; 4 Institute for Research in Biomedicine, Università della Svizzera italiana, 6500 Bellinzona, Switzerland.; 5 Rega Institute for Medical Research, Laboratory of Virology and Chemotherapy, KU Leuven, 3000 Leuven, Belgium.; 6 Clinical Trial Unit, Ente Ospedaliero Cantonale, 6900 Lugano, Switzerland.; 7 Division of Clinical Pharmacology and Toxicology, Institute of Pharmacological Sciences of Southern Switzerland, Ente Ospedaliero Cantonale, 6900 Lugano, Switzerland.; 8 Department of Clinical Pharmacology and Toxicology, University Hospital Zurich, 8091 Zurich, Switzerland.; 9 Faculty of Biomedical Sciences, Università della Svizzera italiana, 6900 Lugano, Switzerland.; 10 Department of Medicine, Ente Ospedaliero Cantonale, 6500 Bellinzona, Switzerland.; 11 Clinical School, University of New South Wales, Sydney, NSW, 2052, Australia.; 12 Faculty of Medicine, University of Zurich, 8057 Zurich, Switzerland.; 13 Intensive Care Unit, Clinica Luganese Moncucco, 6900 Lugano, Switzerland.; 14 Clinic of Internal Medicine and Infectious Diseases, Clinica Luganese Moncucco, 6900 Lugano, Switzerland.; 15 III Division of Infectious Diseases, ASST Fatebenefratelli Sacco, Luigi Sacco Hospital, 20157 Milan, Italy.; 16 Institute of Microbiology, ETH Zurich, 8093 Zurich, Switzerland.; 17 UT Southwestern Medical Center, Dallas, TX 75390, USA.; 18 Washington University School of Medicine, St. Louis, MO 63110, USA.

## Abstract

The spillovers of betacoronaviruses in humans and the emergence of severe acute respiratory syndrome coronavirus 2 (SARS-CoV-2) variants highlight the need for broad coronavirus countermeasures. We describe five monoclonal antibodies (mAbs) cross-reacting with the stem helix of multiple betacoronavirus spike glycoproteins isolated from COVID-19 convalescent individuals. Using structural and functional studies, we show that the mAb with the greatest breadth (S2P6) neutralizes pseudotyped viruses from three different subgenera through the inhibition of membrane fusion, and we delineate the molecular basis for its cross-reactivity. S2P6 reduces viral burden in hamsters challenged with SARS-CoV-2 through viral neutralization and Fc-mediated effector functions. Stem helix antibodies are rare, oftentimes of narrow specificity, and can acquire neutralization breadth through somatic mutations. These data provide a framework for structure-guided design of pan-betacoronavirus vaccines eliciting broad protection.

## Targeting a range of betacoranaviruses

In the past 20 years, three highly pathogenic β-coronaviruses have crossed from animals to humans, including the most recent: severe acute respiratory syndrome coronavirus 2 (SARS-CoV-2). A spike protein that decorates these viruses has an S1 domain that binds host cell receptors and an S2 domain that fuses the viral and cell membranes to allow cell entry. The S1 domain is the target of many neutralizing antibodies but is more genetically variable than S2, and antibodies can exert selective pressure, leading to resistant variants. Pinto *et al*. identified five monoclonal antibodies that interact with a helix in the S2 domain. The most broadly neutralizing antibody inhibited all β-coronavirus subgenera and reduced viral burden in hamsters infected with SARS-CoV-2. —VV

## Teaser

A broadly neutralizing monoclonal antibody targeting the conserved viral spike stem helix reduces SARS-CoV-2 burden in hamsters.

Coronaviruses are zoonotic pathogens responsible for several epidemics and a pandemic in the past two decades. All three highly pathogenic coronaviruses belong to the betacoronavirus genus: Severe acute respiratory syndrome coronavirus 2 (SARS-CoV-2) and SARS-CoV cluster within the *sarbecovirus* subgenus and originated in bats, whereas Middle East respiratory syndrome coronavirus (MERS-CoV) belongs to the *merbecovirus* subgenus and is transmitted to humans through dromedary camels. Moreover, HCoV-HKU1 and HCoV-OC43 (*embecovirus* subgenus, betacoronaviruses) as well as NL63 and 229E (*setracovirus* and *duvinacovirus* subgenera, alphacoronaviruses) are endemic and cause common colds in humans.

The coronavirus spike (S) glycoprotein promotes viral entry into host cells through an S_1_ subunit that engages host receptors and an S_2_ subunit mediating membrane fusion (*1*). The S_1_ subunit is the major target of (neutralizing) antibodies (Abs) and is more genetically variable than the S_2_ subunit (*2*–*5*). Accordingly, Abs binding to the S_1_ subunit receptor-binding domain (RBD) and N-terminal domain (NTD) exert a selective pressure that results in the emergence of new variants (*5*–*10*). SARS-CoV-2 S comprises 22 N-linked glycans (13 and 9 of them in the S_1_ and S_2_ subunits, respectively) participating in folding as well as modulating access to host proteases and antibodies (*1*, *2*, *11*–*13*). Monoclonal antibodies (mAbs) binding to the S_2_ fusion machinery can neutralize distantly related coronaviruses (*14*–*16*), as observed for other viruses such as influenza and HIV-1 (*17*, *18*).

## Results

### Isolation of a broadly neutralizing betacoronavirus mAb from a convalescent SARS-CoV-2–exposed individual

**Fig. 1.**
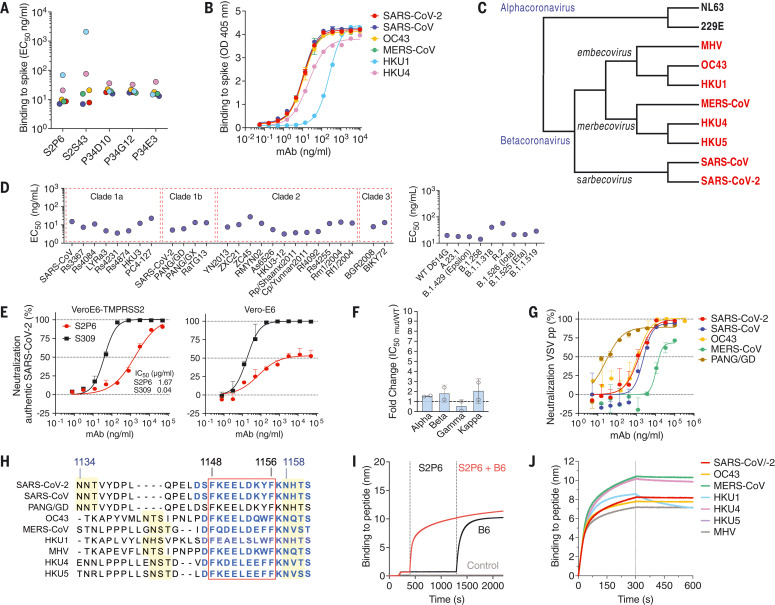
The S2P6 cross-reactive mAb broadly neutralizes betacoronaviruses from three subgenera. (**A**) Binding avidity (EC_50_) of five mAbs to prefusion coronavirus S trimer ectodomains as determined by ELISA. (**B**) S2P6 ELISA binding curves showing the full titration. One representative experiment out of two is shown. OD, optical density. (**C**) Cladogram of representative alpha- (black) and betacoronavirus (red) S glycoprotein amino acid sequences inferred through maximum likelihood analysis. (**D**) Flow cytometry analysis of S2P6 binding (from 10 to 0.22 μg/ml) to a panel of 26 S glycoproteins representative of all *sarbecovirus* clades (left) and eight SARS-CoV-2 variants (right) displayed as EC_50_ values. (**E**) Neutralization of authentic SARS-CoV-2 by S2P6 determined using VeroE6-TMPRSS2 (left) or Vero-E6 (right) cells. The S309 mAb that binds RBD site IV (*38*) is included for comparison. The mean ± SD of triplicates from one representative experiment out of three is shown. (**F**) S2P6-mediated neutralization of SARS-CoV-2 B.1.1.7 (Alpha) S, B.1.351 (Beta) S, P.1 (Gamma) S, and B1.617.1 (Kappa) S VSV pseudotypes (mut) represented as IC_50_ fold change relative to wild-type (WT) (D614G) S VSV pseudotype. Individual values for each of the two replicates are shown as open circles, the mean as a colored bar, and the SD as error bars. (**G**) S2P6-mediated neutralization of VSV pseudotyped with various betacoronavirus S glycoproteins. Error bars indicate standard deviation of triplicates. IC_50_ values are 2.4 μg/ml, 1.4 μg/ml, 17.1 μg/ml, 1.3 μg/ml, and 0.02 μg/ml for SARS-CoV, SARS-CoV-2, MERS-CoV, OC43, and PANG/GD, respectively. pp, pseudoparticles. (**H**) Alignment of betacoronavirus stem helix amino acid sequences with the S2P6 epitope boxed. Residue numbering is shown according to SARS-CoV-2 S. The sequences of the peptides used in this study are shown in blue, and N-linked glycosylation sequons are highlighted in yellow. (**I**) Competition assay for S2P6 or B6 binding to the SARS-CoV-2 or SARS-CoV stem helix peptide (herein defined as SARS-CoV/-2). B6 binding in the presence of S2P6 (red line), B6 binding in the absence of S2P6 (black line), and the control lacking the SARS-CoV/-2 stem helix peptide (gray line) are shown. (**J**) Kinetics of S2P6 binding to a panel of biotinylated betacoronavirus stem helix peptides immobilized at the surface of biolayer interferometry biosensors.

To identify mAbs targeting highly conserved regions of the S glycoprotein, we interrogated human immunoglobulin G^+^ (IgG^+^) memory B cells from three COVID-19 convalescent donors. Five mAbs bound to the prefusion-stabilized S ectodomain trimers of SARS-CoV and SARS-CoV-2 (*sarbecovirus*), MERS-CoV (*merbecovirus*), and OC43 and HKU1 (*embecovirus*), but not to the human alphacoronaviruses 229E and NL63 (Fig. 1, A to C, and fig. S1, A and B). S2P6 and S2S43 mAbs were each derived from a separate donor and use VH1-46*01 and D5-12*01 genes, whereas P34D10, P34G12, and P34E3 mAbs were derived from a third donor, are clonally related, and use the VH3-30 gene (fig. S1A). These five mAbs share nucleotide sequence identities with their respective germline genes ranging from 86 to 97% (fig. S1A), which correspond to a higher level of mutations than that observed for RBD- and NTD-specific mAbs isolated early after SARS-CoV-2 infection or vaccination (*5*, *19*–*21*). The mAbs bound to all prefusion-stabilized human-infecting betacoronavirus S trimers (Fig. 1, A to C, and fig. S1B), and they all neutralize SARS-CoV-2 S vesicular stomatitis virus (VSV) pseudotypes (fig. S1C).

S2P6 was selected for further in-depth characterization because it exhibited the overall greatest cross-reactivity and neutralization breadth. S2P6 bound both prefusion and postfusion SARS-CoV-2 S with comparable avidities, which indicates that its epitope might be (at least partially) accessible in both conformational states (Fig. 1, A and B, and fig. S1D). S2P6 bound to all full-length SARS-CoV-2 S variants tested and to 25 S glycoproteins representative of all *sarbecovirus* clades (Fig. 1D and fig. S1, E and F). Using surface plasmon resonance (SPR), we found that the S2P6 Fab fragment had the highest affinity for SARS-CoV-2 S and SARS-CoV S followed by MERS-CoV S and OC43 S with equilibrium dissociation constants (K_D_) of 7, 7, 12, and 16 nM, respectively (fig. S1, G and H). S2P6 also bound to HKU1 S, albeit with reduced affinity (K_D_ ~120 nM) (fig. S1G). Collectively, these data demonstrate that S2P6 cross-reacts with all human-infecting betacoronaviruses.

To evaluate the neutralization potency and breadth of S2P6, we investigated its ability to inhibit entry of authentic SARS-CoV-2 into Vero-E6 cells in the presence or absence of TMPRSS2, as this protease activates fusion with the cytoplasmic membrane in cultured lung cells (*22*–*24*). S2P6 completely neutralized infection of TMPRSS2-positive Vero-E6 cells but was less effective in inhibiting infection of Vero-E6 cells lacking TMPRSS2 (Fig. 1E), which suggests that S2P6 binding might be reduced at endosomal pH. The recognition of prefusion SARS-CoV-2 S by S2P6 is pH dependent, with higher binding affinity at pH 7, relative to pH 5, in both IgG and Fab formats (fig. S1H). We subsequently assessed S2P6-mediated neutralization of VSV (*25*) pseudotyped with SARS-CoV-2 S of several variants of concern (VOCs), including B.1.1.7 (Alpha), B.1.351 (Beta), P.1 (Gamma), and B1.617.1 (Kappa), and we observed similar potency to that found against the parental SARS-CoV-2 D614G S (Fig. 1F and fig. S1I). Moreover, S2P6 inhibited SARS-CoV S, Pangolin Guangdong 2019 (PANG/GD) S, MERS-CoV S, and OC43 S VSV pseudotypes with median inhibitory concentration (IC_50_) values ranging from 0.02 to 17 μg/ml (Fig. 1G). S2P6 is therefore a broadly neutralizing betacoronavirus mAb with activity against SARS-CoV-2 and SARS-CoV–related viruses as well as members of the *merbecovirus* and *embecovirus* subgenera.

Peptide mapping experiments using 15-nucleotide oligomer linear overlapping peptides revealed that all five mAbs bound to peptides containing the SARS-CoV-2 motif F_1148_KEELDKYF_1156_ located in the S_2_ subunit stem helix (Fig. 1H and fig. S2A). This region is strictly conserved in SARS-CoV, is highly conserved among other betacoronaviruses, and overlaps with the epitopes of the B6 (Fig. 1I) and 28D9 mouse mAbs (*14*, *15*). S2P6 bound efficiently to the stem helix peptides of the five betacoronaviruses that infect humans (albeit with a faster off-rate for HKU1) as well as those of the MERS-CoV–related bat viruses (HKU4 and HKU5) and murine hepatitis virus (MHV) (Fig. 1J and fig. S2B). S2S43 exhibited similar overall binding to that of S2P6, with markedly weaker reactivity toward the HKU1, HKU4, and HKU5 peptides, whereas the three clonally related P34D10, P34G12, and P34E3 mAbs exhibited weaker or no binding to HKU4 and HKU5 peptides (fig. S2B).

### Structural basis for S2P6 binding to the conserved S glycoprotein stem helix

**Fig. 2.**
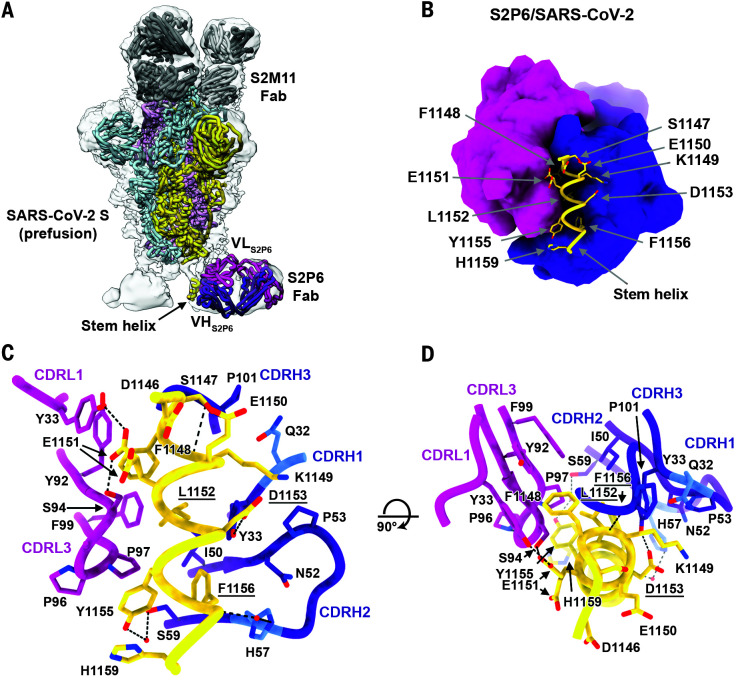
Structural basis for the broad S2P6 cross-reactivity with the conserved coronavirus stem helix peptide. (**A**) Composite model of the S2P6-bound SARS-CoV-2 S cryo-EM structure and of the S2P6-bound stem helix peptide crystal structure docked in the cryo-EM map (semitransparent gray surface). SARS-CoV-2 S protomers are colored pink, cyan, and gold; the S2P6 Fab heavy and light chains are colored purple and magenta; and the S2M11 Fab heavy and light chains are colored dark and light gray, respectively. (**B**) Crystal structure of the S2P6 Fab (surface rendering) in complex with the SARS-CoV-2 S stem helix peptide (yellow ribbon with side chains rendered as sticks). (**C** and **D**) Ribbon diagram in two orthogonal orientations of the S2P6 Fab bound to the SARS-CoV-2 S stem helix peptide showing the network of interactions. Only key interface residues and the S2P6 CDR loops are shown for clarity. Residues Q32 and H57, which are mutated during affinity maturation of the S2P6 heavy chain, are colored blue. Hydrogen bonds are indicated with dashed lines. Residues substituted in the escape mutants isolated are underlined. Single-letter abbreviations for the amino acid residues are as follows: A, Ala; C, Cys; D, Asp; E, Glu; F, Phe; G, Gly; H, His; I, Ile; K, Lys; L, Leu; M, Met; N, Asn; P, Pro; Q, Gln; R, Arg; S, Ser; T, Thr; V, Val; W, Trp; and Y, Tyr.

To determine the molecular basis of the S2P6 neutralization breadth, we determined a cryo–electron microscopy (cryo-EM) structure of the SARS-CoV-2 S ectodomain trimer in complex with the Fab fragments of S2P6 and S2M11 [to lock the RBDs in the closed state (*21*)] at 4.2-Å overall resolution. The marked conformational dynamics of the region recognized by S2P6 limited the local resolution of the stem helix–Fab to ~12 Å, and three-dimensional classification of the cryo-EM data revealed incomplete Fab saturation (Fig. 2A; table S1; and fig. S3, A to F). Our cryo-EM structure confirms that S2P6 recognizes the stem helix and suggests that the mAb disrupts its quaternary structure, which is presumed to form a three-helix bundle in prefusion SARS-CoV-2 S (*2*, *3*, *14*). To overcome the limited resolution of the stem helix–Fab interface in the cryo-EM structure, we determined a crystal structure of the S2P6 Fab in complex with the SARS-CoV-2 S stem helix peptide (residues 1146 to 1159) at 2.67-Å resolution (Fig. 2, B to D; fig. S4A; and table S2). The peptide folds as an amphipathic α helix resolved for residues 1146 to 1159. S2P6 buries ~600 Å^2^ upon binding to its epitope using shape-complementarity and hydrogen bonding involving complementarity-determining regions (CDRs) H1 to H3, L1, and L3. The light chain residues Y33, Y92, G93, S94, P96, P97, and F99 as well as heavy chain residues Y33, I50, H57, T58, S59, P101, K102, and G103 form a deep groove in which the hydrophobic side of the stem helix docks through residues F1148, L1152, Y1155, and F1156. Binding specificity is provided through backbone hydrogen bonding of residues F1148_SARS-CoV-2_ and K1149_SARS-CoV-2_ with CDRH3 P101 and side chain hydrogen bonding of residues E1151_SARS-CoV-2_ with CDRL1 Y33 and CDRL3 S94, D1153_SARS-CoV-2_ with CDRH1 Y33 side chains, as well as Y1155_SARS-CoV-2 S_ with CDRH2 S59 through a water molecule and F1156_SARS-CoV-2 S_ with CDRH2 H57, also through a water molecule (Fig. 2, C and D). The contribution of each epitope residue was validated by single-substitution scan analysis with most mutations at positions 1148, 1151 to 1153, and 1155 to 1156 abolishing S2P6 binding, which highlights the importance of these residues for mAb recognition (fig. S5A). A substitution scan analysis performed on the P34D10, P34G12, and P34E3 mAbs revealed a similar pattern of key interacting residues (fig. S5A). Residue Y1155 is conservatively substituted to F1238_MERS-CoV_ or W1237/1240_HKU1/OC43_, and residue D1153 is conserved in MERS-CoV and OC43 but mutated to S1235 for HKU1 (fig. S5B). The residue scan and structural results suggest that the reduced binding affinity of S2P6 for HKU1 S is at least partially a result of the D1153 _SARS-CoV-2_–S1235_HKU1_ substitution, which is expected to dampen electrostatic interactions with the CDRH1 Y33 side chain hydroxyl (Fig. 2, C and D).

Although S2P6 and B6 recognize a similar epitope (fig. S4, B and C), they bind with opposite orientations of the heavy and light chains relative to the stem helix and the S2P6-bound structure resolves three additional C-terminal peptide residues (1146 to 1159) compared with the B6-bound structure (1147 to 1156) (fig. S4, B and C). Superposition of both structures based on the stem helix reveals that B6 CDRH2 would sterically clash with H1159_SARS-CoV-2_, putatively explaining the broader cross-reactivity of S2P6 over B6 (fig. S4, B and C).

To further validate our structural data, we carried out viral escape mutant selection in vitro in the presence of S2P6 using a replication-competent VSV–SARS-CoV-2 S virus (*26*). After two passages, virus neutralization by S2P6 was abrogated, and deep sequencing revealed the emergence of five distinct resistance mutations—L1152F, D1153N/G/A, and F1156L, which are consistent with the structural data and substitution scan analysis (fig. S5A). Although these mutations have been detected with very low frequencies in circulating SARS-CoV-2 isolates (146 out of 1,217,814 sequences as of 30 April 2021), the subdominant immunogenicity of this cryptic site, likely because of its limited exposure, might result in low Ab pressure limiting accumulation of mutations in this epitope.

### Stem helix–targeting mAbs inhibit S-mediated membrane fusion

The S stem helix forms a three-helix bundle in many prefusion cryo-EM structures of SARS-CoV-2 S and SARS-CoV S although it is not fully resolved (*2*, *3*, *11*, *27*–*29*). By contrast, the S2P6–S2M11–SARS-CoV-2 S cryo-EM structure suggests that the quaternary organization of the stem is disrupted (Fig. 2A), consistent with S2P6 binding to the hydrophobic face of the stem helix. This face is mostly buried through homo-oligomeric interactions in prefusion S and may be only transiently available for Ab binding. Although we observed S2P6 binding to postfusion SARS-CoV-2 S (fig. S1D), the epitope recognized is buried at the interface with the other two protomers of the rod-shaped trimer and therefore might become accessible as a result of conformational dynamics, as is the case for the prefusion state (fig. S4D) (*14*, *30*–*32*). On the basis of these data, we propose that S2P6 binding to S sterically interferes with the conformational changes leading to membrane fusion, as observed for B6 (*14*) and 27D9 (*15*).

**Fig. 3.**
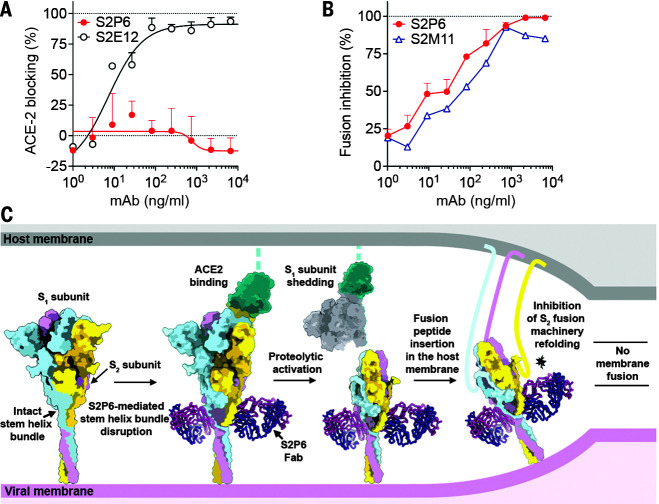
S2P6 binding disrupts the stem helix bundle and sterically inhibits membrane fusion. (**A**) SARS-CoV-2 S binding to ACE2 in the presence of mAb S2P6 analyzed by ELISA. S2E12 was included as a positive control. (**B**) S2P6 inhibition of cell-cell fusion using VeroE6 cells transfected with SARS-CoV-2 S. S2M11 was included as a positive control. Inhibition of fusion values are normalized to the percentage of fusion without mAb (100%) and to that of fusion of nontransfected cells (0%). (**C**) Proposed mechanism of inhibition mediated by the S2P6 mAb. S2P6 binds to the hydrophobic core of the stem helix bundle and disrupts its quaternary structure. S2P6 binding likely prevents S_2_ subunit refolding from the pre- to the postfusion state and blocks viral entry.

To validate the inferred mechanism of S2P6-mediated broad coronavirus neutralization, we first showed that S2P6 binding did not block engagement of SARS-CoV-2 S by angiotensin-converting enzyme 2 (ACE2)—using enzyme-linked immunosorbent assay (ELISA)—as expected based on the distance of its epitope from the RBD (Fig. 3A). S2P6, however, blocked cell-cell fusion between Vero-E6 cells transfected with full-length SARS-CoV-2 S as effectively as the S2M11 mAb, which locks SARS-CoV-2 S in the closed state (*21*) (Fig. 3B). We previously described mAbs targeting RBD antigenic sites Ia (e.g., S2E12) and IIa (e.g., S2X259 or S2X35), which can mimic receptor attachment and prematurely trigger fusogenic S conformational changes (*11*, *20*, *33*). Accordingly, S2P6 at concentrations as low as 1 ng/ml abrogated the formation of syncytia mediated by S2E12 (*33*). Collectively, these results suggest that the main mechanism of S2P6 neutralization is to prevent viral entry by impeding S fusogenic rearrangements, thereby inhibiting membrane fusion (Fig. 3C).

### S2P6-mediated protection in hamsters is enhanced by Fc-mediated effector functions

**Fig. 4.**
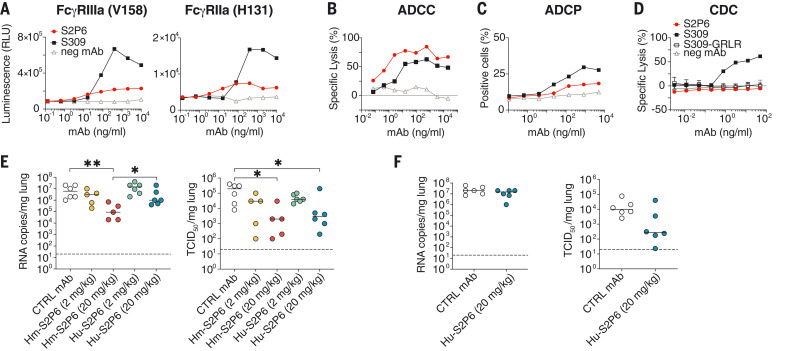
S2P6 activates effector functions and reduces SARS-CoV-2 lung burden in Syrian hamsters. (**A**) NFAT (nuclear factor of activated T cells)–driven luciferase signal induced in Jurkat cells stably expressing FcγRIIIa (V158, left) or FcγRIIa (V131, right) upon S2P6 binding to full-length wild-type SARS-CoV-2 S expressed at the surface of CHO target cells. S309 is included as positive control. RLU, relative luminescence unit. (**B**) mAb-mediated ADCC using SARS-CoV-2 CHO-K1 cells (genetically engineered to stably express a HaloTag-HiBit) as target cells and PBMC as effector cells. The magnitude of natural killer cell–mediated killing is expressed as a percentage of specific lysis. (**C**) mAb-mediated ADCP using cell trace violet–labeled PBMCs as a source of phagocytic cells (monocytes) and PKH67–fluorescently labeled S-expressing CHO cells as target cells. The *y* axis indicates the percentage of monocytes double-positive for anti-CD14 (monocyte) marker and PKH67. (**D**) Lysis of SARS-CoV-2 S stably transfected CHO cells by mAbs in the presence of complement. S309 was included as positive control; S309-GRLR with diminished FcγR binding capacity and an unrelated mAb (neg mAb) were used as negative controls. (**E**) Syrian hamsters were administered with the indicated amount of S2P6 mAb harboring either a hamster (Hm-S2P6) or a human (Hu-S2P6) constant region before intranasal challenge with prototypic SARS-CoV-2 (Wuhan-1 related). An irrelevant mAb (MGH2 against *Plasmodium falciparum* CSP) at 20 mg/kg was used as negative control (CTRL) (*60*). Viral RNA loads and replicating virus titers are shown on the left and right, respectively. TCID_50_, median tissue culture infectious dose. (**F**) Prophylactic administration of Hu-S2P6 at 20 mg/kg in hamsters challenged with SARS-CoV-2 B.1.351 (Beta) VOC. Viral RNA loads and replicating virus titers are shown on the left and right, respectively. *P < 0.05; **P < 0.01; Mann-Whitney test.

Functions mediated by the constant (Fc) region of Abs can contribute to in vivo protection by promoting viral clearance and antiviral immune responses (*34*–*37*). We analyzed the ability of S2P6 to trigger activation of the Fc receptors, FcγRIIa and FcγRIIIa, as well as to exert Fc effector functions in vitro. S2P6 promoted moderate dose-dependent FcγRIIa- and FcγRIIIa-mediated signaling using a luciferase reporter assay (Fig. 4A). However, S2P6 promoted robust activation of Ab-dependent cell cytotoxicity (ADCC), to levels comparable to those observed with the S309 mAb (*38*), after incubation of SARS-CoV-2 S–expressing CHO-K1 target cells with human peripheral blood mononuclear cells (PBMCs) (Fig. 4B). S2P6 also triggered measurable Ab-dependent cellular phagocytosis (ADCP) activity using cell trace violet–labeled PBMCs as phagocytic cells and SARS-CoV-2 S–expressing CHO (CHO-S) as target cells (Fig. 4C). Finally, S2P6 did not promote complement-dependent cytotoxicity (CDC) (Fig. 4D), indicating that S2P6 Fc–mediated effector functions, but not CDC, might participate in viral control in vivo.

We evaluated the prophylactic activity of S2P6 against challenge with the prototypic (Wuhan-1 related) SARS-CoV-2 in a Syrian hamster model (*39*). As we previously showed that the human IgG1 Fc fragment poorly recognizes hamster FcγRs (*33*), we compared S2P6 harboring a human IgG1 constant region (Hu-S2P6) with S2P6 harboring a hamster IgG2a constant region (Hm-S2P6), the latter enabling optimal interactions with hamster FcγRs. Two different doses of Hu-S2P6 or Hm-S2P6 were administered 24 hours before intranasal SARS-CoV-2 challenge, and the lungs of the animals were assessed 4 days after infection for viral RNA load and replicating virus. Hm-S2P6 administered at 20 mg/kg reduced lung viral RNA copies and replicating viral titers by two orders of magnitude relative to a control mAb but did not exert any antiviral effect at 2 mg/kg (Fig. 4E and fig. S6). Hm-S2P6 at 20 mg/kg reduced lung viral RNA load to levels statistically significantly lower than those observed with Hu-S2P6 at 20 mg/kg, suggesting a beneficial effect of S2P6 effector functions in vivo. On the basis of the comparable S2P6 neutralization potencies toward SARS-CoV-2 VOCs observed in vitro (Fig. 1F), we assessed the protective efficacy of S2P6 in hamsters challenged with SARS-CoV-2 B.1.351 (Beta VOC). Prophylactic administration of Hu-S2P6 at 20 mg/kg reduced replicating viral titers in the lungs (but not RNA copy numbers) by ~1.5 orders of magnitude relative to the control group. Although this difference was not statistically significant, the observed efficacy against this VOC is in line with the strict conservation of the stem helix epitope in all VOCs identified to date (Fig. 4F). Collectively, these findings demonstrate that Abs targeting a highly conserved epitope in the S fusion machinery can trigger Fc-mediated ADCC and ADCP in vitro and that their in vivo antiviral activity may rely on both neutralization and effector functions.

### Natural infection or vaccination predominantly elicit stem helix–directed Abs of narrow specificities

**Fig. 5.**
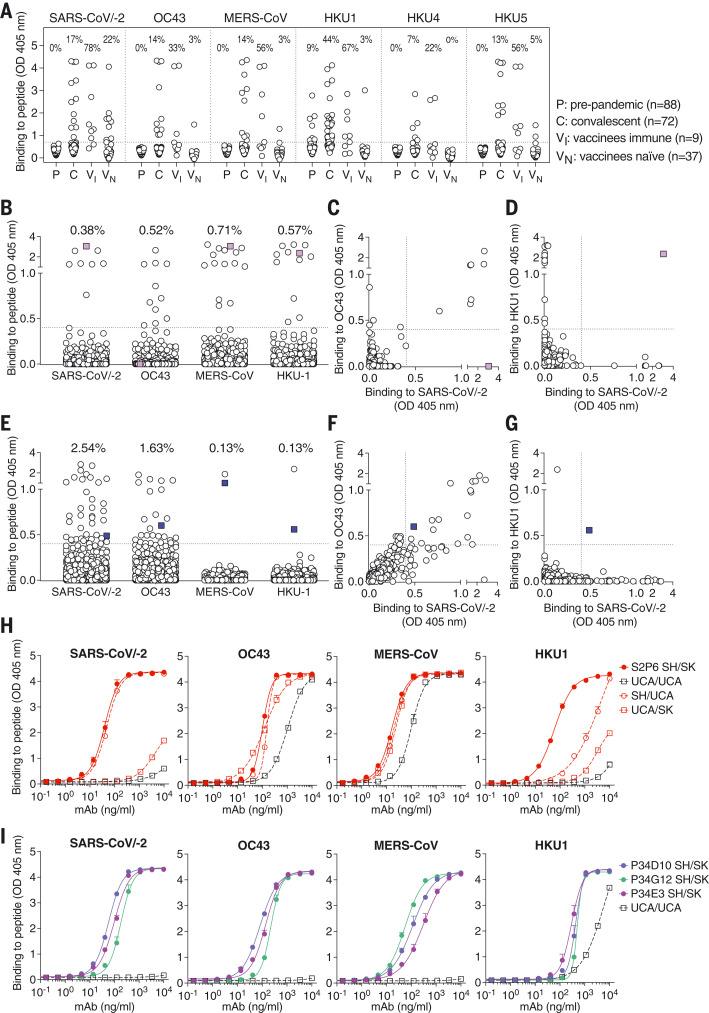
Stem helix–directed Abs are rare, of narrow specificities, and enhance their binding affinity and cross-reactivity through somatic mutations. (**A**) Binding of prepandemic (P; *n* = 88), COVID-19 convalescent (C; *n* = 72), vaccinees immune (VI; *n* = 9), and vaccinees naïve (VN; *n* = 37) plasma Abs (diluted 1:10) to immobilized betacoronavirus stem helix peptides analyzed by ELISA. A cutoff of 0.7 was determined on the basis of the signal of prepandemic samples and binding to uncoated ELISA plates (horizontal dashed line). The fraction of samples for which binding above the cutoff was detected is indicated. (**B** to **G**) Analysis of memory B cell specificities for betacoronavirus stem helix peptides. Each dot represents a well containing oligoclonal B cell supernatant screened for the presence of stem helix peptide binding IgG Abs using ELISA. Samples obtained from 21 COVID-19 convalescent individuals [(B) to (D)] and 16 vaccinees [(E) to (G)]. Pairwise reactivity comparison is shown for SARS-CoV/-2 and OC43 [(C) and (F)] and SARS-CoV/-2 and HKU1 [(D) and (G)]. Cultures cross-reactive with at least three peptides are highlighted in color. A cutoff of 0.4 is indicated by a horizontal dashed line. The fraction of wells for which binding above the cutoff was detected is indicated. (**H** and **I**) Binding to stem helix peptides of S2P6 (H) harboring mature (SH/SK), fully germline-reverted (UCA/UCA), germline-reverted heavy chain paired with mature light chain (UCA/SK), mature heavy chain paired with germline-reverted light chain (SH/UCA), and of P34D10, P34G12, and P34E3 (I) harboring either mature (SH/SK) or germline-reverted (UCA/UCA) sequences.

To understand how frequently stem helix–specific Abs are elicited, we analyzed plasma samples from prepandemic, COVID-19–convalescent, and COVID-19–vaccinated individuals to determine IgG binding titers to the SARS-CoV-2–SARS-CoV (SARS-CoV/-2), OC43, MERS-CoV, HKU1, HKU4, and HKU5 stem helix peptides (Fig. 5A and fig. S7). In prepandemic samples, we only observed binding to the HKU1 stem helix peptide, probably reflecting prior infection with this virus in the cohort. Stem helix–specific Abs were found at low frequencies in individuals previously infected with SARS-CoV-2 or those who had received two doses of mRNA vaccines (Fig. 5A). The highest frequencies of stem helix–specific serum Abs were observed for vaccinated individuals who were previously infected (ranging from 22 to 78% against the different stem helix peptides). Overall, these data show that plasma Ab responses to the stem helix are elicited upon SARS-CoV-2 infection or vaccination, although they are relatively rare. Evaluation of the prevalence of mAbs targeting other fusion machinery epitopes will reveal whether this trend holds true for the overall S_2_ subunit or whether it is a specificity of the cryptic stem helix epitope.

Next, we investigated the frequencies of stem helix–specific memory B cells among 21 convalescent and 17 vaccinated individuals using a clonal analysis based on in vitro polyclonal stimulation (*40*) (Fig. 5, B to G, and figs. S7 to S9). In both cohorts, we observed frequencies of culture positive for stem helix–specific IgGs ranging from 0 to 2.5%, except for one individual (infected and vaccinated with a single dose of mRNA vaccine) for which 99.6% of culture supernatants were binding to the SARS-CoV/-2 stem helix (fig. S9). Most SARS-CoV-2 stem helix–specific memory B cells were cross-reactive with OC43, consistent with the high sequence identity of the stem helices of these two viruses (Fig. 5, C and F, and Fig. 1H). Abs specific for the HKU1 S stem helix were found, but they were not cross-reactive with other betacoronaviruses except for one convalescent and one vaccinated individual (Fig. 5, D and G). This analysis revealed a single example of cross-reactivity to all stem helix betacoronavirus peptides tested (Fig. 5E), whereas most other Abs show limited cross-reactivity among betacoronaviruses.

### Broadly reactive betacoronavirus Abs enhance their binding affinity and cross-reactivity through somatic mutations

To define the ontogeny of the broadly reactive betacoronavirus mAbs presented here, we generated a panel of germline variants of S2P6, P34D10, P34E3, and P34G12. Two out of seven S2P6 heavy chain residues that are mutated relative to germline contribute to epitope recognition (Q32 and H57), whereas none of the five light chain mutated residues participate in S binding (Fig. 2, C and D). To address the role of VH and VK somatic mutations, we generated a panel of S2P6 germline variants for the heavy or the light chain, or both variable regions (VH and VK). The fully germline-reverted S2P6 [unmutated common ancestor (UCA)] bound to OC43 and MERS-CoV stem helix peptides [with approximately one order of magnitude higher median effective concentration (EC_50_) compared with that of the mature mAb] but did not bind to SARS-CoV/-2 or HKU1 stem helix peptides or spike trimers (Fig. 5H and fig. S10, A to C). Somatic mutations in VH were sufficient for high-avidity binding to SARS-CoV/-2, whereas both VH and VK mutations were required for optimal binding to HKU1. The presence of residue G103 in CDRH3 was essential for binding to all betacoronaviruses (fig. S10, A to C). These findings indicate that the S2P6 mAb likely arose in response to OC43 infection, and its specificity was broadened toward SARS-CoV-2 and HKU1 through somatic mutations selected upon natural infection with one or both of these betacoronaviruses. By contrast, analysis of UCA binding of the clonally related P34D10, P34G12, and P34E3 mAbs suggests that they were likely primed by HKU1 infection rather than OC43 infection and acquired cross-reactivity toward the other human betacoronaviruses primarily through somatic mutations in VH (Fig. 5I and fig. S10, D to F). Collectively, these findings demonstrate that broadly reactive betacoronavirus Abs may result from priming of virus-specific B cells gaining affinity and cross-reactivity through somatic mutations in response to heterotypic coronavirus exposures.

## Discussion

The coronavirus S_2_ subunit (fusion machinery) contains several conserved antigenic sites, including the fusion peptide and the heptad-repeat 2 regions (*41*–*46*). The recent identification of four cross-reactive mAbs targeting the stem helix in the S_2_ subunit highlighted the importance of this epitope, which is conserved among betacoronavirus but not alphacoronavirus S glycoproteins (*14*–*16*, *47*), although none of these mAbs inhibit members of all three betacoronavirus subgenera. Here, we identified five stem helix–specific human mAbs cross-reacting with human and animal betacoronaviruses and showed that S2P6 broadly neutralized all sarbecoviruses, merbecoviruses, and embecoviruses pseudotypes evaluated through the inhibition of membrane fusion.

We show that an S_2_ subunit–directed mAb reduces lung viral burden in hamsters challenged with SARS-CoV-2 with a key contribution of Fc-mediated effector functions, as described for some SARS-CoV-2 RBD-specific mAbs (*34*, *37*) and influenza virus hemagglutinin stem mAbs (*17*, *48*, *49*). Although this study showcases the stem helix as a target of pan-betacoronavirus Abs, the existence of other cross-reactive S_2_ epitopes with neutralizing potential remains a subject of investigation. Stem helix–targeted Abs are elicited upon natural infection by endemic (OC43 or HKU1) or pandemic (SARS-CoV-2) coronaviruses as well as by COVID-19 mRNA vaccines. Similarly to the subdominance of Abs recognizing the conserved hemagglutinin stem region of influenza A viruses (*48*, *50*), stem helix–specific Abs are present at low titers in convalescent or vaccinee plasma and at low frequencies in their memory B cell repertoires, possibly as a result of limited epitope exposure. These findings along with the moderate neutralization potency of these mAbs indicate that eliciting high-enough titers of stem helix–targeted mAbs through vaccination will be a key challenge to overcome to develop pan-betacoronavirus vaccines. We propose that harnessing recent advances in computational protein design, such as epitope-focused vaccine design approaches as described for the respiratory syncytial virus fusion protein (*51*–*53*) and multivalent display as described for influenza virus (*54*–*59*) to target the S stem helix or the fusion peptide regions, might enable elicitation of broad betacoronavirus immunity.

## Supplementary Material

20210803-1

## Supplementary Materials

### This PDF file includes:

Materials and Methods Figs. S1 to S10 Tables S1 and S2 References (*61*–*86*)

### Other Supplementary Material for this manuscript includes the following:

MDAR Reproducibility Checklist

## Notes

View/request a protocol for this paper from *Bio-protocol*.
