# Supplementary material for: Broad betacoronavirus neutralization by a stem helix–specific human antibody
Source: Science. 2021 Aug 3;373(6559):1109–16. doi: 10.1126/science.abj3321 (PMC9268357; doi:10.1126/science.abj3321)
Supplement: 20210803-1 [file science.abj3321.v1.pdf]

Cite as: D. Pinto *et al.*, *Science* 10.1126/science.abj3321 (2021).

# Broad betacoronavirus neutralization by a stem helix-specific human antibody

**Dora Pinto[1]†, Maximilian M. Sauer[2]†, Nadine Czudnochowski[3]†, Jun Siong Low[4]†, M. Alejandra Tortorici[2], Michael P. Housley[3], Julia Noack[3], Alexandra C. Walls[2], John E. Bowen[2], Barbara Guarino[1], Laura E. Rosen[3], Julia di Iulio[3], Josipa Jerak[4], Hannah Kaiser[3], Saiful Islam[3], Stefano Jaconi[1], Nicole Sprugasci[1], Katja Culap[1], Rana Abdelnabi[5], Caroline Foo[5], Lotte Coelmont[5], Istvan Bartha[1], Siro Bianchi[1], Chiara Silacci-Fregni[1], Jessica Bassi[1], Roberta Marzi[1], Eneida Vetti[1], Antonino Cassotta[4], Alessandro Ceschi[6,7,8,9], Paolo Ferrari[9,10,11], Pietro E. Cippà[10,12], Olivier Giannini[9,10], Samuele Ceruti[13], Christian Garzoni[14], Agostino Riva[15], Fabio Benigni[1], Elisabetta Cameroni[1], Luca Piccoli[1], Matteo S. Pizzuto[1], Megan Smithey[3], David Hong[3], Amalio Telenti[3], Florian A. Lempp[3], Johan Neyts[5], Colin Havenar-Daughton[3], Antonio Lanzavecchia[1], Federica Sallusto[4,16], Gyorgy Snell[3], Herbert W. Virgin[3,17,18], Martina Beltramello[1], Davide Corti[1]*, David Veesler[2]***

[1]Humabs Biomed SA, a subsidiary of Vir Biotechnology, 6500 Bellinzona, Switzerland. [2]Department of Biochemistry, University of Washington, Seattle, WA 98195, USA. [3]Vir Biotechnology, San Francisco, CA 94158, USA. [4]Institute for Research in Biomedicine, Università della Svizzera italiana, 6500 Bellinzona, Switzerland. [5]Rega Institute for Medical Research, Laboratory of Virology and Chemotherapy, KU Leuven, 3000 Leuven, Belgium. [6]Clinical Trial Unit, Ente Ospedaliero Cantonale, 6900 Lugano, Switzerland. [7]Division of Clinical Pharmacology and Toxicology, Institute of Pharmacological Sciences of Southern Switzerland, Ente Ospedaliero Cantonale, 6900 Lugano, Switzerland. [8]Department of Clinical Pharmacology and Toxicology, University Hospital Zurich, 8091 Zurich, Switzerland. [9]Faculty of Biomedical Sciences, Università della Svizzera italiana, 6900 Lugano, Switzerland. [10]Department of Medicine, Ente Ospedaliero Cantonale, 6500 Bellinzona, Switzerland. [11]Clinical School, University of New South Wales, Sydney, NSW, 2052, Australia. [12]Faculty of Medicine, University of Zurich, 8057 Zurich, Switzerland. [13]Intensive Care Unit, Clinica Luganese Moncucco, 6900 Lugano, Switzerland. [14]Clinic of Internal Medicine and Infectious Diseases, Clinica Luganese Moncucco, 6900 Lugano, Switzerland. [15]III Division of Infectious Diseases, ASST Fatebenefratelli Sacco, Luigi Sacco Hospital, 20157 Milan, Italy. [16]Institute of Microbiology, ETH Zurich, 8093 Zurich, Switzerland. [17]UT Southwestern Medical Center, Dallas, TX 75390, USA. [18]Washington University School of Medicine, St. Louis, MO 63110, USA.

†These authors contributed equally to this work.

***Corresponding author. Email: dveesler@uw.edu (D.V.); dcorti@vir.bio (D.C.)**

**The spillovers of β-coronaviruses in humans and the emergence of SARS-CoV-2 variants highlight the need for broad coronavirus countermeasures. We describe five monoclonal antibodies (mAbs) cross-reacting with the stem helix of multiple β-coronavirus spike glycoproteins isolated from COVID-19 convalescent individuals. Using structural and functional studies we show that the mAb with the greatest breadth (S2P6) neutralizes pseudotyped viruses from three different subgenera through inhibition of membrane fusion and delineate the molecular basis for its cross-reactivity. S2P6 reduces viral burden in hamsters challenged with SARS-CoV-2 through viral neutralization and Fc-mediated effector functions. Stem helix antibodies are rare, oftentimes of narrow specificity and can acquire neutralization breadth through somatic mutations. These data provide a framework for structure-guided design of pan-β-coronavirus vaccines eliciting broad protection.**

Coronaviruses are zoonotic pathogens responsible for several epidemics and a pandemic in the past two decades. All three highly pathogenic coronaviruses belong to the β-coronavirus genus: SARS-CoV-2 and SARS-CoV cluster within the *sarbecovirus* subgenus and originated in bats whereas MERS-CoV belongs to the *merbecovirus* subgenus and is transmitted to humans through dromedary camels. Moreover, HCoV-HKU1 and HCoV-OC43 (*embecovirus* subgenus, β-coronaviruses) as well as NL63 and 229E (*setracovirus* and *duvinacovirus* subgenera, α-coronaviruses) are endemic and cause common colds in humans.

The coronavirus spike (S) glycoprotein promotes viral entry into host cells through an $S_1$ subunit which engages host receptors and an $S_2$ subunit mediating membrane fusion (*1*). The $S_1$ subunit is the major target of (neutralizing) antibodies (Abs), and is more genetically variable than the $S_2$ subunit (*2*–*5*). Accordingly, Abs binding to the $S_1$ subunit receptor-binding domain (RBD) and N-terminal domain (NTD) exert a selective pressure resulting in the emergence of new variants (*5*–*10*). SARS-CoV-2 S comprises 22 N-linked glycans (13 and 9 of them in the $S_1$ and $S_2$ subunits, respectively) participating in folding as well as modulating access to host proteases and antibodies (*1*, *2*, *11*–*13*). MAbs binding to the $S_2$ fusion machinery can neutralize distantly related coronaviruses (*14*–*16*) as observed for other viruses, such as influenza and HIV-1 (*17*, *18*).

## Results

### ***Isolation of a broadly neutralizing β-coronavirus mAb from a convalescent SARS-CoV-2-exposed individual***

To identify mAbs targeting highly conserved regions of the S glycoprotein, we interrogated human $IgG^+$ memory B cells from three COVID-19 convalescent donors. Five mAbs bound to the prefusion-stabilized S ectodomain trimers of SARS-CoV and SARS-CoV-2 (*sarbecovirus*), MERS-CoV (*merbecovirus*), OC43 and HKU1 (*embecovirus*), but not to the human α-coronaviruses 229E and NL63 (Fig. 1, A to C, and fig. S1, A and B). S2P6 and S2S43 mAbs were each derived from a separate donor and use VH1-46*01 and D5-12*01 genes whereas P34D10, P34G12 and P34E3 mAbs derived from a third donor, are clonally related and use the VH3-30 gene (fig. S1A). These 5 mAbs share nucleotide sequence identities with their respective germline genes ranging from 86 to 97% (fig. S1A), which correspond to a higher level of mutations than that observed for RBD- and NTD-specific mAbs isolated early after SARS-CoV-2 infection or vaccination (*5*, *19*–*21*). The mAbs bound to all prefusion-stabilized human-infecting β-coronavirus S trimers (Fig. 1, A to C, and fig. S1B) and they all neutralize SARS-CoV-2 S VSV pseudotypes (fig.S1C).

S2P6 was selected for further in-depth characterization because it exhibited the overall greatest cross-reactivity and neutralization breadth. S2P6 bound both prefusion and post-fusion SARS-CoV-2 S with comparable avidities, indicating that its epitope might be (at least partially) accessible in both conformational states (Fig. 1, A and B, and fig. S1D). S2P6 bound to all full-length SARS-CoV-2 S variants tested and to 25 S glycoproteins representative of all *sarbecovirus* clades (Fig. 1D and fig. S1, E and F). Using surface plasmon resonance (SPR), we found that the S2P6 Fab fragment had the highest affinity for SARS-CoV-2 S and SARS-CoV S followed by MERS-CoV S and OC43 S with equilibrium dissociation constants ($K_D$) of 7, 7, 12 and 16 nM, respectively (fig. S1, G and H). S2P6 also bound to HKU1 S albeit with reduced affinity ($K_D$ ~120 nM) (fig. S1G). Collectively, these data demonstrate that S2P6 cross-reacts with all human-infecting β-coronaviruses.

To evaluate the neutralization potency and breadth of S2P6, we investigated its ability to inhibit entry of authentic SARS-CoV-2 into Vero-E6 cells in the presence or absence of TMPRSS2 as this protease activates fusion with the cytoplasmic membrane in cultured lung cells (*22*–*24*). S2P6 completely neutralized infection of TMPRSS2-positive Vero-E6 cells but was less effective in inhibiting infection of Vero-E6 cells lacking TMPRSS2 (Fig. 1E), suggesting that S2P6 binding might be reduced at endosomal pH. Indeed, the recognition of prefusion SARS-CoV-2 S by S2P6 is pH dependent with higher binding affinity at pH 7, relative to pH 5, in both IgG and Fab formats (fig. S1H). We subsequently assessed S2P6-mediated neutralization of vesicular stomatitis virus (VSV) (*25*) pseudotyped with SARS-CoV-2 S of several variants of concern (VOC), including B.1.1.7 (alpha), B.1.351 (beta), P.1 (gamma) and B1.617.1 (kappa) and observed similar potency to that found against the parental SARS-CoV-2 D614G S (Fig. 1F and fig. S1I). Moreover, S2P6 inhibited SARS-CoV S, Pangolin Guangdong 2019 (PANG/GD) S, MERS-CoV S and OC43 S VSV pseudotypes with IC50 values ranging from 0.02 to 17 μg/ml (Fig. 1G). S2P6 is therefore a broadly neutralizing β-coronavirus mAb with activity against SARS-CoV-2 and SARS-CoV-related viruses as well as members of the merbecovirus and embecovirus subgenera.

Peptide mapping experiments using 15-mer linear overlapping peptides revealed that all five mAbs bound to peptides containing the SARS-CoV-2 motif $F_{1148}$KEELDKY$F_{1156}$ located in the $S_2$ subunit stem helix (Fig. 1H and fig. S2A) . This region is strictly conserved in SARS-CoV, highly conserved among other β-coronaviruses, and overlaps with the epitopes of the B6 (Fig. 1I) and 28D9 mouse mAbs (*14*, *15*). S2P6 bound efficiently to the stem helix peptides of the five β-coronaviruses that infect humans (albeit with a faster off-rate for HKU1) as well as of the MERS-CoV-related bat viruses (HKU4 and HKU5) and murine hepatitis virus (MHV) (Fig. 1J and fig. S2B). S2S43 exhibited similar overall binding than S2P6 with markedly weaker reactivity toward the HKU1, HKU4 and HKU5 peptides, whereas the three clonally related P34D10, P34G12, and P34E3 mAbs exhibited weaker or no binding to HKU4 and HKU5 peptides (fig. S2B).

### ***Structural basis for S2P6 binding to the conserved S glycoprotein stem helix***

To determine the molecular basis of the S2P6 neutralization breadth, we determined a cryo-EM structure of the SARS-CoV-2 S ectodomain trimer in complex with the Fab fragments of S2P6 and S2M11 (to lock the RBDs in the closed state (*21*)) at 4.2 Å overall resolution. The marked conformational dynamics of the region recognized by S2P6 limited the local resolution of the stem helix/Fab to approximately ~12 Å and 3D classification of the cryo-EM data revealed incomplete Fab saturation (Fig. 2A; table S1; and fig. S3, A to F). Our cryo-EM structure confirms that S2P6 recognizes the stem helix and suggests that the mAb disrupts its quaternary structure, which is presumed to form a 3-helix bundle in prefusion SARS-CoV-2 S (*2*, *3*, *14*). To overcome the limited resolution of the stem helix/Fab interface in the cryoEM structure, we determined a crystal structure of the S2P6 Fab in complex with the SARS-CoV-2 S stem helix peptide (residues 1146-1159) at 2.67 Å resolution (Fig. 2, B to D; fig. S4A; and table S2). The peptide folds as an amphipathic α-helix resolved for residues 1146 to 1159. S2P6 buries approximately 600 $Å^2$ upon binding to its epitope using shape-complementarity and hydrogen bonding involving complementarity determining

regions (CDR) H1-H3, L1 and L3. The light chain residues Y33, Y92, G93, S94, P96, P97 and F99 as well as heavy chain residues Y33, I50, H57, T58, S59, P101, K102 and G103 form a deep groove in which the hydrophobic side of the stem helix docks through residues F1148, L1152, Y1155 and F1156. Binding specificity is provided through backbone hydrogen bonding of residues $F1148_{SARS\text{-}CoV\text{-}2}$ and $K1149_{SARS\text{-}CoV\text{-}2}$ with CDRH3 P101, side chain hydrogen bonding of residues $E1151_{SARS\text{-}CoV\text{-}2}$ with CDRL1 Y33 and CDRL3 S94, $D1153_{SARS\text{-}CoV\text{-}2}$ with CDRH1 Y33 side chains as well as $Y1155_{SARS\text{-}CoV\text{-}2\ S}$ with CDRH2 S59 through a water molecule and $F1156_{SARS\text{-}CoV\text{-}2\ S}$ with CDRH2 H57 also through a water molecule (Fig. 2, C and D). The contribution of each epitope residue was validated by single substitution scan analysis with most mutations at positions 1148, 1151-1153 and 1155-1156 abolishing S2P6 binding, highlighting the importance of these residues for mAb recognition (fig. S5A). A substitution scan analysis performed on the P34D10, P34G12 and P34E3 mAbs revealed a similar pattern of key interacting residues (fig. S5A). Residue Y1155 is conservatively substituted to $F1238_{MERS\text{-}CoV}$ or $W1237/1240_{HKU1/OC43}$ and residue D1153 is conserved in MERS-CoV and OC43 but mutated to S1235 for HKU1 (fig. S5B). The residue scan and structural results suggest that the reduced binding affinity of S2P6 for HKU1 S is at least partially due to the $D1153_{SARS\text{-}CoV\text{-}2}/S1235_{HKU1}$ substitution which is expected to dampen electrostatic interactions with the CDRH1 Y33 side chain hydroxyl (Fig. 2, C and D).

Although S2P6 and B6 recognize a similar epitope (fig. S4, B and C), they bind with opposite orientations of the heavy and light chains relative to the stem helix and the S2P6-bound structure resolves 3 additional C-terminal peptide residues (1156-1159) compared to the B6-bound structure (1147-1156) (fig. S4, B and C). Superposition of both structures based on the stem helix reveals that B6 CDRH2 would sterically clash with $H1159_{SARS\text{-}CoV\text{-}2}$, putatively explaining the broader cross-reactivity of S2P6 over B6 (fig. S4, B and C).

To further validate our structural data, we carried out viral escape mutant selection in vitro in the presence of S2P6 using a replication-competent VSV-SARS-CoV-2 S virus (*26*). After two passages, virus neutralization by S2P6 was abrogated and deep sequencing revealed the emergence of five distinct resistance mutations: L1152F, D1153N/G/A and F1156L, which are consistent with the structural data and substitution scan analysis (fig. S5A). Although these mutations have been detected with very low frequencies in circulating SARS-CoV-2 isolates (146 out of 1,217,814 sequences as of April 30, 2021), the subdominant immunogenicity of this cryptic site, likely due to its limited exposure, might result in low Ab pressure limiting accumulation of mutations in this epitope.

### *Stem helix-targeting mAbs inhibit S-mediated membrane fusion*

The S stem helix forms a 3-helix bundle in many prefusion cryoEM structures of SARS-CoV-2 S and SARS-CoV S although it is not fully resolved (*2*, *3*, *11*, *27*–*29*). In contrast, the S2P6/S2M11/SARS-CoV-2 S cryoEM structure suggests that the quaternary organization of the stem is disrupted (Fig. 2A) consistent with S2P6 binding to the hydrophobic face of the stem helix. This face is mostly buried through homo-oligomeric interactions in prefusion S, and may be only transiently available for Ab binding. Although we observed S2P6 binding to post-fusion SARS-CoV-2 S (fig. S1D), the epitope recognized is buried at the interface with the other two protomers of the rod-shaped trimer and therefore might become accessible due to conformational dynamics, as is the case for the prefusion state (fig. S4D) (*14*, *30*–*32*). Based on these data, we propose that S2P6 binding to S sterically interferes with the conformational changes leading to membrane fusion, as observed for B6 (*14*) and 27D9 (*15*).

To validate the inferred mechanism of S2P6-mediated broad coronavirus neutralization, we first showed that S2P6 binding did not block engagement of SARS-CoV-2 S by ACE2 using ELISA, as expected based on the distance of its epitope from the RBD (Fig. 3A). S2P6, however, blocked cell-cell fusion between Vero-E6 cells transfected with full-length SARS-CoV-2 S as effectively as the S2M11 mAb which locks SARS-CoV-2 S in the closed state (*21*) (Fig. 3B). We previously described mAbs targeting RBD antigenic site Ia (e.g., S2E12) and IIa (e.g., S2X259 or S2X35) which can mimic receptor attachment and prematurely trigger fusogenic S conformational changes (*11*, *20*, *33*). Accordingly, S2P6 at concentrations as low as 1 ng/ml abrogated the formation of syncytia mediated by S2E12 (*33*). Collectively, these results suggest that the main mechanism of S2P6 neutralization is to prevent viral entry by impeding S fusogenic rearrangements thus inhibiting membrane fusion (Fig. 3C).

### *S2P6-mediated protection in hamsters is enhanced by Fc-mediated effector functions*

Functions mediated by the constant (Fc)-region of Abs can contribute to in vivo protection by promoting viral clearance and anti-viral immune responses (*34*–*37*). We analyzed the ability of S2P6 to trigger activation of the Fc receptors, FcγRIIa and FcγRIIIa, as well as to exert Fc effector functions in vitro. S2P6 promoted moderate dose-dependent FcγRIIa and FcγRIIIa mediated signaling using a luciferase reporter assay (Fig. 4A). However, S2P6 promoted robust activation of Ab-dependent cell cytotoxicity (ADCC), to levels comparable to those observed with the S309 mAb (*38*), following incubation of SARS-CoV-2 S-expressing CHO-K1 target cells with human peripheral blood mononuclear cells (PBMCs) (Fig. 4B).

S2P6 also triggered measurable Ab-dependent cellular phagocytosis (ADCP) activity using Cell-Trace-Violet-labeled PBMCs as phagocytic cells, and SARS-CoV-2 S-expressing CHO (CHO-S) as target cells (Fig. 4C). Finally, S2P6 did not promote complement-dependent cytotoxicity (CDC) (Fig. 4D) indicating that S2P6 Fc-mediated effector functions, but not CDC, might participate in viral control in vivo.

We evaluated the prophylactic activity of S2P6 against challenge with the prototypic (Wuhan-1 related) SARS-CoV-2 in a Syrian hamster model (*39*). As we previously showed that the human IgG1 Fc fragment poorly recognizes hamster FcγRs (*33*), we compared S2P6 harboring a human IgG1 constant region (Hu-S2P6) with S2P6 harboring a hamster IgG2a constant region (Hm-S2P6), the latter enabling optimal interactions with hamster FcγRs. Two different doses of Hu-S2P6 or Hm-S2P6 were administered 24 hours prior to intranasal SARS-CoV-2 challenge and the lungs of the animals were assessed 4 days post-infection for viral RNA load and replicating virus. Hm-S2P6 administered at 20 mg/kg reduced lung viral RNA copies and replicating viral titers by two orders of magnitude relative to a control mAb but did not exert any antiviral effect at 2 mg/kg (Fig. 4E and fig. S6). Hm-S2P6 at 20 mg/kg reduced lung viral RNA load to levels statistically significantly lower than those observed with Hu-S2P6 at 20 mg/kg, suggesting a beneficial effect of S2P6 effector functions in vivo. Based on the comparable S2P6 neutralization potencies toward SARS-CoV-2 VOC observed in vitro (Fig. 1F), we assessed the protective efficacy of S2P6 in hamsters challenged with SARS-CoV-2 B.1.351 (beta VOC). Prophylactic administration of Hu-S2P6 at 20 mg/kg reduced replicating viral titers in the lungs (but not RNA copy numbers) by ~1.5 order of magnitude relative to the control group. Although this difference was not statistically significant, the observed efficacy against this VOC is in line with the strict conservation of the stem helix epitope in all VOC identified to date (Fig. 4F).

Collectively, these findings demonstrate that Abs targeting a highly conserved epitope in the S fusion machinery can trigger Fc-mediated ADCC and ADCP in vitro and that their in vivo antiviral activity may rely on both neutralization and effector functions.

### *Natural infection or vaccination predominantly elicit stem helix-directed Abs of narrow specificities*

To understand how frequently stem helix-specific Abs are elicited, we analyzed plasma samples from pre-pandemic, COVID-19 convalescent, and COVID-19 vaccinated individuals to determine IgG binding titers to the SARS-CoV-2/SARS-CoV (SARS-CoV/-2), OC43, MERS-CoV, HKU1, HKU4 and HKU5 stem helix peptides (Fig. 5A and fig. S7). In pre-pandemic samples, we only observed binding to the HKU1 stem helix peptide, probably reflecting prior infection with this virus in the cohort. Stem helix-specific Abs were found at low frequencies in individuals previously infected with SARS-CoV-2 or those who had received two doses of mRNA vaccines (Fig. 5A). The highest frequencies of stem helix-specific serum Abs were observed for vaccinated individuals who were previously infected (ranging from 22% to 78% against the different stem helix peptides). Overall, these data show that plasma Ab responses to the stem helix are elicited upon SARS-CoV-2 infection or vaccination although they are relatively rare. Evaluation of the prevalence of mAbs targeting other fusion machinery epitopes will reveal if this trend holds true for the overall $S_2$ subunit or if it is a specificity of the cryptic stem helix epitope.

Next, we investigated the frequencies of stem helix-specific memory B cells among 21 convalescent and 17 vaccinated individuals using a clonal analysis based on in vitro polyclonal stimulation (*40*) (Fig. 5, B to G, and figs. S7 to S9). In both cohorts, we observed frequencies of culture positive for stem helix-specific IgGs ranging from 0-2.5%, except for one individual (infected and vaccinated with a single dose of mRNA vaccine) for which 99.6% of culture supernatants were binding to the SARS-CoV/-2 stem helix (fig. S9). Most SARS-CoV-2 stem helix-specific memory B cells were cross-reactive with OC43, consistent with the high sequence identity of the stem helices of these two viruses (Fig. 1H). Abs specific for the HKU1 S stem helix were found but they were not cross-reactive with other β-coronaviruses except for one convalescent and one vaccinated individuals (Fig. 5, D and G). This analysis revealed a single example of cross-reactivity to all stem helix β-coronavirus peptides tested (Fig. 5E), whereas most other Abs show limited cross-reactivity among β-coronaviruses.

### *Broadly reactive β-coronavirus Abs enhance their binding affinity and cross-reactivity through somatic mutations*

To define the ontogeny of the broadly reactive β-coronavirus mAbs presented here, we generated a panel of germline variants of S2P6, P34D10, P34E3 and P34G12. Two out of seven S2P6 heavy chain residues that are mutated relative to germline contribute to epitope recognition (Q32 and H57) whereas none of the 5 light chain mutated residues participate in S binding (Fig. 2, C and D). To address the role of VH and VK somatic mutations we generated a panel of S2P6 germline variants for the heavy or the light chain, or both variable regions (VH and VK). The fully germline reverted S2P6 (unmutated common ancestor - UCA) bound to OC43 and MERS-CoV stem helix peptides (with approximately 1 order of magnitude higher EC50 compared to the mature mAb) but did not bind to SARS-CoV/-2 or HKU1 stem helix peptides or spike trimers (Fig. 5H and fig. S10, A to C). Somatic mutations in VH were sufficient for high avidity binding to SARS-CoV/-2, whereas both VH and VK mutations were required

for optimal binding to HKU1. The presence of residue G103 in CDRH3 was essential for binding to all β-coronaviruses (fig. S10, A to C). These findings indicate that the S2P6 mAb likely arose in response to OC43 infection, and its specificity was broadened toward SARS-CoV-2 and HKU1 through somatic mutations selected upon natural infection with one or both of these β-coronaviruses. In contrast, analysis of UCA binding of the clonally related P34D10, P34G12 and P34E3 mAbs suggest they were likely primed by HKU1 infection, rather than OC43, and acquired cross-reactivity toward the other human β-coronaviruses primarily through somatic mutations in VH (Fig. 5I and fig. S10, D to F). Collectively, these findings demonstrate that broadly reactive β-coronavirus Abs may result from priming of virus-specific B cells gaining affinity and cross-reactivity through somatic mutations in response to heterotypic coronavirus exposures.

## Discussion

The coronavirus $S_2$ subunit (fusion machinery) contains several conserved antigenic sites, including the fusion peptide and the heptad-repeat 2 regions (*41*–*46*). The recent identification of 4 cross-reactive mAbs targeting the stem helix in the $S_2$ subunit highlighted the importance of this epitope, which is conserved among β-coronavirus but not α-coronavirus S glycoproteins (*14*–*16*, *47*), although none of these mAbs inhibit members of all three β-coronavirus subgenera. Here, we identified five stem helix-specific human mAbs cross-reacting with human and animal β-coronaviruses and showed that S2P6 broadly neutralized all sarbecoviruses, merbecoviruses and embecoviruses pseudotypes evaluated through inhibition of membrane fusion.

We show that a $S_2$ subunit-directed mAb reduces lung viral burden in hamsters challenged with SARS-CoV-2 with a key contribution of Fc-mediated effector functions, as described for some SARS-CoV-2 RBD-specific mAbs (*34*, *37*) and influenza virus hemagglutinin stem mAbs (*17*, *48*, *49*). While this study showcases the stem helix as a target of pan-β-coronavirus Abs, the existence of other cross-reactive $S_2$ epitopes with neutralizing potential remains a subject of investigation. Stem helix-targeted Abs are elicited upon natural infection by endemic (OC43 or HKU1) or pandemic (SARS-CoV-2) coronaviruses as well as by COVID-19 mRNA vaccines. Similarly to the subdominance of Abs recognizing the conserved hemagglutinin stem region of influenza A viruses (*48*, *50*), stem helix-specific Abs are present at low titers in convalescent or vaccinees plasma and at low frequencies in their memory B cell repertoires, possibly as a result of limited epitope exposure. These findings along with the moderate neutralization potency of these mAbs indicate that eliciting high enough titers of stem helix-targeted mAbs through vaccination will be a key challenge to overcome to develop pan-β-coronavirus vaccines. We propose that harnessing recent advances in computational protein design, such as epitope-focused vaccine design approaches as described for the respiratory syncytial virus fusion protein (*51*–*53*) and multivalent display as described for influenza virus (*54*–*59*) to target the S stem helix or the fusion peptide regions might enable elicitation of broad β-coronavirus immunity.

## REFERENCES AND NOTES


1. M. A. Tortorici, D. Veesler, Structural insights into coronavirus entry. *Adv. Virus Res.* **105**, 93–116 (2019). doi:10.1016/bs.aivir.2019.08.002 Medline
2. A. C. Walls, Y. J. Park, M. A. Tortorici, A. Wall, A. T. McGuire, D. Veesler, Structure, Function, and Antigenicity of the SARS-CoV-2 Spike Glycoprotein. *Cell* **181**, 281–292.e6 (2020). doi:10.1016/j.cell.2020.02.058 Medline
3. D. Wrapp, N. Wang, K. S. Corbett, J. A. Goldsmith, C. L. Hsieh, O. Abiona, B. S. Graham, J. S. McLellan, Cryo-EM structure of the 2019-nCoV spike in the prefusion conformation. *Science* **367**, 1260–1263 (2020). doi:10.1126/science.abb2507 Medline
4. E. C. Thomson, L. E. Rosen, J. G. Shepherd, R. Spreafico, A. da Silva Filipe, J. A. Wojcechowskyj, C. Davis, L. Piccoli, D. J. Pascall, J. Dillen, S. Lytras, N. Czudnochowski, R. Shah, M. Meury, N. Jesudason, A. De Marco, K. Li, J. Bassi, A. O'Toole, D. Pinto, R. M. Colquhoun, K. Culap, B. Jackson, F. Zatta, A. Rambaut, S. Jaconi, V. B. Sreenu, J. Nix, I. Zhang, R. F. Jarrett, W. G. Glass, M. Beltramello, K. Nomikou, M. Pizzuto, L. Tong, E. Cameroni, T. I. Croll, N. Johnson, J. Di Iulio, A. Wickenhagen, A. Ceschi, A. M. Harbison, D. Mair, P. Ferrari, K. Smollett, F. Sallusto, S. Carmichael, C. Garzoni, J. Nichols, M. Galli, J. Hughes, A. Riva, A. Ho, M. Schiuma, M. G. Semple, P. J. M. Openshaw, E. Fadda, J. K. Baillie, J. D. Chodera, ISARIC4C Investigators, COVID-19 Genomics UK (COG-UK) Consortium, S. J. Rihn, S. J. Lycett, H. W. Virgin, A. Telenti, D. Corti, D. L. Robertson, G. Snell, Circulating SARS-CoV-2 spike N439K variants maintain fitness while evading antibody-mediated immunity. *Cell* **184**, 1171–1187.e20 (2021). doi:10.1016/j.cell.2021.01.037 Medline
5. M. McCallum, A. De Marco, F. A. Lempp, M. A. Tortorici, D. Pinto, A. C. Walls, M. Beltramello, A. Chen, Z. Liu, F. Zatta, S. Zepeda, J. di Iulio, J. E. Bowen, M. Montiel-Ruiz, J. Zhou, L. E. Rosen, S. Bianchi, B. Guarino, C. S. Fregni, R. Abdelnabi, S.-Y. C. Foo, P. W. Rothlauf, L.-M. Bloyet, F. Benigni, E. Cameroni, J. Neyts, A. Riva, G. Snell, A. Telenti, S. P. J. Whelan, H. W. Virgin, D. Corti, M. S. Pizzuto, D. Veesler, N-terminal domain antigenic mapping reveals a site of vulnerability for SARS-CoV-2. *Cell* **184**, 2332–2347.e16 (2021). doi:10.1016/j.cell.2021.03.028 Medline
6. K. R. McCarthy, L. J. Rennick, S. Nambulli, L. R. Robinson-McCarthy, W. G. Bain, G. Haidar, W. P. Duprex, Recurrent deletions in the SARS-CoV-2 spike glycoprotein drive antibody escape. *Science* **371**, 1139–1142 (2021). doi:10.1126/science.abf6950 Medline
7. B. Choi, M. C. Choudhary, J. Regan, J. A. Sparks, R. F. Padera, X. Qiu, I. H. Solomon, H. H. Kuo, J. Boucau, K. Bowman, U. D. Adhikari, M. L. Winkler, A. A. Mueller, T. Y. Hsu, M. Desjardins, L. R. Baden, B. T. Chan, B. D. Walker, M. Lichterfeld, M. Brigl, D. S. Kwon, S. Kanjilal, E. T. Richardson, A. H. Jonsson, G. Alter, A. K. Barczak, W. P. Hanage, X. G. Yu, G. D. Gaiha, M. S. Seaman, M. Cernadas, J. Z. Li, Persistence and Evolution of SARS-CoV-2 in an Immunocompromised Host. *N. Engl. J. Med.* **383**, 2291–2293 (2020). doi:10.1056/NEJMc2031364 Medline
8. Z. Liu, L. A. VanBlargan, L.-M. Bloyet, P. W. Rothlauf, R. E. Chen, S. Stumpf, H. Zhao, J. M. Errico, E. S. Theel, M. J. Liebeskind, B. Alford, W. J. Buchser, A. H. Ellebedy, D. H. Fremont, M. S. Diamond, S. P. J. Whelan, Identification of SARS-CoV-2 spike mutations that attenuate monoclonal and serum antibody neutralization. *Cell Host Microbe* **29**, 477–488.e4 (2021). doi:10.1016/j.chom.2021.01.014 Medline
9. Y. Weisblum, F. Schmidt, F. Zhang, J. DaSilva, D. Poston, J. C. C. Lorenzi, F. Muecksch, M. Rutkowska, H.-H. Hoffmann, E. Michailidis, C. Gaebler, M. Agudelo, A. Cho, Z. Wang, A. Gazumyan, M. Cipolla, L. Luchsinger, C. D. Hillyer, M. Caskey, D. F. Robbiani, C. M. Rice, M. C. Nussenzweig, T. Hatziioannou, P. D. Bieniasz, Escape from neutralizing antibodies by SARS-CoV-2 spike protein variants. *eLife* **9**, e61312 (2020). doi:10.7554/eLife.61312 Medline
10. M. McCallum, J. Bassi, A. De Marco, A. Chen, A. C. Walls, J. Di Iulio, M. A. Tortorici, M.-J. Navarro, C. Silacci-Fregni, C. Saliba, K. R. Sprouse, M. Agostini, D. Pinto, K.

Culap, S. Bianchi, S. Jaconi, E. Cameroni, J. E. Bowen, S. W. Tilles, M. S. Pizzuto, S. B. Guastalla, G. Bona, A. F. Pellanda, C. Garzoni, W. C. Van Voorhis, L. E. Rosen, G. Snell, A. Telenti, H. W. Virgin, L. Piccoli, D. Corti, D. Veesler, SARS-CoV-2 immune evasion by the B.1.427/B.1.429 variant of concern. *Science* 10.1126/science.abi7994 (2021). doi:10.1126/science.abi7994 Medline

11. A. C. Walls, X. Xiong, Y. J. Park, M. A. Tortorici, J. Snijder, J. Quispe, E. Cameroni, R. Gopal, M. Dai, A. Lanzavecchia, M. Zambon, F. A. Rey, D. Corti, D. Veesler, Unexpected Receptor Functional Mimicry Elucidates Activation of Coronavirus Fusion. *Cell* **176**, 1026–1039.e15 (2019). doi:10.1016/j.cell.2018.12.028 Medline
12. Y. Watanabe, J. D. Allen, D. Wrapp, J. S. McLellan, M. Crispin, Site-specific glycan analysis of the SARS-CoV-2 spike. *Science* **369**, 330–333 (2020). doi:10.1126/science.abb9983 Medline
13. Y. Yang, C. Liu, L. Du, S. Jiang, Z. Shi, R. S. Baric, F. Li, Two Mutations Were Critical for Bat-to-Human Transmission of Middle East Respiratory Syndrome Coronavirus. *J. Virol.* **89**, 9119–9123 (2015). doi:10.1128/JVI.01279-15 Medline
14. M. M. Sauer, M. A. Tortorici, Y.-J. Park, A. C. Walls, L. Homad, O. Acton, J. Bowen, C. Wang, X. Xiong, W. de van der Schueren, J. Quispe, B. G. Hoffstrom, B.-J. Bosch, A. T. McGuire, D. Veesler, Structural basis for broad coronavirus neutralization. bioRxiv 2020.12.29.424482 [Preprint] (2021). https://doi.org/10.1101/2020.12.29.424482.
15. C. Wang, R. van Haperen, J. Gutiérrez-Álvarez, W. Li, N. M. A. Okba, I. Albulescu, I. Widjaja, B. van Dieren, R. Fernandez-Delgado, I. Sola, D. L. Hurdiss, O. Daramola, F. Grosveld, F. J. M. van Kuppeveld, B. L. Haagmans, L. Enjuanes, D. Drabek, B.-J. Bosch, A conserved immunogenic and vulnerable site on the coronavirus spike protein delineated by cross-reactive monoclonal antibodies. *Nat. Commun.* **12**, 1715 (2021). doi:10.1038/s41467-021-21968-w Medline
16. G. Song, W.-t. He, S. Callaghan, F. Anzanello, D. Huang, J. Ricketts, J. L. Torres, N. Beutler, L. Peng, S. Vargas, J. Cassell, M. Parren, L. Yang, C. Ignacio, D. M. Smith, J. E. Voss, D. Nemazee, A. B. Ward, T. Rogers, D. R. Burton, R. Andrabi, Cross-reactive serum and memory B-cell responses to spike protein in SARS-CoV-2 and endemic coronavirus infection. *Nat. Commun.* **12**, 2938 (2021). doi:10.1038/s41467-021-23074-3 Medline
17. N. L. Kallewaard, D. Corti, P. J. Collins, U. Neu, J. M. McAuliffe, E. Benjamin, L. Wachter-Rosati, F. J. Palmer-Hill, A. Q. Yuan, P. A. Walker, M. K. Vorlaender, S. Bianchi, B. Guarino, A. De Marco, F. Vanzetta, G. Agatic, M. Foglierini, D. Pinna, B. Fernandez-Rodriguez, A. Fruehwirth, C. Silacci, R. W. Ogrodowicz, S. R. Martin, F. Sallusto, J. A. Suzich, A. Lanzavecchia, Q. Zhu, S. J. Gamblin, J. J. Skehel, Structure and Function Analysis of an Antibody Recognizing All Influenza A Subtypes. *Cell* **166**, 596–608 (2016). doi:10.1016/j.cell.2016.05.073 Medline
18. B. F. Haynes, D. R. Burton, J. R. Mascola, Multiple roles for HIV broadly neutralizing antibodies. *Sci. Transl. Med.* **11**, eaaz2686 (2019). doi:10.1126/scitranslmed.aaz2686 Medline
19. Z. Wang, F. Schmidt, Y. Weisblum, F. Muecksch, C. O. Barnes, S. Finkin, D. Schaefer-Babajew, M. Cipolla, C. Gaebler, J. A. Lieberman, T. Y. Oliveira, Z. Yang, M. E. Abernathy, K. E. Huey-Tubman, A. Hurley, M. Turroja, K. A. West, K. Gordon, K. G. Millard, V. Ramos, J. Da Silva, J. Xu, R. A. Colbert, R. Patel, J. Dizon, C. Unson-O'Brien, I. Shimeliovich, A. Gazumyan, M. Caskey, P. J. Bjorkman, R. Casellas, T. Hatziioannou, P. D. Bieniasz, M. C. Nussenzweig, mRNA vaccine-elicited antibodies to SARS-CoV-2 and circulating variants. *Nature* **592**, 616–622 (2021). doi:10.1038/s41586-021-03324-6 Medline
20. L. Piccoli, Y. J. Park, M. A. Tortorici, N. Czudnochowski, A. C. Walls, M. Beltramello, C. Silacci-Fregni, D. Pinto, L. E. Rosen, J. E. Bowen, O. J. Acton, S. Jaconi, B. Guarino, A. Minola, F. Zatta, N. Sprugasci, J. Bassi, A. Peter, A. De Marco, J. C. Nix, F. Mele, S. Jovic, B. F. Rodriguez, S. V. Gupta, F. Jin, G. Piumatti, G. Lo Presti, A. F. Pellanda, M. Biggiogero, M. Tarkowski, M. S. Pizzuto, E. Cameroni, C. Havenar-Daughton, M. Smithey, D. Hong, V. Lepori, E. Albanese, A. Ceschi, E. Bernasconi, L. Elzi, P. Ferrari, C. Garzoni, A. Riva, G. Snell, F. Sallusto, K. Fink, H. W. Virgin, A. Lanzavecchia, D. Corti, D. Veesler, Mapping Neutralizing and Immunodominant Sites on the SARS-CoV-2 Spike Receptor-Binding Domain by Structure-Guided High-Resolution Serology. *Cell* **183**, 1024–1042.e21 (2020). doi:10.1016/j.cell.2020.09.037 Medline
21. M. A. Tortorici, M. Beltramello, F. A. Lempp, D. Pinto, H. V. Dang, L. E. Rosen, M. McCallum, J. Bowen, A. Minola, S. Jaconi, F. Zatta, A. De Marco, B. Guarino, S. Bianchi, E. J. Lauron, H. Tucker, J. Zhou, A. Peter, C. Havenar-Daughton, J. A. Wojcechowskyj, J. B. Case, R. E. Chen, H. Kaiser, M. Montiel-Ruiz, M. Meury, N. Czudnochowski, R. Spreafico, J. Dillen, C. Ng, N. Sprugasci, K. Culap, F. Benigni, R. Abdelnabi, S. C. Foo, M. A. Schmid, E. Cameroni, A. Riva, A. Gabrieli, M. Galli, M. S. Pizzuto, J. Neyts, M. S. Diamond, H. W. Virgin, G. Snell, D. Corti, K. Fink, D. Veesler, Ultrapotent human antibodies protect against SARS-CoV-2 challenge via multiple mechanisms. *Science* **370**, 950–957 (2020). doi:10.1126/science.abe3354 Medline
22. M. Hoffmann, K. Mösbauer, H. Hofmann-Winkler, A. Kaul, H. Kleine-Weber, N. Krüger, N. C. Gassen, M. A. Müller, C. Drosten, S. Pöhlmann, Chloroquine does not inhibit infection of human lung cells with SARS-CoV-2. *Nature* **585**, 588–590 (2020). doi:10.1038/s41586-020-2575-3 Medline
23. M. Hoffmann, H. Kleine-Weber, S. Schroeder, N. Krüger, T. Herrler, S. Erichsen, T. S. Schiergens, G. Herrler, N. H. Wu, A. Nitsche, M. A. Müller, C. Drosten, S. Pöhlmann, SARS-CoV-2 Cell Entry Depends on ACE2 and TMPRSS2 and Is Blocked by a Clinically Proven Protease Inhibitor. *Cell* **181**, 271–280.e8 (2020). doi:10.1016/j.cell.2020.02.052 Medline
24. M. Hoffmann, H. Kleine-Weber, S. Pöhlmann, A Multibasic Cleavage Site in the Spike Protein of SARS-CoV-2 Is Essential for Infection of Human Lung Cells. *Mol. Cell* **78**, 779–784.e5 (2020). doi:10.1016/j.molcel.2020.04.022 Medline
25. Y. Kaname, H. Tani, C. Kataoka, M. Shiokawa, S. Taguwa, T. Abe, K. Moriishi, T. Kinoshita, Y. Matsuura, Acquisition of complement resistance through incorporation of CD55/decay-accelerating factor into viral particles bearing baculovirus GP64. *J. Virol.* **84**, 3210–3219 (2010). doi:10.1128/JVI.02519-09 Medline
26. J. B. Case, P. W. Rothlauf, R. E. Chen, Z. Liu, H. Zhao, A. S. Kim, L. M. Bloyet, Q. Zeng, S. Tahan, L. Droit, M. X. G. Ilagan, M. A. Tartell, G. Amarasinghe, J. P. Henderson, S. Miersch, M. Ustav, S. Sidhu, H. W. Virgin, D. Wang, S. Ding, D. Corti, E. S. Theel, D. H. Fremont, M. S. Diamond, S. P. J. Whelan, Neutralizing Antibody and Soluble ACE2 Inhibition of a Replication-Competent VSV-SARS-CoV-2 and a Clinical Isolate of SARS-CoV-2. *Cell Host Microbe* **28**, 475–485.e5 (2020). doi:10.1016/j.chom.2020.06.021 Medline
27. M. Gui, W. Song, H. Zhou, J. Xu, S. Chen, Y. Xiang, X. Wang, Cryo-electron microscopy structures of the SARS-CoV spike glycoprotein reveal a prerequisite conformational state for receptor binding. *Cell Res.* **27**, 119–129 (2017). doi:10.1038/cr.2016.152 Medline
28. R. N. Kirchdoerfer, N. Wang, J. Pallesen, D. Wrapp, H. L. Turner, C. A. Cottrell, K. S. Corbett, B. S. Graham, J. S. McLellan, A. B. Ward, Stabilized coronavirus spikes are resistant to conformational changes induced by receptor recognition or proteolysis. *Sci. Rep.* **8**, 15701 (2018). doi:10.1038/s41598-018-34171-7 Medline
29. Y. Yuan, D. Cao, Y. Zhang, J. Ma, J. Qi, Q. Wang, G. Lu, Y. Wu, J. Yan, Y. Shi, X. Zhang, G. F. Gao, Cryo-EM structures of MERS-CoV and SARS-CoV spike glycoproteins reveal the dynamic receptor binding domains. *Nat. Commun.* **8**, 15092 (2017). doi:10.1038/ncomms15092 Medline
30. A. C. Walls, M. A. Tortorici, J. Snijder, X. Xiong, B. J. Bosch, F. A. Rey, D. Veesler, Tectonic conformational changes of a coronavirus spike glycoprotein promote membrane fusion. *Proc. Natl. Acad. Sci. U.S.A.* **114**, 11157–11162 (2017). doi:10.1073/pnas.1708727114 Medline
31. Y. Cai, J. Zhang, T. Xiao, H. Peng, S. M. Sterling, R. M. Walsh Jr., S. Rawson, S. Rits-Volloch, B. Chen, Distinct conformational states of SARS-CoV-2 spike protein. *Science* **369**, 1586–1592 (2020). doi:10.1126/science.abd4251 Medline
32. X. Fan, D. Cao, L. Kong, X. Zhang, Cryo-EM analysis of the post-fusion structure of the SARS-CoV spike glycoprotein. *Nat. Commun.* **11**, 3618 (2020). doi:10.1038/s41467-020-17371-6 Medline
33. F. A. Lempp, L. Soriaga, M. Montiel-Ruiz, F. Benigni, J. Noack, Y.-J. Park, S. Bianchi, A. C. Walls, J. E. Bowen, J. Zhou, H. Kaiser, M. Agostini, M. Meury, E. Dellota Jr., S. Jaconi, E. Cameroni, H. W. Virgin, A. Lanzavecchia, D. Veesler, L. Purcell, A. Telenti, D. Corti, Membrane lectins enhance SARS-CoV-2 infection and influence the neutralizing activity of different classes of antibodies. bioRxiv 2021.04.03.438258 [Preprint] (2021). https://doi.org/10.1101/2021.04.03.438258.
34. A. Schäfer, F. Muecksch, J. C. C. Lorenzi, S. R. Leist, M. Cipolla, S. Bournazos, F. Schmidt, R. M. Maison, A. Gazumyan, D. R. Martinez, R. S. Baric, D. F. Robbiani, T. Hatziioannou, J. V. Ravetch, P. D. Bieniasz, R. A. Bowen, M. C. Nussenzweig, T. P. Sheahan, Antibody potency, effector function, and combinations in protection and therapy for SARS-CoV-2 infection in vivo. *J. Exp. Med.* **218**, e20201993 (2021). doi:10.1084/jem.20201993 Medline

35. S. Bournazos, T. T. Wang, J. V. Ravetch, The Role and Function of Fcγ Receptors on Myeloid Cells. *Microbiol. Spectr.* **4**, 4.6.20 (2016). doi:10.1128/microbiolspec.MCHD-0045-2016 Medline
36. S. Bournazos, D. Corti, H. W. Virgin, J. V. Ravetch, Fc-optimized antibodies elicit CD8 immunity to viral respiratory infection. *Nature* **588**, 485–490 (2020). doi:10.1038/s41586-020-2838-z Medline
37. E. S. Winkler, P. Gilchuk, J. Yu, A. L. Bailey, R. E. Chen, Z. Chong, S. J. Zost, H. Jang, Y. Huang, J. D. Allen, J. B. Case, R. E. Sutton, R. H. Carnahan, T. L. Darling, A. C. M. Boon, M. Mack, R. D. Head, T. M. Ross, J. E. Crowe Jr., M. S. Diamond, Human neutralizing antibodies against SARS-CoV-2 require intact Fc effector functions for optimal therapeutic protection. *Cell* **184**, 1804–1820.E16 (2021). doi:10.1016/j.cell.2021.02.026 Medline
38. D. Pinto, Y. J. Park, M. Beltramello, A. C. Walls, M. A. Tortorici, S. Bianchi, S. Jaconi, K. Culap, F. Zatta, A. De Marco, A. Peter, B. Guarino, R. Spreafico, E. Cameroni, J. B. Case, R. E. Chen, C. Havenar-Daughton, G. Snell, A. Telenti, H. W. Virgin, A. Lanzavecchia, M. S. Diamond, K. Fink, D. Veesler, D. Corti, Cross-neutralization of SARS-CoV-2 by a human monoclonal SARS-CoV antibody. *Nature* **583**, 290–295 (2020). doi:10.1038/s41586-020-2349-y Medline
39. R. Boudewijns, H. J. Thibaut, S. J. F. Kaptein, R. Li, V. Vergote, L. Seldeslachts, J. Van Weyenbergh, C. De Keyzer, L. Bervoets, S. Sharma, L. Liesenborghs, J. Ma, S. Jansen, D. Van Looveren, T. Vercruysse, X. Wang, D. Jochmans, E. Martens, K. Roose, D. De Vlieger, B. Schepens, T. Van Buyten, S. Jacobs, Y. Liu, J. Martí-Carreras, B. Vanmechelen, T. Wawina-Bokalanga, L. Delang, J. Rocha-Pereira, L. Coelmont, W. Chiu, P. Leyssen, E. Heylen, D. Schols, L. Wang, L. Close, J. Matthijnssens, M. Van Ranst, V. Compernolle, G. Schramm, K. Van Laere, X. Saelens, N. Callewaert, G. Opdenakker, P. Maes, B. Weynand, C. Cawthorne, G. Vande Velde, Z. Wang, J. Neyts, K. Dallmeier, STAT2 signaling restricts viral dissemination but drives severe pneumonia in SARS-CoV-2 infected hamsters. *Nat. Commun.* **11**, 5838 (2020). doi:10.1038/s41467-020-19684-y Medline
40. D. Pinna, D. Corti, D. Jarrossay, F. Sallusto, A. Lanzavecchia, Clonal dissection of the human memory B-cell repertoire following infection and vaccination. *Eur. J. Immunol.* **39**, 1260–1270 (2009). doi:10.1002/eji.200839129 Medline
41. C. Daniel, R. Anderson, M. J. Buchmeier, J. O. Fleming, W. J. Spaan, H. Wege, P. J. Talbot, Identification of an immunodominant linear neutralization domain on the S2 portion of the murine coronavirus spike glycoprotein and evidence that it forms part of complex tridimensional structure. *J. Virol.* **67**, 1185–1194 (1993). doi:10.1128/jvi.67.3.1185-1194.1993 Medline
42. H. Zhang, G. Wang, J. Li, Y. Nie, X. Shi, G. Lian, W. Wang, X. Yin, Y. Zhao, X. Qu, M. Ding, H. Deng, Identification of an antigenic determinant on the S2 domain of the severe acute respiratory syndrome coronavirus spike glycoprotein capable of inducing neutralizing antibodies. *J. Virol.* **78**, 6938–6945 (2004). doi:10.1128/JVI.78.13.6938-6945.2004 Medline
43. C. M. Poh, G. Carissimo, B. Wang, S. N. Amrun, C. Y. Lee, R. S. Chee, S. W. Fong, N. K. Yeo, W. H. Lee, A. Torres-Ruesta, Y. S. Leo, M. I. Chen, S. Y. Tan, L. Y. A. Chai, S. Kalimuddin, S. S. G. Kheng, S. Y. Thien, B. E. Young, D. C. Lye, B. J. Hanson, C. I. Wang, L. Renia, L. F. P. Ng, Two linear epitopes on the SARS-CoV-2 spike protein that elicit neutralising antibodies in COVID-19 patients. *Nat. Commun.* **11**, 2806 (2020). doi:10.1038/s41467-020-16638-2 Medline
44. H. A. Elshabrawy, M. M. Coughlin, S. C. Baker, B. S. Prabhakar, Human monoclonal antibodies against highly conserved HR1 and HR2 domains of the SARS-CoV spike protein are more broadly neutralizing. *PLOS ONE* **7**, e50366 (2012). doi:10.1371/journal.pone.0050366 Medline
45. Z. Zheng, V. M. Monteil, S. Maurer-Stroh, C. W. Yew, C. Leong, N. K. Mohd-Ismail, S. Cheyyatraivendran Arularasu, V. T. K. Chow, R. T. P. Lin, A. Mirazimi, W. Hong, Y. J. Tan, Monoclonal antibodies for the S2 subunit of spike of SARS-CoV-1 cross-react with the newly-emerged SARS-CoV-2. *Euro Surveill.* **25**, 2000291 (2020). doi:10.2807/1560-7917.ES.2020.25.28.2000291 Medline
46. A. C. Walls, M. A. Tortorici, B. J. Bosch, B. Frenz, P. J. M. Rottier, F. DiMaio, F. A. Rey, D. Veesler, Cryo-electron microscopy structure of a coronavirus spike glycoprotein trimer. *Nature* **531**, 114–117 (2016). doi:10.1038/nature16988 Medline
47. P. Zhou, M. Yuan, G. Song, N. Beutler, N. Shaabani, D. Huang, W.-t. He, X. Zhu, S. Callaghan, P. Yong, F. Anzanello, L. Peng, J. Ricketts, M. Parren, E. Garcia, S. A. Rawlings, D. M. Smith, D. Nemazee, J. R. Teijaro, T. F. Rogers, I. A. Wilson, D. R. Burton, R. Andrabi, A protective broadly cross-reactive human antibody defines a conserved site of vulnerability on beta-coronavirus spikes. bioRxiv 2021.03.30.437769 [Preprint] (2021). https://doi.org/10.1101/2021.03.30.437769.
48. D. Corti, J. Voss, S. J. Gamblin, G. Codoni, A. Macagno, D. Jarrossay, S. G. Vachieri, D. Pinna, A. Minola, F. Vanzetta, C. Silacci, B. M. Fernandez-Rodriguez, G. Agatic, S. Bianchi, I. Giacchetto-Sasselli, L. Calder, F. Sallusto, P. Collins, L. F. Haire, N. Temperton, J. P. Langedijk, J. J. Skehel, A. Lanzavecchia, A neutralizing antibody selected from plasma cells that binds to group 1 and group 2 influenza A hemagglutinins. *Science* **333**, 850–856 (2011). doi:10.1126/science.1205669 Medline
49. D. J. DiLillo, G. S. Tan, P. Palese, J. V. Ravetch, Broadly neutralizing hemagglutinin stalk-specific antibodies require FcγR interactions for protection against influenza virus in vivo. *Nat. Med.* **20**, 143–151 (2014). doi:10.1038/nm.3443 Medline
50. D. Corti, A. L. Suguitan Jr., D. Pinna, C. Silacci, B. M. Fernandez-Rodriguez, F. Vanzetta, C. Santos, C. J. Luke, F. J. Torres-Velez, N. J. Temperton, R. A. Weiss, F. Sallusto, K. Subbarao, A. Lanzavecchia, Heterosubtypic neutralizing antibodies are produced by individuals immunized with a seasonal influenza vaccine. *J. Clin. Invest.* **120**, 1663–1673 (2010). doi:10.1172/JCI41902 Medline
51. F. Sesterhenn, C. Yang, J. Bonet, J. T. Cramer, X. Wen, Y. Wang, C. I. Chiang, L. A. Abriata, I. Kucharska, G. Castoro, S. S. Vollers, M. Galloux, E. Dheilly, S. Rosset, P. Corthésy, S. Georgeon, M. Villard, C. A. Richard, D. Descamps, T. Delgado, E. Oricchio, M. A. Rameix-Welti, V. Más, S. Ervin, J. F. Eléouët, S. Riffault, J. T. Bates, J. P. Julien, Y. Li, T. Jardetzky, T. Krey, B. E. Correia, De novo protein design enables the precise induction of RSV-neutralizing antibodies. *Science* **368**, eaay5051 (2020). doi:10.1126/science.aay5051 Medline
52. M. L. Azoitei, B. E. Correia, Y. E. Ban, C. Carrico, O. Kalyuzhniy, L. Chen, A. Schroeter, P. S. Huang, J. S. McLellan, P. D. Kwong, D. Baker, R. K. Strong, W. R. Schief, Computation-guided backbone grafting of a discontinuous motif onto a protein scaffold. *Science* **334**, 373–376 (2011). doi:10.1126/science.1209368 Medline
53. B. E. Correia, J. T. Bates, R. J. Loomis, G. Baneyx, C. Carrico, J. G. Jardine, P. Rupert, C. Correnti, O. Kalyuzhniy, V. Vittal, M. J. Connell, E. Stevens, A. Schroeter, M. Chen, S. Macpherson, A. M. Serra, Y. Adachi, M. A. Holmes, Y. Li, R. E. Klevit, B. S. Graham, R. T. Wyatt, D. Baker, R. K. Strong, J. E. Crowe Jr., P. R. Johnson, W. R. Schief, Proof of principle for epitope-focused vaccine design. *Nature* **507**, 201–206 (2014). doi:10.1038/nature12966 Medline
54. A. C. Walls, B. Fiala, A. Schäfer, S. Wrenn, M. N. Pham, M. Murphy, L. V. Tse, L. Shehata, M. A. O'Connor, C. Chen, M. J. Navarro, M. C. Miranda, D. Pettie, R. Ravichandran, J. C. Kraft, C. Ogohara, A. Palser, S. Chalk, E. C. Lee, K. Guerriero, E. Kepl, C. M. Chow, C. Sydeman, E. A. Hodge, B. Brown, J. T. Fuller, K. H. Dinnon 3rd, L. E. Gralinski, S. R. Leist, K. L. Gully, T. B. Lewis, M. Guttman, H. Y. Chu, K. K. Lee, D. H. Fuller, R. S. Baric, P. Kellam, L. Carter, M. Pepper, T. P. Sheahan, D. Veesler, N. P. King, Elicitation of Potent Neutralizing Antibody Responses by Designed Protein Nanoparticle Vaccines for SARS-CoV-2. *Cell* **183**, 1367–1382.e17 (2020). doi:10.1016/j.cell.2020.10.043 Medline
55. A. C. Walls, M. C. Miranda, M. N. Pham, A. Schäfer, A. Greaney, P. S. Arunachalam, M.-J. Navarro, M. A. Tortorici, K. Rogers, M. A. O'Connor, L. Shireff, D. E. Ferrell, N. Brunette, E. Kepl, J. Bowen, S. K. Zepeda, T. Starr, C.-L. Hsieh, B. Fiala, S. Wrenn, D. Pettie, C. Sydeman, M. Johnson, A. Blackstone, R. Ravichandran, C. Ogohara, L. Carter, S. W. Tilles, R. Rappuoli, D. T. O'Hagan, R. Van Der Most, W. C. Van Voorhis, J. S. McLellan, H. Kleanthous, T. P. Sheahan, D. H. Fuller, F. Villinger, J. Bloom, B. Pulendran, R. Baric, N. King, D. Veesler, Elicitation of broadly protective sarbecovirus immunity by receptor-binding domain nanoparticle vaccines. bioRxiv 2021.03.15.435528 [Preprint] (2021). https://doi.org/10.1101/2021.03.15.435528.
56. S. Boyoglu-Barnum, D. Ellis, R. A. Gillespie, G. B. Hutchinson, Y.-J. Park, S. M. Moin, O. J. Acton, R. Ravichandran, M. Murphy, D. Pettie, N. Matheson, L. Carter, A. Creanga, M. J. Watson, S. Kephart, S. Ataca, J. R. Vaile, G. Ueda, M. C. Crank, L. Stewart, K. K. Lee, M. Guttman, D. Baker, J. R. Mascola, D. Veesler, B. S. Graham, N. P. King, M. Kanekiyo, Quadrivalent influenza nanoparticle vaccines induce broad protection. *Nature* **592**, 623–628 (2021). doi:10.1038/s41586-021-03365-x Medline
57. M. Kanekiyo, C. J. Wei, H. M. Yassine, P. M. McTamney, J. C. Boyington, J. R. Whittle, S. S. Rao, W. P. Kong, L. Wang, G. J. Nabel, Self-assembling influenza

nanoparticle vaccines elicit broadly neutralizing H1N1 antibodies. *Nature* **499**, 102–106 (2013). doi:10.1038/nature12202 Medline

58. M. Kanekiyo, M. G. Joyce, R. A. Gillespie, J. R. Gallagher, S. F. Andrews, H. M. Yassine, A. K. Wheatley, B. E. Fisher, D. R. Ambrozak, A. Creanga, K. Leung, E. S. Yang, S. Boyoglu-Barnum, I. S. Georgiev, Y. Tsybovsky, M. S. Prabhakaran, H. Andersen, W. P. Kong, U. Baxa, K. L. Zephir, J. E. Ledgerwood, R. A. Koup, P. D. Kwong, A. K. Harris, A. B. McDermott, J. R. Mascola, B. S. Graham, Mosaic nanoparticle display of diverse influenza virus hemagglutinins elicits broad B cell responses. *Nat. Immunol.* **20**, 362–372 (2019). doi:10.1038/s41590-018-0305-x Medline
59. J. Marcandalli, B. Fiala, S. Ols, M. Perotti, W. de van der Schueren, J. Snijder, E. Hodge, M. Benhaim, R. Ravichandran, L. Carter, W. Sheffler, L. Brunner, M. Lawrenz, P. Dubois, A. Lanzavecchia, F. Sallusto, K. K. Lee, D. Veesler, C. E. Correnti, L. J. Stewart, D. Baker, K. Loré, L. Perez, N. P. King, Induction of Potent Neutralizing Antibody Responses by a Designed Protein Nanoparticle Vaccine for Respiratory Syncytial Virus. *Cell* **176**, 1420–1431.e17 (2019). doi:10.1016/j.cell.2019.01.046 Medline
60. J. Tan, B. K. Sack, D. Oyen, I. Zenklusen, L. Piccoli, S. Barbieri, M. Foglierini, C. S. Fregni, J. Marcandalli, S. Jongo, S. Abdulla, L. Perez, G. Corradin, L. Varani, F. Sallusto, B. K. L. Sim, S. L. Hoffman, S. H. I. Kappe, C. Daubenberger, I. A. Wilson, A. Lanzavecchia, A public antibody lineage that potently inhibits malaria infection through dual binding to the circumsporozoite protein. *Nat. Med.* **24**, 401–407 (2018). doi:10.1038/nm.4513 Medline
61. M. A. Tortorici, N. Czudnochowski, T. N. Starr, R. Marzi, A. C. Walls, F. Zatta, J. E. Bowen, S. Jaconi, J. di Iulio, Z. Wang, A. De Marco, S. K. Zepeda, D. Pinto, Z. Liu, M. Beltramello, I. Bartha, M. P. Housley, F. A. Lempp, L. E. Rosen, E. Dellota Jr., H. Kaiser, M. Montiel-Ruiz, J. Zhou, A. Addetia, B. Guarino, K. Culap, N. Sprugasci, C. Saliba, E. Vetti, I. Giacchetto-Sasselli, C. S. Fregni, R. Abdelnabi, S.-Y. C. Foo, C. Havenar-Daughton, M. A. Schmid, F. Benigni, E. Cameroni, J. Neyts, A. Telenti, G. Snell, H. W. Virgin, S. P. J. Whelan, J. D. Bloom, D. Corti, D. Veesler, M. S. Pizzuto, Structural basis for broad sarbecovirus neutralization by a human monoclonal antibody. bioRxiv 2021.04.07.438818 [Preprint] (2021). https://doi.org/10.1101/2021.04.07.438818.
62. M. A. Tortorici, A. C. Walls, Y. Lang, C. Wang, Z. Li, D. Koerhuis, G. J. Boons, B. J. Bosch, F. A. Rey, R. J. de Groot, D. Veesler, Structural basis for human coronavirus attachment to sialic acid receptors. *Nat. Struct. Mol. Biol.* **26**, 481–489 (2019). doi:10.1038/s41594-019-0233-y Medline
63. Y. J. Park, A. C. Walls, Z. Wang, M. M. Sauer, W. Li, M. A. Tortorici, B. J. Bosch, F. DiMaio, D. Veesler, Structures of MERS-CoV spike glycoprotein in complex with sialoside attachment receptors. *Nat. Struct. Mol. Biol.* **26**, 1151–1157 (2019). doi:10.1038/s41594-019-0334-7 Medline
64. T. N. Starr, N. Czudnochowski, F. Zatta, Y.-J. Park, Z. Liu, A. Addetia, D. Pinto, M. Beltramello, P. Hernandez, A. J. Greaney, R. Marzi, W. G. Glass, I. Zhang, A. S. Dingens, J. E. Bowen, J. A. Wojcechowskyj, A. De Marco, L. E. Rosen, J. Zhou, M. Montiel-Ruiz, H. Kaiser, H. Tucker, M. P. Housley, J. di Iulio, G. Lombardo, M. Agostini, N. Sprugasci, K. Culap, S. Jaconi, M. Meury, E. Dellota, E. Cameroni, T. I. Croll, J. C. Nix, C. Havenar-Daughton, A. Telenti, F. A. Lempp, M. S. Pizzuto, J. D. Chodera, C. M. Hebner, S. P. J. Whelan, H. W. Virgin, D. Veesler, D. Corti, J. D. Bloom, G. Snell, Antibodies to the SARS-CoV-2 receptor-binding domain that maximize breadth and resistance to viral escape. bioRxiv 2021.04.06.438709 [Preprint] (2021). https://doi.org/10.1101/2021.04.06.438709.
65. A. M. Bolger, M. Lohse, B. Usadel, Trimmomatic: A flexible trimmer for Illumina sequence data. *Bioinformatics* **30**, 2114–2120 (2014). doi:10.1093/bioinformatics/btu170 Medline
66. H. Li, Aligning sequence reads, clone sequences and assembly contigs with BWA-MEM. arXiv:1303.3997 [q-bio.GN] (2013).
67. A. Wilm, P. P. K. Aw, D. Bertrand, G. H. T. Yeo, S. H. Ong, C. H. Wong, C. C. Khor, R. Petric, M. L. Hibberd, N. Nagarajan, LoFreq: A sequence-quality aware, ultra-sensitive variant caller for uncovering cell-population heterogeneity from high-throughput sequencing datasets. *Nucleic Acids Res.* **40**, 11189–11201 (2012). doi:10.1093/nar/gks918 Medline
68. P. Cingolani, A. Platts, L. Wang, M. Coon, T. Nguyen, L. Wang, S. J. Land, X. Lu, D. M. Ruden, A program for annotating and predicting the effects of single nucleotide polymorphisms, SnpEff. *Fly* **6**, 80–92 (2012). doi:10.4161/fly.19695 Medline
69. B. S. Pedersen, A. R. Quinlan, Mosdepth: Quick coverage calculation for genomes and exomes. *Bioinformatics* **34**, 867–868 (2018). doi:10.1093/bioinformatics/btx699 Medline
70. P. Danecek, J. K. Bonfield, J. Liddle, J. Marshall, V. Ohan, M. O. Pollard, A. Whitwham, T. Keane, S. A. McCarthy, R. M. Davies, H. Li, Twelve years of SAMtools and BCFtools. *Gigascience* **10**, giab008 (2021). doi:10.1093/gigascience/giab008 Medline
71. P. Ewels, M. Magnusson, S. Lundin, M. Käller, MultiQC: Summarize analysis results for multiple tools and samples in a single report. *Bioinformatics* **32**, 3047–3048 (2016). doi:10.1093/bioinformatics/btw354 Medline
72. P. Di Tommaso, M. Chatzou, E. W. Floden, P. P. Barja, E. Palumbo, C. Notredame, Nextflow enables reproducible computational workflows. *Nat. Biotechnol.* **35**, 316–319 (2017). doi:10.1038/nbt.3820 Medline
73. B. Grüning, R. Dale, A. Sjödin, B. A. Chapman, J. Rowe, C. H. Tomkins-Tinch, R. Valieris, J. Köster, Bioconda Team, Bioconda: Sustainable and comprehensive software distribution for the life sciences. *Nat. Methods* **15**, 475–476 (2018). doi:10.1038/s41592-018-0046-7 Medline
74. P. Emsley, B. Lohkamp, W. G. Scott, K. Cowtan, Features and development of *Coot*. *Acta Cryst.* **D66**, 486–501 (2010). doi:10.1107/S0907444910007493 Medline
75. T. I. Croll, *ISOLDE*: A physically realistic environment for model building into low-resolution electron-density maps. *Acta Cryst.* **D74**, 519–530 (2018). doi:10.1107/S2059798318002425 Medline
76. G. N. Murshudov, P. Skubák, A. A. Lebedev, N. S. Pannu, R. A. Steiner, R. A. Nicholls, M. D. Winn, F. Long, A. A. Vagin, *REFMAC5* for the refinement of macromolecular crystal structures. *Acta Cryst.* **D67**, 355–367 (2011). doi:10.1107/S0907444911001314 Medline
77. D. Liebschner, P. V. Afonine, M. L. Baker, G. Bunkóczi, V. B. Chen, T. I. Croll, B. Hintze, L. W. Hung, S. Jain, A. J. McCoy, N. W. Moriarty, R. D. Oeffner, B. K. Poon, M. G. Prisant, R. J. Read, J. S. Richardson, D. C. Richardson, M. D. Sammito, O. V. Sobolev, D. H. Stockwell, T. C. Terwilliger, A. G. Urzhumtsev, L. L. Videau, C. J. Williams, P. D. Adams, Macromolecular structure determination using X-rays, neutrons and electrons: Recent developments in *Phenix*. *Acta Cryst.* **D75**, 861–877 (2019). doi:10.1107/S2059798319011471 Medline
78. C. Suloway, J. Pulokas, D. Fellmann, A. Cheng, F. Guerra, J. Quispe, S. Stagg, C. S. Potter, B. Carragher, Automated molecular microscopy: The new Leginon system. *J. Struct. Biol.* **151**, 41–60 (2005). doi:10.1016/j.jsb.2005.03.010 Medline
79. D. Tegunov, P. Cramer, Real-time cryo-electron microscopy data preprocessing with Warp. *Nat. Methods* **16**, 1146–1152 (2019). doi:10.1038/s41592-019-0580-y Medline
80. A. Punjani, J. L. Rubinstein, D. J. Fleet, M. A. Brubaker, cryoSPARC: Algorithms for rapid unsupervised cryo-EM structure determination. *Nat. Methods* **14**, 290–296 (2017). doi:10.1038/nmeth.4169 Medline
81. J. Zivanov, T. Nakane, S. H. W. Scheres, A Bayesian approach to beam-induced motion correction in cryo-EM single-particle analysis. *IUCrJ* **6**, 5–17 (2019). doi:10.1107/S205225251801463X Medline
82. S. Chen, G. McMullan, A. R. Faruqi, G. N. Murshudov, J. M. Short, S. H. Scheres, R. Henderson, High-resolution noise substitution to measure overfitting and validate resolution in 3D structure determination by single particle electron cryomicroscopy. *Ultramicroscopy* **135**, 24–35 (2013). doi:10.1016/j.ultramic.2013.06.004 Medline
83. E. F. Pettersen, T. D. Goddard, C. C. Huang, G. S. Couch, D. M. Greenblatt, E. C. Meng, T. E. Ferrin, UCSF Chimera—A visualization system for exploratory research and analysis. *J. Comput. Chem.* **25**, 1605–1612 (2004). doi:10.1002/jcc.20084 Medline
84. L. J. Reed, H. Muench, A Simple Method of Estimating Fifty Per Cent Endpoints. *Am. J. Epidemiol.* **27**, 493–497 (1938). doi:10.1093/oxfordjournals.aje.a118408
85. R. Abdelnabi, R. Boudewijns, C. S. Foo, L. Seldeslachts, L. Sanchez-Felipe, X. Zhang, L. Delang, P. Maes, S. J. F. Kaptein, B. Weynand, G. V. Velde, J. Neyts, K. Dallmeier, Comparative infectivity and pathogenesis of emerging SARS-CoV-2 variants in Syrian hamsters. bioRxiv 2021.02.26.433062 [Preprint] (2021). https://doi.org/10.1101/2021.02.26.433062.
86. M. Foglierini, L. Pappas, A. Lanzavecchia, D. Corti, L. Perez, AncesTree: An interactive immunoglobulin lineage tree visualizer. *PLOS Comput. Biol.* **16**, e1007731 (2020). doi:10.1371/journal.pcbi.1007731 Medline

ACKNOWLEDGMENTS

We thank Jay C. Nix for x-ray data collection, and Tristan I. Croll for assistance in refinement of the crystal structure. Thanks to Ann Arvin for her insightful comments. We thank Marcel Meury for help with protein production. We thank Hideki Tani (University of Toyama) for providing the reagents necessary for preparing VSV pseudotyped viruses. The authors would also like to thank Cindy Castado and Normand Blais (GSK Vaccines) for their help in the selection of the genetically divergent sarbecoviruses used in this study. We thank Promega Corporation for kindly providing SARS-CoV2 CHO-K1 cells (genetically engineered to stably express a HaloTag-HiBit-tagged). **Funding:** This study was supported by the National Institute of Allergy and Infectious Diseases (DP1AI158186 and HHSN272201700059C to D.V., and U01 AI151698-01 to WCVV), a Pew Biomedical Scholars Award (D.V.), Investigators in the Pathogenesis of Infectious Disease Awards from the Burroughs Wellcome Fund (D.V.), Fast Grants (D.V.), the Swiss National Science Foundation (P400PB_183942 to M.M.S.), the University of Washington Arnold and Mabel Beckman cryoEM center, the Helmut Horten Foundation (F.S. and the Institute for Research in Biomedicine). The project was also partially funded by the Swiss Kidney Foundation. Use of the Stanford Synchrotron Radiation Lightsource, SLAC National Accelerator Laboratory, is supported by the U.S. Department of Energy, Office of Science, Office of Basic Energy Sciences under Contract No. DE-AC02-76SF00515. The SSRL Structural Molecular Biology Program is supported by the DOE Office of Biological and Environmental Research, and by the National Institutes of Health, National Institute of General Medical Sciences (P30GM133894). The contents of this publication are solely the responsibility of the authors and do not necessarily represent the official views of NIGMS or NIH. **Author contributions:** Experiment design: DP, MM, JSL, MAT, MSP, HWV, MB, DC, DV. Donors' Recruitment and Sample Collection: EC, FB, BG, AC, PF, PEC, OG, SC, CG, AR, LP, MS, DV, CHD. Antibody discovery: DP, MB, JSL, JJ, SJ, NS, KC, EC, DC. AMBRA preparation: CSF, JB, AC. Expression and purification of proteins: ACW, JEB, JJ. Antibody functional experiments: DP, MMS, NC, JSL, MAT, ACW, FL, JN, SB, RM Bioinformatic analysis of virus diversity and variants: JdI, IB, AT. Evaluation of effector functions: BG. SPR Binding Assays: LER. Neutralization assay: DP, MAT, FAL, JN, MPH. Escape mutants selection and sequencing: HK, SI, FAL, JdI. Effects in the hamster model and data analysis: RA, CF, CDK, LB, LC, JN, EV, FB. Cryo-EM Data Collection, Processing, and Model Building, MMS, DV. Crystallization, X-Ray Crystallography Data Collection, Processing, and Model Building: MMS, NC, GS, DV. Serological Assays: DP, RM. Data Analysis: DP, MMS, MB, JSL, JJ, FAL, JN, NC, AMT, ACW, GS, DC, DV. Manuscript Writing: DOP, MMS, JSL, MB, GS, FS, AL, HWV, DC, DV. **Competing interests:** DP, NC, MPH, JN, BG, LER, JdI, HK, SI, SJ, NS, KC, IB, SB, CSF, JB, RM, EV, FB, EC, LP, MSP, MS, DH, AT, FAL, CHD, AL, GS, HWV, MB and DC are employees of Vir Biotechnology and may hold shares in Vir Biotechnology. DC, JSL, FS, AC and AL are currently listed as an inventor on multiple patent applications, which disclose the subject matter described in this manuscript. DV is a consultant for Vir Biotechnology Inc. The Veesler laboratory and the Sallusto laboratory have received sponsored research agreements from Vir Biotechnology Inc. The other authors declare no competing interests. **Data and materials availability:** The crystal structure of S2P6-bound SARS-CoV-2 stem helix peptide was deposited to the protein databank with accession number PDB 7RNJ. The cryoEM structure of SARS-CoV-2 spike protein bound to the S2P6 and S2M11 Fab fragments was deposited to the electron microscopy databank with accession number EMD-24533. Materials generated in this study will be made available on request, but may require a completed materials transfer agreement signed with Vir Biotechnology or the University of Washington. This work is licensed under a Creative Commons Attribution 4.0 International (CC BY 4.0) license, which permits unrestricted use, distribution, and reproduction in any medium, provided the original work is properly cited. To view a copy of this license, visit https://creativecommons.org/licenses/by/4.0/. This license does not apply to figures/photos/artwork or other content included in the article that is credited to a third party; obtain authorization from the rights holder before using such material.

## SUPPLEMENTARY MATERIALS

science.sciencemag.org/cgi/content/full/science.abj3321/DC1
Materials and Methods
Figs. S1 to S10
Tables S1 and S2
References (*61*–*86*)
MDAR Reproducibility Checklist

6 May 2021; accepted 29 July 2021
Published online 3 August 2021
10.1126/science.abj3321

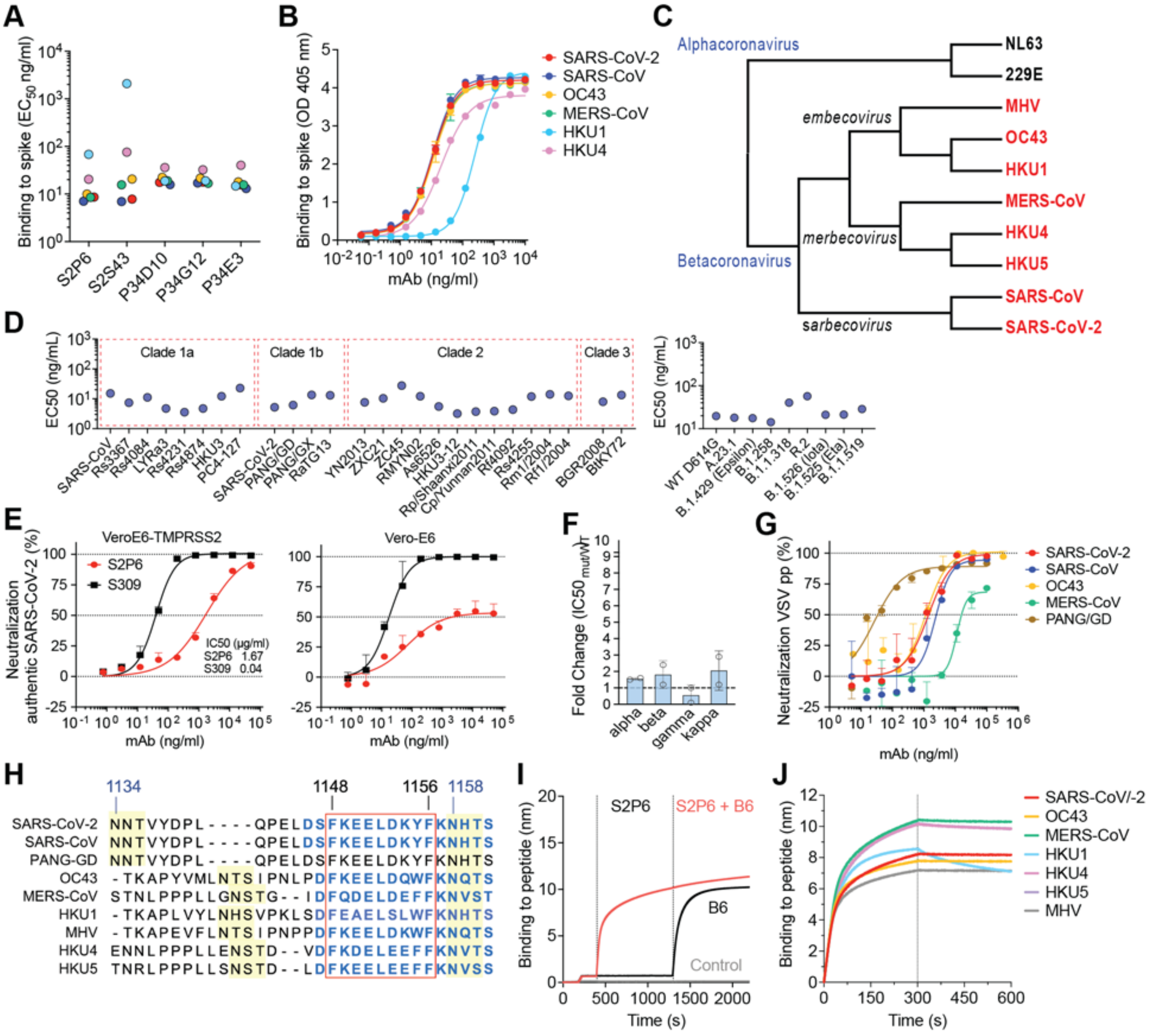
A
Binding to spike (EC50 ng/ml)
S2P6
S2S43
P34D10
P34G12
P34E3
B
Binding to spike (OD 405 nm)
mAb (ng/ml)
SARS-CoV-2
SARS-CoV
OC43
MERS-CoV
HKU1
HKU4
C
Alphacoronavirus
NL63
229E
embecovirus
MHV
OC43
HKU1
MERS-CoV
merbecovirus
HKU4
HKU5
Betacoronavirus
SARS-CoV
sarbecovirus
SARS-CoV-2
D
EC50 (ng/mL)
Clade 1a
Clade 1b
Clade 2
Clade 3
SARS-CoV
Rs3367
Rs4084
LYRa3
Rs4231
Rs4874
HKU3
PC4-127
SARS-CoV-2
PANG/GD
PANG/GX
RaTG13
YN2013
ZXC21
ZC45
RMYN02
As6526
HKU3-12
Rp/Shaanxi2011
Cp/Yunnan2011
Rf4092
Rs4255
Rm1/2004
Rf1/2004
BGR2008
BtKY72
EC50 (ng/mL)
WT D614G
A.23.1
B.1.429 (Epsilon)
B.1.258
B.1.1.318
R.2
B.1.526 (Iota)
B.1.525 (Eta)
B.1.1.519
E
VeroE6-TMPRSS2
Neutralization authentic SARS-CoV-2 (%)
S2P6
S309
IC50 (μg/ml)
S2P6 1.67
S309 0.04
mAb (ng/ml)
Vero-E6
mAb (ng/ml)
F
Fold Change (IC50 mut/WT)
alpha
beta
gamma
kappa
G
Neutralization VSV pp (%)
SARS-CoV-2
SARS-CoV
OC43
MERS-CoV
PANG/GD
mAb (ng/ml)
H
1134
1148
1156
1158
SARS-CoV-2 NNTVYDPL----QPELDSFKEELDKYFKNHTS
SARS-CoV NNTVYDPL----QPELDSFKEELDKYFKNHTS
PANG-GD NNTVYDPL----QPELDSFKEELDKYFKNHTS
OC43 -TKAPYVMLNTSIPNLPDFKEELDQWFKNQTS
MERS-CoV STNLPPPLLGNSTG--IDFQDELDEFFKNVST
HKU1 -TKAPLVYLNHSVPKLSDFEAELSLWFKNHTS
MHV -TKAPEVFLNTSIPNPPDFKEELDKWFKNQTS
HKU4 ENNLPPPLLENSTD--VDFKDELEEFFKNVTS
HKU5 TNRLPPPLLSNSTD--LDFKEELEEFFKNVSS
I
Binding to peptide (nm)
S2P6
S2P6 + B6
B6
Control
Time (s)
J
Binding to peptide (nm)
SARS-CoV/-2
OC43
MERS-CoV
HKU1
HKU4
HKU5
MHV
Time (s)

**Fig. 1. The S2P6 cross-reactive mAb broadly neutralizes β-coronaviruses from three subgenera.** (**A**) Binding avidity (EC50) of 5 mAbs to prefusion coronavirus S trimer ectodomains as determined by ELISA. (**B**) S2P6 ELISA binding curves showing the full titration. One representative experiment out of two is shown. (**C**) Cladogram of representative α- (black) and β-coronavirus (red) S glycoprotein amino acid sequences inferred via maximum likelihood analysis. (**D**) Flow cytometry analysis of S2P6 binding (from 10 to 0.22 μg/ml) to a panel of 26 S glycoproteins representative of all *sarbecovirus* clades (left) and 8 SARS-CoV-2 variants (right) displayed as EC50 values. (**E**) Neutralization of authentic SARS-CoV-2 by S2P6 determined using VeroE6-TMPRSS2 (left) or VeroE6 (right) cells. The S309 mAb that binds RBD site IV (*38*) is included for comparison. Mean ± s.d. of triplicates from one representative experiment out of three is shown. (**F**) S2P6-mediated neutralization of SARS-CoV-2 B.1.1.7 (alpha) S, B.1.351 (beta) S, P.1 (gamma) S and B1.617.1 (kappa) S VSV pseudotypes represented as IC50 fold change relative to wildtype (D614G) S VSV pseudotype. Individual values for each of the two replicates are shown as open circles, the mean as a colored bar and the SD as error bars. (**G**) S2P6-mediated neutralization of VSV pseudotyped with various β-coronavirus S glycoproteins. Error bars indicate standard deviation of triplicates. IC50 values: 2.4 μg/ml, 1.4 μg/ml, 17.1 μg/ml, 1.3 μg/ml and 0.02 μg/ml for SARS-CoV, SARS-CoV-2, MERS-CoV, OC43 and PANG/GD, respectively. (**H**) Alignment of β-coronavirus stem helix amino acid sequences with the S2P6 epitope boxed. Residue numbering is shown according to SARS-CoV-2 S. The sequences of the peptides used in this study are shown in blue and N-linked glycosylation sequons are highlighted in yellow. (**I**) Competition assay for S2P6/B6 binding to the SARS-CoV-2/SARS-CoV stem helix peptide (herein defined as SARS-CoV/-2). B6 binding in presence of S2P6 (red line), B6 binding in absence of S2P6 (black line) and the control lacking the SARS-CoV/-2 stem helix peptide (grey line) are shown. (**J**) Kinetics of S2P6 binding to a panel of biotinylated β-coronavirus stem helix peptides immobilized at the surface of biolayer interferometry biosensors.

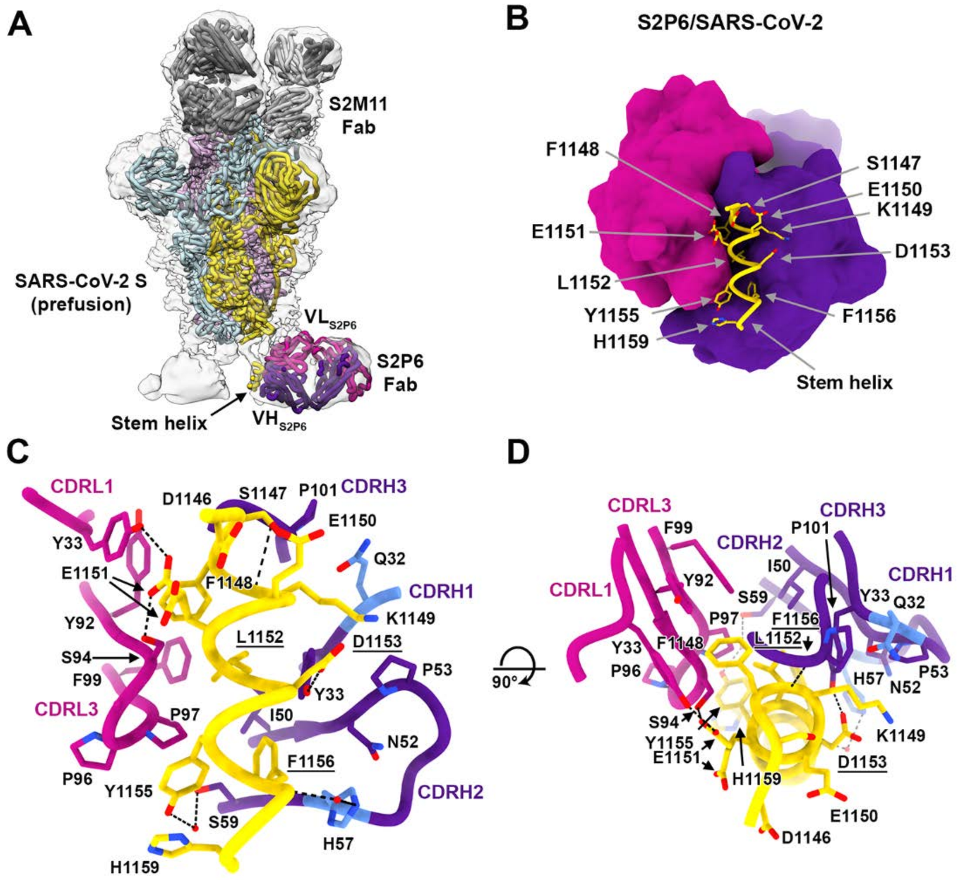


Fig. 2. **Structural basis for the broad S2P6 cross-reactivity with the conserved coronavirus stem helix peptide.** (A) Composite model of the S2P6-bound SARS-CoV-2 S cryoEM structure and of the S2P6-bound stem helix peptide crystal structure docked in the cryoEM map (transparent gray surface). SARS-CoV-2 S protomers are colored pink, cyan and gold, the S2P6 Fab heavy and light chains are colored purple and magenta and the S2M11 Fab heavy and light chains are colored dark and light gray, respectively. (B) Crystal structure of the S2P6 Fab (surface rendering) in complex with the SARS-CoV-2 S stem helix peptide (yellow ribbon with side chains rendered as sticks). (C and D) Ribbon diagram in two orthogonal orientations of the S2P6 Fab bound to the SARS-CoV-2 S stem helix peptide showing a conserved network of interactions. Only key interface residues and the S2P6 CDR loops are shown for clarity. Residues Q32 and H57, that are mutated during affinity maturation of the S2P6 heavy chain, are colored blue. Hydrogen bonds are indicated with dashed lines. Residues substituted in the escape mutants isolated are underlined.

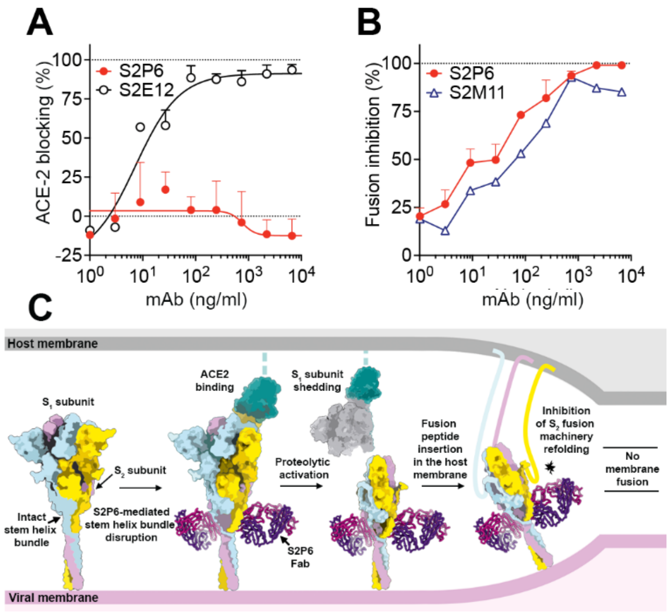


**Fig. 3. S2P6 binding disrupts the stem helix bundle and sterically inhibits membrane fusion.** (**A**) SARS-CoV-2 S binding to ACE2 in the presence of mAb S2P6 analyzed by ELISA. S2E12 was included as a positive control. (**B**) S2P6 inhibition of cell-cell fusion using Vero-E6 cells transfected with SARS-CoV-2 S. S2M11 was included as a positive control. Inhibition of fusion values are normalized to the percentage of fusion without mAb (100%) and to that of fusion of non-transfected cells (0%). (**C**) Proposed mechanism of inhibition mediated by the S2P6 mAb. S2P6 binds to the hydrophobic core of the stem helix bundle and disrupts its quaternary structure. S2P6 binding likely prevents $S_2$ subunit refolding from the pre- to the post-fusion state and blocks viral entry.

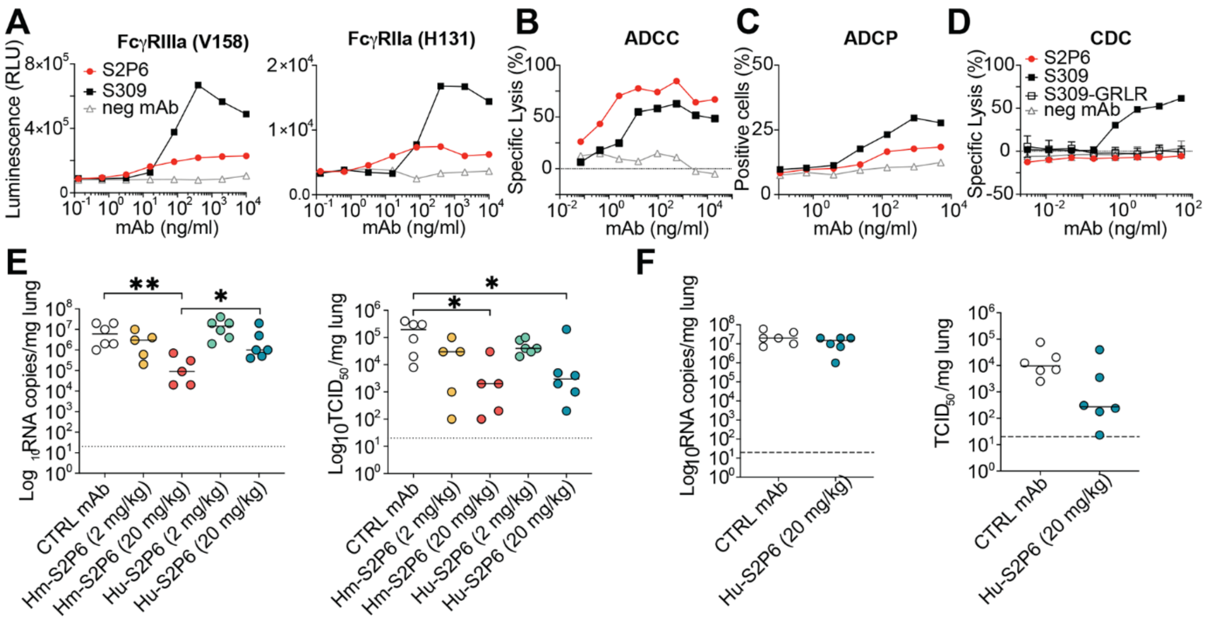


**Fig. 4. S2P6 activates effector functions and reduces SARS-CoV-2 lung burden in Syrian hamsters.** (**A**) NFAT-driven luciferase signal induced in Jurkat cells stably expressing FcγRIIIa (V158, left) or FcγRIIa (V131, right) upon S2P6 binding to full-length wild-type SARS-CoV-2 S expressed at the surface of CHO target cells. S309 is included as positive control. RLU, relative luminescence unit. (**B**) mAb-mediated ADCC using SARS-CoV-2 CHO-K1 cells (genetically engineered to stably express a HaloTag-HiBit) as target cells and PBMC as effector cells. The magnitude of NK cell–mediated killing is expressed as percentage of specific lysis. (**C**) mAb-mediated ADCP using Cell-Trace-Violet-labeled PBMCs as a source of phagocytic cells (monocytes) and PKH67–fluorescently labeled S-expressing CHO cells as target cells. The *y*-axis indicates percentage of monocytes double-positive for anti-CD14 (monocyte) marker and PKH67. (**D**) Lysis of SARS-CoV-2 S stably transfected CHO cells by mAbs in the presence of complement. S309 was included as positive control, S309-GRLR with diminished FcγR binding capacity and an unrelated mAb (neg mAb) were used as negative controls. (**E**) Syrian hamsters were administered with the indicated amount of S2P6 mAb harboring either a hamster (Hm-S2P6) or a human (Hu-S2P6) constant region before intranasal challenge with prototypic SARS-CoV-2 (Wuhan-1 related). An irrelevant mAb (MGH2 against *P. falciparum* CSP) at 20 mg/kg was used as negative control (*60*). Viral RNA loads and replicating virus titers are shown on the left and right, respectively. (**F**) Prophylactic administration of Hu-S2P6 at 20 mg/kg in hamsters challenged with SARS-CoV-2 B.1.351 (beta) VOC. Viral RNA loads and replicating virus titers are shown on the left and right, respectively. * P<0.05, ** P<0.01 Mann-Whitney test.

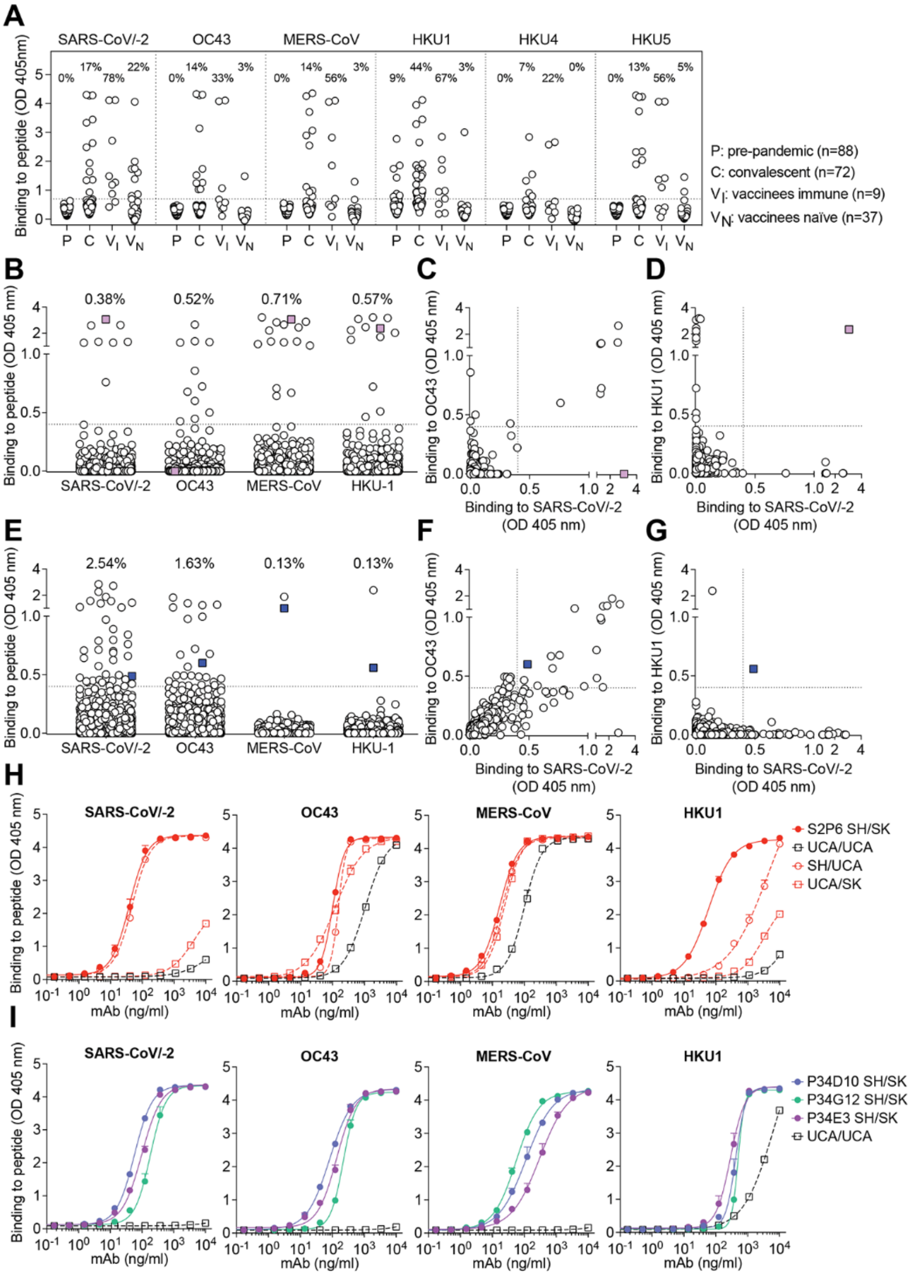


**Fig. 5. Stem helix-directed Abs are rare, of narrow specificities and enhance their binding affinity and cross-reactivity through somatic mutations.** (**A**) Binding of pre-pandemic (P, n=88), COVID-19 convalescent (C, n=72), vaccinees immune (VI, n=9) and vaccinees naïve (VN, n=37) plasma Abs (diluted 1:10) to immobilized β-coronavirus stem helix peptides analyzed by ELISA. A cutoff of 0.7 was determined based on signal of pre-pandemic samples and binding to uncoated ELISA plates (horizontal dash line). The fraction of samples for which binding above the cutoff was detected is indicated. (**B** to **G**) Analysis of memory B cell specificities for β-coronavirus stem helix peptides. Each dot represents a well containing oligoclonal B cell supernatant screened for the presence of stem helix peptide binding IgG Abs using ELISA. Samples obtained from 21 COVID-19 convalescent individuals (B-D) and 16 vaccinees (E-G). Pairwise reactivity comparison is shown for SARS-CoV/-2 and OC43 (C and F) and SARS-CoV/-2 and HKU1 (D and G). Cultures cross-reactive with at least three peptides are highlighted in color. A cutoff of 0.4 is indicated by a horizontal dash line. The fraction of wells for which binding above the cutoff was detected is indicated. (**H** and **I**) Binding to stem helix peptides of S2P6 (H) harboring mature (SH/SK), fully germline reverted (UCA/UCA), germline reverted heavy chain paired with mature light chain (UCA/SK), mature heavy chain paired with germline reverted light chain (SH/UCA) and of P34D10, P34G12 and P34E3 (I) harboring either mature (SH/SK) or germline reverted (UCA/UCA) sequences.
