# Supplementary material for: Broad betacoronavirus neutralization by a stem helix–specific human antibody
Source: Science. 2021 Aug 3;373(6559):1109–16. doi: 10.1126/science.abj3321 (PMC9268357; doi:10.1126/science.abj3321)
Supplement: Supplementary file 2 — Materials and Methods Figs. S1 to S10 Tables S1 and S2 References (61–86) [file science.abj3321_sm.pdf]

Science AAAS

Supplementary Materials for

Broad betacoronavirus neutralization by a stem helix–specific human antibody

Dora Pinto *et al.*

Corresponding authors: Davide Corti, dcorti@vir.bio; David Veesler, dveesler@uw.edu

Science **373**, 1109 (2021)
DOI: 10.1126/science.abj3321

The PDF file includes:

Materials and Methods
Figs. S1 to S10
Tables S1 and S2
References

Other Supplementary Material for this manuscript includes the following:

MDAR Reproducibility Checklist

Materials and Methods

Cell lines

Cell lines used in this study were obtained from ATCC (HEK293T and Vero-E6) or ThermoFisher Scientific (ExpiCHO cells, FreeStyle™ 293-F cells and Expi293F™ cells).

Sample donors

Samples were obtained from cohorts of individuals enrolled before June 2019 (pre-pandemic), of SARS-CoV-2 infected individuals or of vaccinated individuals immunized with Moderna or Pfizer/BioNTech BNT162b2 vaccines under study protocols approved by the local Institutional Review Boards (Canton Ticino Ethics Committee, Switzerland, the Ethical committee of Luigi Sacco Hospital, Milan, Italy and WCG North America, Princeton, NJ, US). All donors provided written informed consent for the use of blood and blood components (such as human peripheral blood mononuclear cells (PBMCs), sera or plasma) and were recruited at hospitals or as outpatients. PBMCs were isolated from blood by Ficoll density gradient centrifugation and either used freshly or stored in liquid nitrogen for later use. Sera were obtained from blood collected using tubes containing clot activator, followed by centrifugation and stored at -80°C.

AMBRA (antigen-specific memory B cell repertoire analysis) of IgG antibodies

Replicate cultures of total unfractionated PBMC from SARS-CoV-2 infected or vaccinated individuals were seeded in 96 U-bottom plates (Corning) in RPMI1640 supplemented with 10% Hyclone, sodium pyruvate, MEM non-essential amino acid, stable glutamine and Penicillin-Streptomycin. Memory B cell stimulation and differentiation was induced by adding 2.5 μg/ml R848 (3 M) and 1000 U/ ml human recombinant IL-2 for 10 days at 37 °C 5% CO_2. The cell culture supernatants were collected for further analysis.

Antibody discovery and expression

Antigen specific IgG^+ memory B cells were isolated and cloned from total PBMCs of convalescent individuals. Abs VH and VL sequences were obtained by reverse transcription PCR (RT-PCR) and mAbs were expressed as recombinant human IgG1, carrying the half-life extending M428L/N434S (LS) mutation in the Fc region or Fab fragment. ExpiCHO cells were transiently transfected with heavy and light chain expression vectors as previously described (*26*). For in vivo experiments in Syrian hamsters, S2P6 was produced with a Syrian hamster IgG2 constant region. Using the Database IMGT (http://www.imgt.org), the VH and VL gene family and the number of somatic mutations were determined by analyzing the homology of the VH and VL sequences to known human V, D and J genes. UCA sequences of the VH and VL were constructed using IMGT/V-QUEST.

MAbs affinity purification was performed on ÄKTA Xpress FPLC (Cytiva) operated by UNICORN software version 5.11 (Build 407) using HiTrap Protein A columns (Cytiva) for full length human and hamster mAbs and CaptureSelect CH1-XL MiniChrom columns (ThermoFisher Scientific) for Fab fragments, using PBS as mobile phase. Buffer exchange to the appropriate formulation buffer was performed with a HiTrap Fast desalting column (Cytiva). The final products were sterilized by filtration through 0.22 μm filters and stored at 4ºC.

Flow cytometry of antibody on S Protein expressing ExpiCHO-S cells

For Expi-CHO cell transient transfection, S plasmids (*26*, *61*) were diluted in cold OptiPRO SFM, mixed with ExpiFectamine CHO Reagent (Life Technologies, A29130) and added to the cells seeded at $6x10^6$ cells/ml in a volume of 5 ml in a 50 ml bioreactor. Transfected cells were incubated at 37°C, 8% CO_2 with an orbital shaking speed of 209 rpm (orbital diameter of 25 mm) for 42 hours. To test mAb binding, transfected ExpiCHO cells were collected, washed twice in wash buffer (1% w/v solution of Bovine Serum Albumin (BSA; Sigma) in PBS, 2 mM EDTA) and distributed at 60,000 cells/well into 96 U-bottom plates (Corning). mAb serial dilutions from 10 µg/ml were added onto cells for 30 minutes on ice and, after two washes, Alexa Fluor647-labelled Goat Anti-Human IgG (Jackson Immunoresearch, 109-606-098) was used for detection. After 15 minutes of incubation on ice, cells were washed twice and mAb binding analyzed by flow cytometry using a ZE5 Cell Analyzer (Biorard).

Protein expression and purification

SARS-CoV-2 S 2P, SARS-CoV S 2P, MERS-CoV S 2P, OC43 S, HKU1 S2P, and HKU4 S2P ectodomains were produced as previously described (*2*, *11*, *14*, *62*, *63*). SARS-CoV-2 S D614G, used for production of SARS-CoV-2 postfusion, contains a mu-phosphatase signal peptide beginning at 14Q, a mutated S_1/S_2 cleavage site (SGAR), and ends at residue K1211 followed by a TEV cleavage, foldon trimerization motif, and an 8X his tag in a pCMV vector. Briefly, spike glycoproteins were produced in Expi293F cells grown in suspension using Expi293 expression medium (Life Technologies) at 37˚C in a humidified 8% CO_2 incubator rotating at 130 rpm. The cultures were transiently transfected using PEI with cells grown to a density of 3 million cells per mL and cultivated for 3 days. The supernatant was clarified and affinity purified using a 1 mL HisTrapFF column (Cytiva). To isolate post-fusion SARS-CoV-2 S, SARS-CoV-2 S D614G ectodomain was incubated for one hour with the S2X58 triggering Fab (*64*) and 1 µg/ml trypsin before size-exclusion chromatography purification using a Superose 6 Increase 10/24 column (Cytiva). Purified protein was concentrated, quantified using absorption at 280 nm, and flash frozen in Tris-saline (20 mM Tris pH 8.0, 100 mM NaCl).

Enzyme-linked immunosorbent assay (ELISA)

96-well plates (Corning) were coated overnight at 4°C with recombinant proteins at 1 µg/ml or peptides at 8 µg/ml diluted in phosphate-buffered saline (PBS). Plates were blocked with a 1% w/v solution of Bovine Serum Albumin (BSA; Sigma) in PBS and serial dilutions of mAbs were added for 1 hour at room temperature. When testing human plasma or memory B-cell supernatants, plates were blocked with Blocker Casein (1% w/v) in PBS (Thermo Fisher Scientific) supplemented with 0.05% Tween 20. Plasma and memory B-cell supernatants (AMBRA testing) were then incubated for 1 hour at room temperature at a 1:10 and 1:2 dilution, respectively. After further wash, mAbs bound were revealed using an anti-human IgG coupled to alkaline phosphatase (Jackson Immunoresearch) incubated for 1 hour. Substrate (p-NPP, Sigma) was used for color development and plates read at 405 nm by a microplate reader (Biotek). The data were plotted with GraphPad Prism software.

For ELISA with plasma, cut-off value (OD=0.7) was determined based on signal of pre-pandemic samples and binding to uncoated ELISA plates. For AMBRA, cut off value (OD= 0.4) was determined as three times the mean OD values of negative wells.

Blockade of SARS-CoV-2 S binding to ACE2

SARS-CoV-2 S prefusion (final concentration 300 ng/ml) was incubated with 1 µg/ml of S309 mouse Fc-tagged mAb (S309-mFc) 30 minutes at 37°C before the addition of serially diluted S2P6 (from 20 µg/ml) and incubated for additional 30 minutes at 37°C. The complex S:S309:S2P6 was then added to a pre-coated hACE2 (2 µg/ml in PBS) 96-well plate MaxiSorp (Nunc) and incubated 1 hour at room temperature. Subsequently, the plates were washed and a goat anti-mouse IgG (Southern Biotech) coupled to alkaline phosphatase (Jackson Immunoresearch) added to detect SARS-CoV-2 S:S309-mFc binding. After further washing, the substrate (p-NPP, Sigma) was added, and plates read at 405 nm using a microplate reader (Biotek). The percentage of inhibition was calculated as follow: (1-((OD sample-OD neg ctr)/(OD pos. ctr-OD neg. ctr))*100.

Epitope identification and substitution scan

PEPperMAP Epitope Mapping (PEPperPRINT GmbH, Heidelberg, Germany) was performed to determine mAbs epitope through a pan-corona Spike protein Microarray covering the S proteins of all β-coronaviruses. Briefly, microarray containing 15-mer peptides (overlapping of 13-mer) was incubated with 10 µg/ml mAb for 16 hours at 4°C shaking at 140 rpm followed by staining with Goat anti-human IgG (H+L) DyLight680 for 45 minutes at room temperature. Microarray read-out was performed with a LI-COR Odyssey Imaging System at scanning intensities of 7/7 (red/green). Epitope substitution scan was performed on the identified epitope based on a stepwise single amino acid exchange on all amino acid positions. The mAbs binding to the generated microarray was performed as above.

Conservation analysis

Conservation analysis was performed as described previously (Pinto et al 2020). SARS-CoV-2 S sequences were obtained from GISAID (https://www.gisaid.org/) on Apr 22nd 2021, the other viruses sequences were obtained from NCBI Virus (https://www.ncbi.nlm.nih.gov/labs/virus/vssi/#/) in December 2020. The multiple sequences alignment was performed using MAFFT (https://mafft.cbrc.jp/alignment/software/) with the spike amino acid sequences as input.

SPR binding measurements

SPR binding measurements were performed using a Biacore T200 instrument using anti-AviTag pAb covalently immobilized on CM5 chips to capture S ECDs except the Cytiva Biotin CAPture kit was used to capture biotinylated OC43 S ECD. Running buffer was Cytiva HBS-EP+ (pH 7.4) or 20 mM phosphate pH 5.4, 150 mM NaCl, 0.05% P-20, for neutral or acidic pH experiments, respectively. All measurements were performed at 25 °C. S2P6 Fab or IgG concentrations were 11, 33, 100, and 300 nM run as single-cycle kinetics. Double reference-

subtracted data were fit to a binding model using Biacore Evaluation software. All data for SARS-CoV-2 S, SARS-CoV S, and OC43 S were fit to a 1:1 binding model. Data for MERS S were fit to a Heterogeneous Ligand binding model, due to a kinetic phase with very slow dissociation presumed to be an artifact; the lower affinity of the two KDs returned by the fit is reported as the KD of the S2P6:MERS S interaction and is indicated to be approximate (the Rmax associated with the higher affinity kinetic phase is proportional to the magnitude of the final signal above baseline). Data for HKU1 S were fit to a steady-state binding model, because of the low signal and fast approach to equilibrium within each association phase; the reported KD is indicated to be approximate. IgG binding data yield an "apparent KD" due to avidity.

Neutralization of authentic SARS-CoV-2 virus

For SARS-CoV-2 neutralization experiments, cells were cultured in DMEM (Gibco 11995-040) supplemented with 10% FBS (VWR 97068-085 lot#345K19) and 100 U/ml Penicillin-Streptomycin (Gibco 15140-122). Cells were seeded in black, 96-well glass bottom plates (Cellvis P96-1.5H-N) at a density of 20,000 cells/well. In a BSL3 facility, serial dilutions of mAbs (1:4) were incubated with 200 PFU (plaque forming units, corresponding to a multiplicity of infection of 0.01) of authentic SARS-CoV-2 (isolate USA-WA1/2020, passage 3, passaged in Vero-E6 cells) for 30 minutes at 37°C. After removal of cell culture supernatants, cells were infected with the virus:mAb mixtures and incubated for 20 hours at 37°C. Cells were then fixed with 4% paraformaldehyde (Electron Microscopy Sciences, 15714-S) in PBS (Gibco 10010-031) for 30 minutes, permeabilized with 0.1% Triton X-100 (Sigma, X100-500ML) for 30 minutes, and stained with Human SARS Coronavirus Nucleoprotein/NP Ab, Rabbit Mab (Sino Biological, 40143-R001) at a dilution of 1:2000 dilution in 2% milk (RPI, M17200-500.0) for 1 hour. Subsequently, cells were stained with Goat anti-Rabbit IgG (H+L) AF647 (Invitrogen, Cat. A21245 Lot. 223 2862) at a dilution of 1:1000 and 2 ug/ml Hoechst 33342 in 2% milk for 1 hour. Plates were imaged with an automated microscope (Cytation5, Biotek), and nuclei and cells positive for the SARS-CoV-2 Nucleoprotein were quantified using the supplied Gen5 software.

VSV pseudotype virus production and neutralization

Sarbecovirus spike cassettes with a C-terminal deletion of 19 amino acids (D19) were synthesized and cloned into mammalian expression constructs (pcDNA3.1(+) or pTwist-CMV) for the following Sarbecoviruses: SARS-CoV-2 (Accession QOU99296.1), SARS-CoV-1 (Accession AAP13441.1), hCoV-19/pangolin/Guangdong/1/2019 (GD19, Accession QLR06867.1), and Middle East respiratory syndrome-related coronavirus (MERS, Accession YP_009047204). To generate pseudotyped VSV, 293T Lenti-X packaging cells (Takara, 632180) were seeded in 15 cm dishes such that the cells would be 80% confluent the following day. Cultures were then transfected with various S expression plasmids using TransIT-Lenti transfection reagent (Mirus, 6600) according to the manufacturer's instructions. 24 hours after transfection, the packaging cells were infected with VSV-G*ΔG-luciferase (Kerafast, EH1020-PM). 48 hours after infection the supernatant containing Sarbecovirus pseudotyped VSV-luc was collected, centrifuged at 1000 × g for 5 minutes, aliquoted and frozen at −80°C.

To perform pseudotype neutralization assays, VeroE6-TMPRSS2 cells were used for VSV-SARS-CoV-2, VSV-SARS-CoV-1, and VSV-GD19 and Huh7 cells were used for VSV-MERS. Cells were seeded into clear bottom white-walled 96-well plates at 20,000 cells/well. The following day, 1:3 serial dilutions of Ab were prepared in DMEM and pseudotyped VSVs (final dilution 1:20) were added to each mAb dilution and incubated for 1 hour at 37°C. Media was removed from the cells and replaced with 50 µl of pseudotype:mAb complex and one hour post-infection, 50 µl of complete culture media was added to the cells and incubated overnight at 37°C. The media from infected cells was then removed and 100 µl of 1:1 diluted PBS:Bio-Glo (Promega, G7940) luciferase substrate was added to each well. The plates were shaken at 300 rpm at room temperature for 10 minutes and relative light units (RLUs) were then read on an EnSight microplate reader (Perkin Elmer). Percent neutralization was determined by subtracting the mean background (uninfected cells with luciferase substrate alone) values of 6 wells per plate from all data points. Percent neutralization for each mAb concentration was calculated relative to control wells receiving no mAb for each plate. Percent neutralization data were analyzed using GraphPad Prism. Absolute IC_{50} values were calculated by fitting a curve using a variable slope 4 parameter non-linear regression model and values were interpolated from the curve at y=50.

Production of OC43 S (AAT84354.1) pseudotyped VSV virus and neutralization assays was performed similarly to previously described (*14*). Briefly, HEK-293T cells at 70~80% confluency were transfected with the pCDNA3.1 expression vectors encoding full-length OC43 S harboring a truncation of the 17 C-terminal residues along with a fusion to Ha-tag and the bovine coronavirus hemagglutinin esterase protein Fc-tagged at molar ratios of 7:1. The day after, cells were transduced with VSVΔG/Fluc (*25*). After 2 h, infected cells were washed four times with DMEM before adding medium supplemented with anti-VSV-G antibody (I1- mouse hybridoma supernatant diluted 1 to 25, from CRL- 2700, ATCC). Supernatant was harvested 18-24 h post-inoculation, clarified from cellular debris by centrifugation at 2,000 x g for 5 min and concentrated 10 times using a 30 kDa cut off membrane and aliquoted and frozen at -80°C until use in neutralization experiments. For viral neutralization, stable HRT-18G cells (ATCC) in DMEM supplemented with 10% FBS, 1% PenStrep were seeded at 40,000 cells/well into clear bottom white walled 96-well plates and cultured overnight at 37°C. Twelve-point 3-fold serial dilutions of S2P6 were prepared in DMEM and OC43 S VSV pseudoviruses were added 1:1 (v/v) to each dilution in the presence of anti-VSV-G antibody from I1- mouse hybridoma supernatant diluted 50 times (final volume: 50 µl). After 45 min incubation at 37℃, 40 µl of the mixture was added to the cells and 2 h post-infection, another 40 µL DMEM were added to avoid evaporation. After 17-20 h, 50 µL/well of One-Glo-EX substrate (Promega) were added to the cells and incubated in the dark for 5-10 min prior reading on a Varioskan LUX plate reader (ThermoFisher). Data was processed using GraphPad Prism v9.0.

Selection of VSV-SARS-CoV-2 mAb escape mutants

Resistant virus selection

Cells were cultured in DMEM (Gibco 11995-040) supplemented with 10% FBS (VWR 97068-085 lot#345K19) and 100 U/ml Penicillin-Streptomycin (Gibco 15140-122). The day before infection, 250,000 VeroE6-TMPRSS2 cells were seeded in 12-well plates in 2 ml of DMEM (Gibco 11995-040) supplemented with 10% FBS (VWR 97068-085 lot#345K19) and 100 U/ml

Penicillin-Streptomycin (Gibco 15140-122) and incubated overnight at 37C. The next day, S2P6 was serially diluted 1:4 starting at 80 µg/ml in infection media (DMEM supplemented with 2% FBS and 20mM HEPES (Gibco, 15630-080)) and incubated with replication-competent VSV-SARS-CoV-2 (*27*) at MOI 2 for 1 hour at 37°C. A no Ab control was included to account for any tissue culture adaptations and quasispecies variability that may occur during virus replication. The mAb-virus complexes were adsorbed on the cells for 1 hour at 37°C, with manual rocking every 15 minutes. After adsorption, cells were washed with PBS and overlaid with infection media containing an equivalent amount of S2P6 as was used for the initial infection. Infection was monitored visually by microscopy for GFP expression and cytopathic effect (CPE) of the cells at day 1 and day 3 post-infection. At day 3 post-infection, when the no mAb control well reached >50% CPE, the well with the highest Ab concentration showing >20% CPE (in this case the 80 ug/ml well) was selected for passaging. The cell supernatant was centrifuged to remove cell debris, diluted 1:10 in infection medium and added to fresh VeroE6-TMPRRS2 cells with the same S2P6 concentration range and treatment as for the initial passage. Selection was stopped after two passages, after no virus neutralization was observed at the highest concentration tested.

Sequencing of S gene

Viral RNA was extracted from the supernatant of viral passages using the QIAamp Viral RNA Mini Kit (Qiagen, 52904) according to the manufacturer's instructions, without the addition of carrier RNA. Reverse transcription reactions were performed with 6 µl of purified RNA and random primers using the NEB ProtoScript II First Strand cDNA Synthesis Kit (NEB, E6560S), according to manufacturer's instructions. The resulting cDNA was used as a template for PCR amplification of the spike gene using the KapaBiosystems polymerase (KAPA HiFi HotStart Ready PCR Kit KK2601) with primers 5'- CGAGAAAAGGCATCTGGAG -3' and 5'-CATTGAACTCGTCGGTCTC -3'. Amplification conditions included an initial 3 minutes at 95°C, followed by 28 cycles with 20 seconds at 98°C, 15 seconds at 59°C and 72°C for 2 minutes, with a final 4 minutes at 72°C. PCR products were purified using AMPure XP beads (Beckman Coulter, A63881) following manufacturer's instructions. The size of the amplicon was confirmed by analyzing 2 µl of PCR products using the Agilent D5000 ScreenTape System (Agilent D5000 ScreenTape, 5067-5588, Agilent D5000, Reagents 5067-5589). Products were quantified by analyzing 2 µl with the Quant-iT dsDNA High-Sensitivity Assay Kit (Thermo Fisher, Q331120). Twenty ng of purified PCR product was used as input for library construction using the NEBNext Ultra II FS DNA Library Prep Kit (NEB, E6177S) following manufacturer's instructions. DNA fragmentation was performed for 13 minutes. NEBNext Multiplex Oligos for Illumina Dual Index Primer Set 1 (NEB, E7600S) was used for library construction, with a total of 6 PCR cycles. Libraries size was determined using the Agilent D1000 ScreenTape System (Agilent D1000 ScreenTape, 5067-5582, Agilent D5000 Reagents, 5067-5583) and quantified with the Quant-iT dsDNA High-Sensitivity Assay Kit. Equal amounts of each library were pooled together for multiplexing and 'Protocol A: Standard Normalization Method' of the Illumina library preparation guide was used to prepare 8 pM final multiplexed library with 1% PhiX spike-in for sequencing. The Illumina MiSeq Reagent Kit v3 (600-cycle) (Illumina, MS-102-300) was used for sequencing the libraries on the Illumina MiSeq platform, with 300 cycles for Read 1, 300 cycles for Read 2, 8 cycles for Index 1, and 8 cycles for Index 2.

Bioinformatic analysis

The average read length after running Illumina's Bcl2fastq command was ranging from 149 to 188bp on average per sample. For consistency across samples, paired-end reads were initially trimmed to 2X150bp and further cleaned to remove Illumina's adapter and low quality bases using Trimmomatic (*65*). Read alignment was performed with Burrows- Wheeler Aligner (BWA (*66*)) using a custom reference sequence. Variants were called with LoFreq upon indel realignment and base quality recalibration (*67*), using a frequency threshold of 1%. Two consecutive rounds of alignments and variant calling were performed, where the variants called during the first round at allelic frequency >50% were integrated in the reference for the second round in order to adjust alignment rate and variant calling accuracy. Variants were annotated with SnpEff (*68*). The reference sequence coordinates were mapped back to the SARS-CoV-2 Wuhan-Hu-1 sequence (NCBI: NC_045512.2) in order to match the reference sequence nomenclature. Extensive QCs were performed at read, alignment and variant level using FastQC , samtools, picard, mosdepth (*69*), bcftools (*70*), MultiQC (*71*) and in-house scripts, notably to remove variants that were consistently called at a static position in reads (such as the beginning or end of reads that were carrying it, rather than being randomly distributed throughout those reads.). An end-to-end workflow was automated using NextFlow (*72*). All programs are available through the Bioconda Initiative (*73*) (bioconda.github.io).

Crystallization and structure determination

Crystals of the S2P6 Fab/SARS-CoV-2 peptide complex were obtained using the sitting-drop vapor diffusion method at 20°C with a Fab concentration of 12 mg/ml and a 1.5-fold molar excess of peptide. A total of 150 nl S2P6 Fab/peptide solution in 20 mM Tris-HCl pH 7.5, 50 mM NaCl were mixed with 150 nl mother liquor containing 0.2 M ammonium sulfate, 0.1 M sodium acetate pH 4.6 and 25% (v/v) PEG Smear Broad (Molecular Dimensions). Crystals were flash frozen in liquid nitrogen. Data were collected at beamline 12-2 at the Stanford Synchrotron Radiation Lightsource facility in Stanford, CA. Data were processed with the XDS software package (Kabsch, 2010) for a final dataset of 2.67 Å in space group $P6_522$. The S2P6 Fab/peptide complex structure was solved by molecular replacement using a homology model of the S2P6 Fab built using the Molecular Operating Environment (MOE) software package from the Chemical Computing Group (https://www.chemcomp.com). Several subsequent rounds of model building and refinement were performed using Coot (*74*), ISOLDE (*75*), Refmac5 (*76*), Phenix (*77*) and MOE, to arrive at a final model for the complex.

Measurement of Fc-effector functions

MAb-dependent activation of human FcγRIIIa and FcγRIIa

Determination of mAb-dependent activation of human FcγRIIIa and FcγRIIa was performed using ExpiCHO cells stably expressing full-length wild-type SARS-CoV-2 spike (S) (target cells). Cells were incubated with different amounts of mAbs for 10 minutes before incubation with Jurkat cells stably expressing FcγRIIIa receptor (V158 variant) or FcγRIIa receptor (H131 variant) and NFAT-driven luciferase gene (effector cells) at an effector to target ratio of 6:1 for FcγRIIIa and 5:1 for FcγRIIa. Activation of human FcγRs was quantified by the luciferase signal produced as a result of NFAT pathway activation. Luminescence was measured after 21 hours of

incubation at 37˚C with 5% CO_2 with a luminometer using the Bio-Glo-TM Luciferase Assay Reagent according to the manufacturer's instructions (Promega, Cat. Nr.: G7018 and G9995).

Antibody-dependent cell cytotoxicity (ADCC)

ADCC assays were performed using SARS-CoV2 CHO-K1 cells (genetically engineered to stably express a HaloTag-HiBit-tagged) as target cells and PBMC as effector cells at a E:T ratio of 33:1. HiBit-cells were seeded at 3,000 cells/well and incubated for 16 hours at 37°C, while PBMCs isolated from fresh blood (VV donor) were cultivated overnight at 37°C 5% CO_2 in the presence of 5 ng/ml of IL-2. The day after, media was removed and titrated concentrations of mAbs were added before the addition of PBMCs at 100,000 cells/well. As 100% specific lysis, Digitonin at 100 ug/ml was used. After 4 hours of incubation at 37°C, ADCC was measured with Nano-Glo HiBiT Extracellular Detection System (Promega; Cat. Nr.: N2421) using a luminometer (Integration Time 00:30).

Antibody-dependent cellular phagocytosis (ADCP)

ADCP was performed using CHO cells stably expressing full-length wild-type SARS-CoV-2 S glycoprotein (target cells) fluorescently labelled with PKH67 Fluorescent Cell Linker Kits (Sigma Aldrich; Cat. Nr.: MINI67). Target cells were incubated with titrated concentrations of mAbs for 10 minutes, followed by incubation with PBMCs fluorescently labelled with Cell Trace Violet (Invitrogen, cat. no. C34557) after an overnight incubation in 5 ng/ml IL-2 (Recombinant Human Interleukin-2; ImmunoTools GmbH; Cat. Nr.: 11340027). An effector:target ratio of 20:1 was used. After an overnight incubation at 37°C, cells were stained with anti-human CD14-APC Ab (BD Pharmingen, cat. no. 561708, Clone M5E2) to stain monocytes. ADCP was determined by flow cytometry, gating on CD14+ cells that were double-positive for cell trace violet and PKH67.

Complement-dependent cytotoxicity (CDC)

CDC was performed on CHO cells stably expressing SARS-CoV-2 S glycoprotein (target cells) incubated with serial dilutions of mAbs for 10 minutes, followed by incubation with pre-adsorbed Low-Tox M Rabbit Complement (Cederlane Laboratories Limited; Cat. Nr.: CL3051) at a final dilution of 1:12. CDC was measured using lactate dehydrogenase (LDH) release as a readout according to the manufacturer's instructions (Cytotoxicity Detection Kit (LDH), Roche) after 3 hours of incubation at 37°C. In brief, plates were centrifuged for 4 minutes at 400 x g, and 20 µl of supernatant was transferred to a flat 384 well plate. LDH reagent was prepared and 20 µl were added to each well. Using a kinetic protocol, the absorbance at 490 nm and 650 nm was measured once every 2 minutes for 8 minutes, and the slope of the kinetics curve was used as result. The percent specific lysis was determined by applying the following formula: (specific release – spontaneous release) / (maximum release - spontaneous release) x 100. The spontaneous release is the level of lysis of target cells with complement (and without antibodies) and corresponds to the baseline. On the contrary the maximal release is the level of lysis of target cells with complement and 0.83% Triton.

S2P6 binding and S2P6/B6 competition experiments to different synthetic coronavirus S stem peptides

All biotinylated coronavirus stem helix peptides binding experiments were performed in PBS supplemented with 0.005 % Tween20 (PBST) at 30°C and 1,000 rpm shaking on an Octet Red instrument (Fortebio). For S2P6 binding to different stem helix peptides, 1 µg/ml biotinylated stem peptide (15- or 16-residue long stem peptide-PEG6-Lys-Biotin synthesized from Genscript) was loaded on SA biosensors to a threshold of 0.5 nm. Then, the system was equilibrated in PBST for 300 seconds prior to immersing the sensors in 0.1 µM S2P6 mAb, respectively, for 300 seconds prior to dissociation in buffer for 300 seconds. For S2P6-B6 competition, 1 µg/ml biotinylated to SARS CoV-2 peptide was loaded on SA biosensors to a threshold of 0.5 nm. The system was equilibrated in PBST for 180 seconds and each subsequent step was monitored for 900 seconds. The first sample biosensor was immersed in 0.1 µM mAb S2P6 prior to immersing the sample biosensor in a solution of 0.1 µM mAb S2P6 and B6, respectively. The second sample biosensor was immersed in PBST and subsequently in 0.1 µM mAb B6. To monitor unspecific binding, identical experiments were performed without loading stem peptides to the biosensors.

CryoEM sample preparation and data collection

1 mg/ml SARS-CoV-2 S 2P was incubated with 1.5-fold molar excess of S2M11 Fab for 30 minutes at 37°C (to promote the closed trimer conformation). Excess S2M11 Fab was removed from the sample using a centrifugal filter (amicon ultra, 100kDa cut off). Then a 2-fold molar excess of S2P6 Fab over SARS-CoV-2 S protomer was added to the solution and incubated for additional 45 minutes at 37°C. 3 µl sample were applied on to a freshly glow discharged UltrAUFoil Au 200 (R2/2) grid. Plunge freezing was performed using a TFS Vitrobot Mark IV (blot force: 0, blot time: 6.5 s, Humidity: 100 %, temperature: 23°C). Data were acquired using a FEI Titan Krios transmission electron microscope operated at 300 kV and equipped with a Gatan K3 Summit direct detector and Gatan Quantum GIF energy filter, operated in zero-loss mode with a slit width of 20 eV. Automated data collection was carried out using Leginon (*78*) at a nominal magnification of 105,000x with a pixel size of 0.4215Å. The dose rate was adjusted to 15 counts/pixel/s, and each movie was acquired in super-resolution mode fractionated in 75 frames of 40 ms. Tilted data collection (45° tilt) was performed to compensate for preferential specimen orientation and 6,015 micrographs were collected in a single session with a defocus range comprised between 0.5 and 5.0 µm.

CryoEM data processing

Movie frame alignment, estimation of the microscope contrast-transfer function parameters, particle picking and extraction were carried out using Warp (*79*)

. Particle images were extracted with a box size of 1024 $pixels^2$ binned to 256 $pixels^2$ yielding a pixel size of 1.686 Å. Two rounds of reference-free 2D classification were performed using cryoSPARC (*80*) to select well-defined particle images. Subsequently, one round of 3D classification with 25 iterations was carried out using Relion without imposing symmetry and using an initial ab initio model created in cryoSPARC.

The best subclasses were combined and non-uniform refinement (NUR), defocus refinement (DR) and NUR again performed in cryoSPARC. We then performed one round of global CTF refinement of beam-tilt, trefoil and tetrafoil parameters followed by another refinement cycle of NUR-DR-NUR. Selected particle images were then subjected to Bayesian polishing in Relion (*81*). During this step the box and pixel size were changed to 426 pixels and 1.201 Å, respectively, before performing another NUR-DR-NUR refinement cycle. We then performed one additional round of focused classification in Relion with 25 iterations, skipping the oriental assignment and using a mask covering the strongest S2P6 Fab density and a small part of the S stem to further separate distinct S2P6 Fab conformations. The best classes were combined and a final round of NUR performed. Reported resolutions are based on the gold-standard Fourier shell correlation (FSC) of 0.143 criterion and Fourier shell correlation curves were corrected for the effects of soft masking by high-resolution noise substitution (*82*).

CryoEM model building and analysis

UCSF Chimera (*83*) was used to fit atomic models into the cryoEM maps. The SARS-CoV-2 S EM structure in complex with the variable domain of the S2M11 Fab (PDB 7K43, residue 15-1140), the constant domain of the S2H14 Fab crystal structure and the S2P6-SARS-CoV-2 (residue 1146-1159) crystal structure were fit into the cryoEM map.

Fusion inhibition assay

For testing inhibition of spike-mediated cell–cell fusion Vero-E6 cells were seeded in 96 well plates at 20,000 cells/ well in 70 µl DMEM with high glucose and 2.4% FBS (Hyclone). After 16 hours, cells were transfected with SARS-CoV-2-S-D19_pcDNA3.1 as follows: for 10 wells, 0.57 µg plasmid SARS-CoV-2- S-D19_pcDNA3.1 were mixed with 1.68 µl X-tremeGENE HP in 30 µl OPTIMEM. After 15 minutes incubation, the mixture was diluted 1:10 in DMEM medium and 30 µl was added per well. A 4-fold serial dilution mAb was prepared and added to the cells, with a starting concentration of 20 µg/ml. The following day, 30 µl 5X concentrated DRAQ5 in DMEM was added per well and incubated for 2 hours at 37°C. Nine images of each well were acquired with a Cytation 5 equipment for analysis.

In vivo mAb testing using a Syrian hamster model

KU LEUVEN R&D has developed and validated a SARS-CoV-2 Syrian Golden hamster infection model (*39*).

SARS-CoV-2 virus production

The wt SARS-CoV-2 strain used in this study, BetaCov/Belgium/GHB-03021/2020 (EPI ISL 109 407976|2020-02-03), was recovered from a nasopharyngeal swab taken from an RT-qPCR confirmed asymptomatic patient who returned from Wuhan, China in the beginning of February 2020. A close relation with the prototypic Wuhan-Hu-1 2019-nCoV (GenBank accession 112 number MN908947.3) strain was confirmed by phylogenetic analysis. Infectious virus was isolated by serial passaging on HuH7 and Vero-E6 cells (*39*); passage 6 virus was used for the study described here. The titer of the virus stock was determined by end-point dilution on Vero-E6 cells by the Reed and Muench method (*84*). The variant strain B.1.351 (hCoV-19/Belgium/rega-1920/2021; EPI_ISL_896474, 2021-01-11) was isolated from nasopharyngeal swabs taken from a traveler returning to Belgium and developing respiratory symptoms. The

patients' nasopharyngeal swabs were directly subjected to sequencing on a MinION platform (Oxford Nanopore) (*85*).
Live virus-related work was conducted in the high-containment A3 and BSL3+ facilities of the KU Leuven Rega Institute (3CAPS), under licenses AMV 30112018 SBB 219 2018 0892 and AMV 23102017 SBB 219 20170589 according to institutional guidelines.

SARS-CoV-2 infection model in hamsters
Wildtype Syrian hamsters (Mesocricetus auratus) were purchased from Janvier Laboratories and were housed per two in ventilated isolator cages (IsoCage N Biocontainment System, Tecniplast) with ad libitum access to food and water and cage enrichment (wood block). Housing conditions and experimental procedures were approved by the ethical committee of animal experimentation of KU Leuven (license P065-2020). Female hamsters of 6-10 weeks old were anesthetized with ketamine/xylazine/atropine and inoculated intranasally with 50 µl containing 2×10^6 or 1×10^4 TCID50 for wt or B.1.351 variant, respectively. Treatment with mAb (human or hamster S2P6 (2-20 mg/kg) was initiated either 24 or 48 hours before infection by intraperitoneal injection. Hamsters were monitored for appearance, behavior and body weight. At day 4 post-infection, hamsters were euthanized by intraperitoneal injection of 500 µl Dolethal (200 mg/ml sodium pentobarbital, Vétoquinol SA). Lungs were collected, and viral RNA and infectious virus were quantified by RT-qPCR and end-point virus titration, respectively. Blood samples were collected before infection for pharmacokinetics analysis.

SARS-CoV-2 RT-qPCR
Hamster tissues were collected after sacrifice and were homogenized using bead disruption (Precellys) in 350 µl RLT buffer (RNeasy Mini kit, Qiagen) and centrifuged (10,000 rpm, 5 minutes) to pellet the cell debris. RNA was extracted according to the manufacturer's instructions. To extract RNA from serum, a NucleoSpin kit (Macherey-Nagel) was used. 4 µl out of 50 µl eluate were used as a template in RT-qPCR reactions. RT-qPCR was performed on a LightCycler96 platform (Roche) using the iTaq Universal Probes One-Step RTqPCR kit (BioRad) with N2 primers and probes targeting the nucleocapsid (*39*). Standards of SARS-CoV-2 cDNA (IDT) were used to express viral genome copies per mg tissue or per ml serum.

End-point virus titrations

Lung tissues were homogenized using bead disruption (Precellys) in 350 µl minimal essential medium and centrifuged (10,000 rpm, 5 minutes, 4°C) to pellet the cell debris. To quantify infectious SARS-CoV-2 particles, endpoint titrations were performed on confluent Vero-E6 cells in 96-well plates. Viral titers were calculated by the Reed and Muench method (*84*) using the Lindenbach calculator and were expressed as 50% tissue culture infectious dose (TCID50) per mg tissue.

MSD quantification of mAbs in sera of Syrian hamsters

For quantification of mAbs in hamster serum, a mAb specific against human Fc was used to capture human S2P6 or SARS-Cov2 Spike D614G protein was used to capture S2P6 with a hamster Fc onto standard plates (MSD, Meso Scale Discovery). Plates were washed, blocked with Casein in PBS (Thermo Fisher) and incubated for detection with mouse anti-human IgG (CH2 domain)-sulfo tag (from MSD and labelled with sulfo tag) or goat anti-hamster IgG(H+L)-

sulfo tag (Southern Biotech), respectively. After adding MSD gold read buffer, chemoluminescence signals were read using the MESO Quickplex SQ 120 instrument. Signals were directly proportional to the amount of S2P6 present in the samples.

A

	HEAVY CHAIN					LIGHT CHAIN				
mAb	IGHV	IGHD	IGHJ	CDR3-length (amino acid)	V identity (%)	IGLV	IGLJ	CDR3-length (amino acid)	V identity (%)	Time point from disease onset
S2P6	V1-46*01	D5-12*01	J4*02	11	95.14	KV3-20*01	K3*01	11	97.52	46
S2S43	V1-46*01	D5-12*01	J6*02	10	96.53	LV1-51*02	J1*01	11	94.74	39
P34D10	V3-30*03	D3-9*01	J6*02	14	90.28	LV2-23*02	J3*02	11	97.22	43
P34G12	V3-30*03	D3-9*01	J6*02	14	87.85	LV2-23*02	J3*02	11	94.1	43
P34E3	V3-30*03	D3-9*01	J6*02	14	86.46	LV2-23*02	J3*02	11	95.14	43

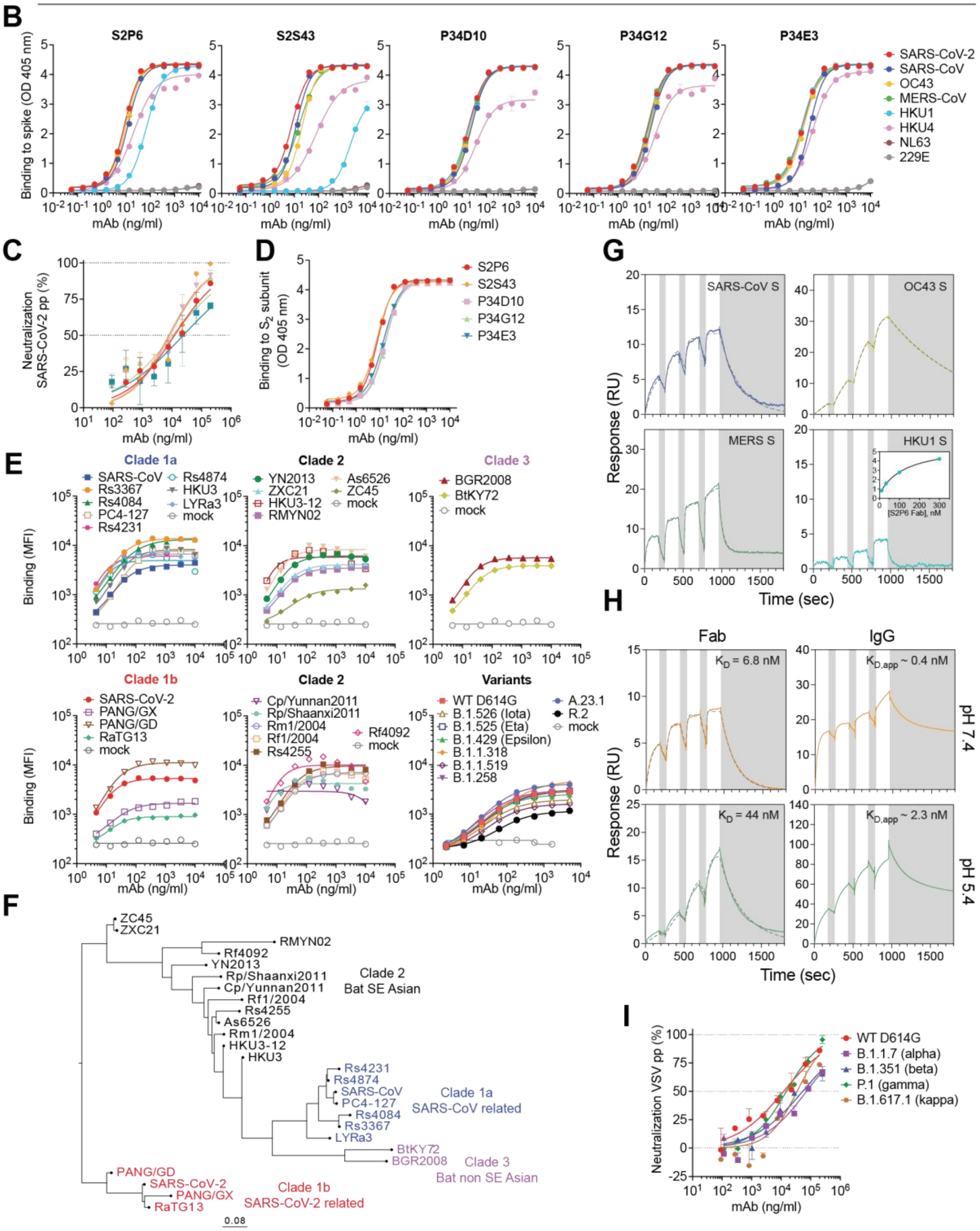

Fig. S1. Properties of the 5 cross-reactive mAbs isolated. (**A**) V(D)J usage, nucleotide sequence identity to germline genes, number of somatic mutations, and time interval between sample collection and mAb isolation. (**B**) Binding of identified mAbs to prefusion β-coronavirus S ectodomain trimers by ELISA. (**C**) Evaluation of mAb-mediated neutralization of SARS-CoV-2 S VSV pseudotype viruses. Error bars indicate standard deviation of technical duplicates. (**D**) Evaluation of mAb binding to the SARS-CoV-2 (post-fusion) S_2 subunit by ELISA. (**E**) Mean fluorescence intensity as measured in flow cytometry for S2P6 binding to a panel of 26 full-length S glycoproteins representative of all *sarbecovirus* clades and 8 SARS-CoV-2 variants transiently expressed at the surface of expiCHO cells. (**F**) Phylogenetic tree of sarbecovirus S glycoproteins used in this work inferred via maximum likelihood analysis of S amino acid sequences. (**G**) SPR analysis of S2P6 Fab binding to immobilized prefusion β-coronavirus S trimers. Data for HKU1 S were fit to a steady-state binding model (insert panel). (**H**) SPR analysis of S2P6 Fab and IgG binding to immobilized prefusion SARS-CoV-2 S ectodomain trimer at pH 7.4 and pH 5.4. Fits to a 1:1 binding model are an approximation for IgG binding due to bivalency. (**I**) S2P6 neutralization of VSV pseudotyped with SARS-CoV-2 S of several SARS-CoV-2 variants of concern (VOC) and the parental SARS-CoV-2 D614G S.

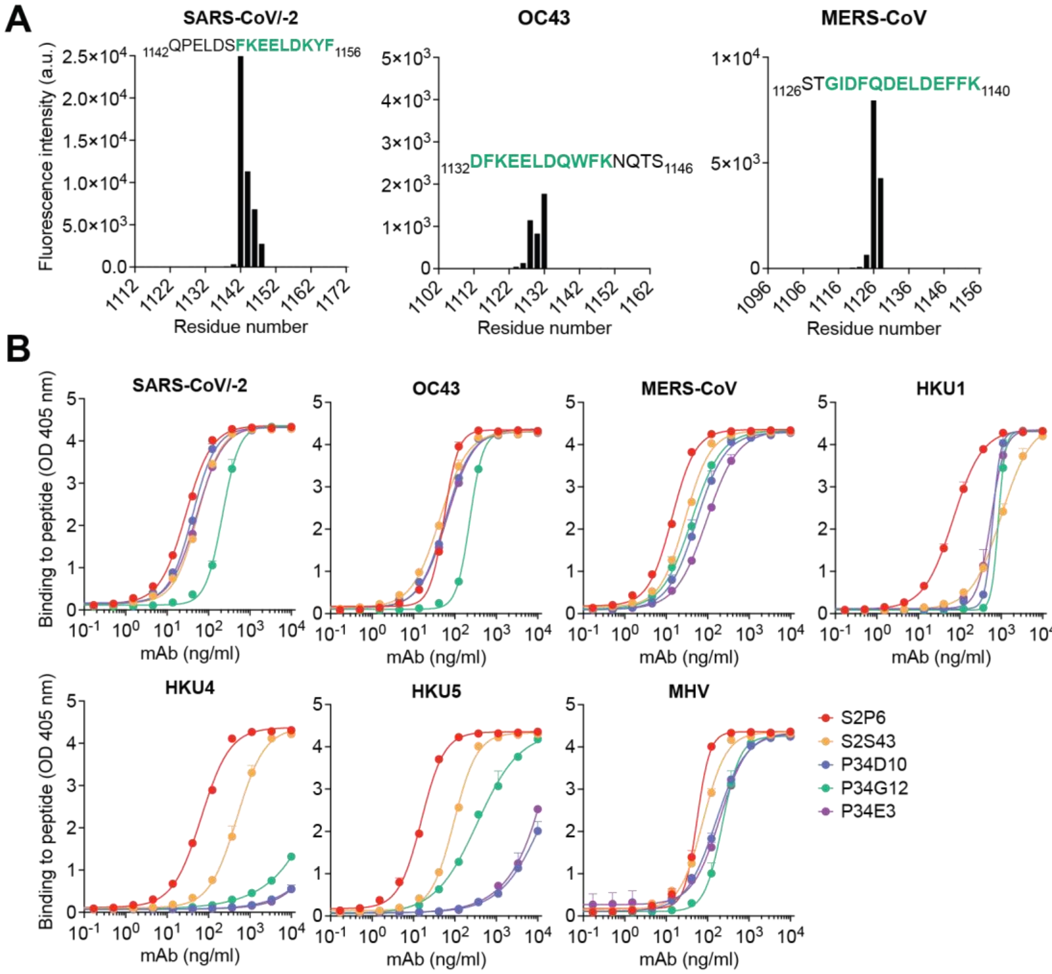

Fig. S2. Identification of the S2P6 epitope. (**A**) Binding of S2P6 to linear peptides (15-mer peptides overlapping by 13 residues) spanning the SARS-CoV/SARS-CoV-2 S, OC43 S and MERS-CoV S sequences. (**B**) Binding of identified mAbs to β-coronavirus S stem helix peptides by ELISA.

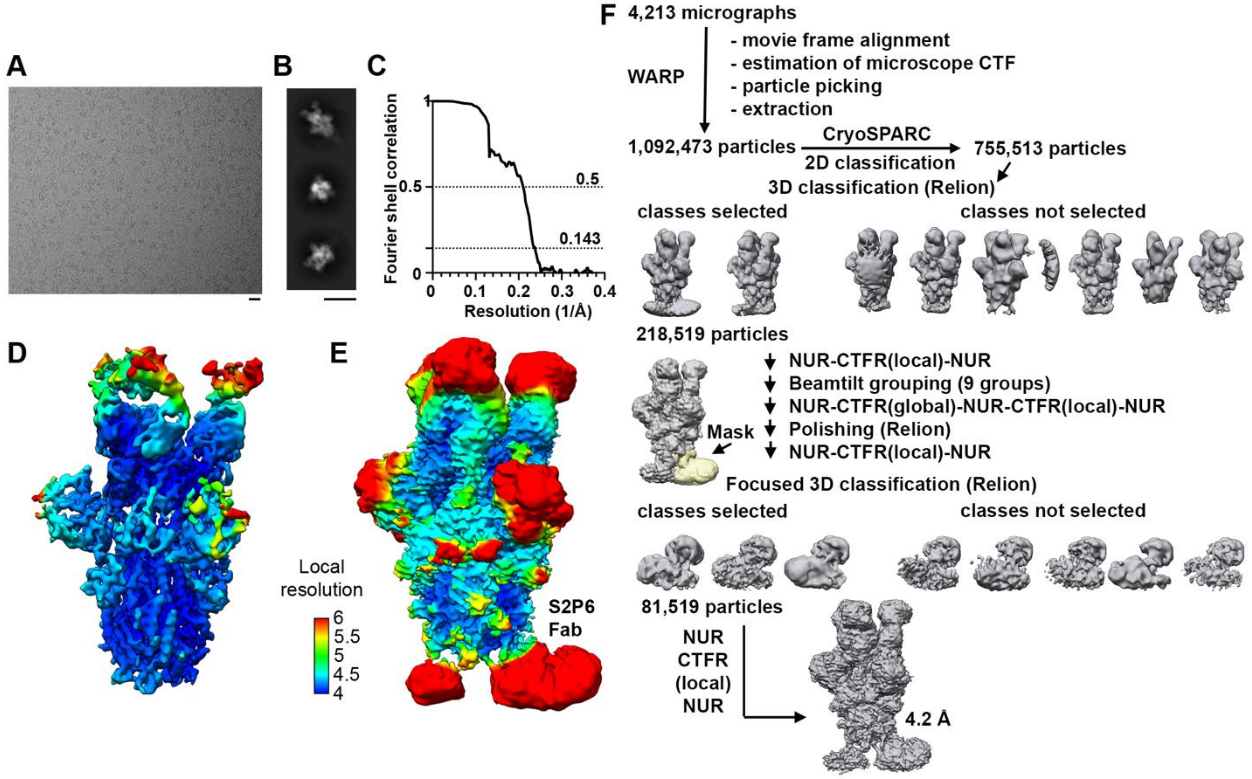

Fig. S3. **CryoEM data processing and validation of S2P6- and S2M11-bound SARS-CoV-2 S dataset.** (**A-B**) Representative electron micrograph (A) and class averages (B) of SARS-CoV-2 S in complex with the S2P6 and S2M11 Fabs. Scale bars: 200 Å. (**C**) Gold-standard Fourier shell correlation curve. The 0.143 and 0.5 cut-offs are indicated by horizontal dashed gray lines. (**D-E**) CryoEM map colored by local resolution computed using cryoSPARC shown at two distinct contour levels. **(F)** Cryo-EM data processing flow chart. CTFR: per-particle defocus refinement, NUR: non-uniform refinement.

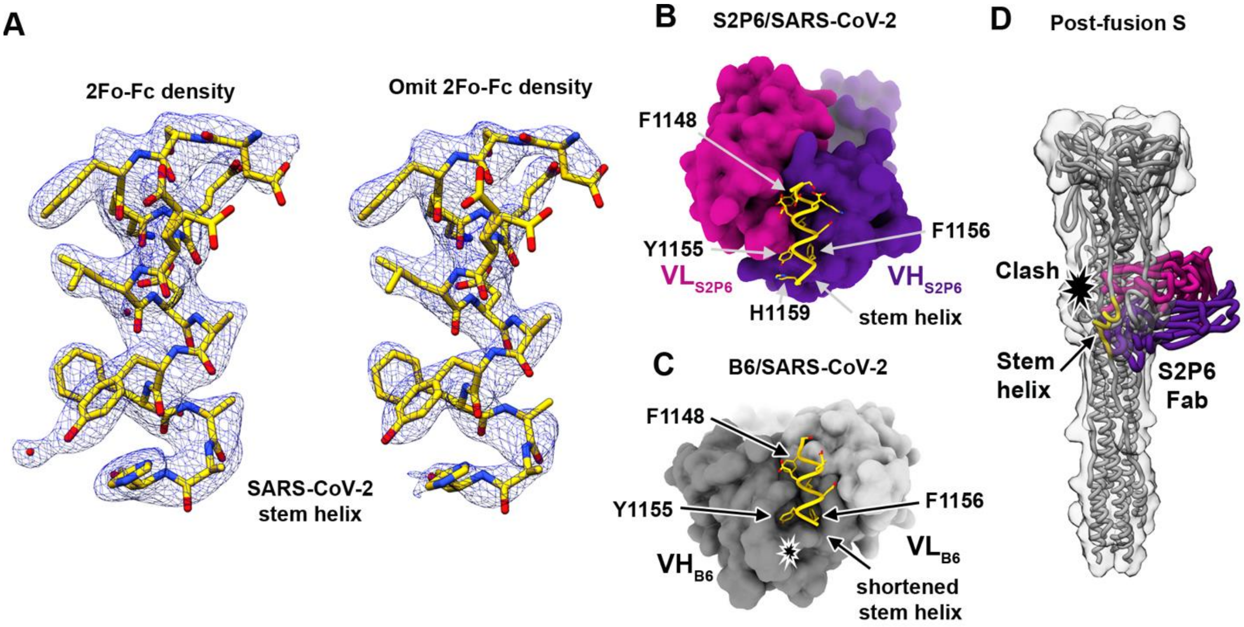

Fig. S4. Comparison of the S2P6 and B6 mAb binding modes. (**A**) Crystal structure of the SARS-CoV-2 stem helix peptide rendered as sticks with the corresponding 2Fo-Fc (left) and 2Fo-Fc omit (annealed, right) maps contoured at 1.0$\boldsymbol{\sigma}$. The S2P6 Fab fragment is not shown for clarity. (**B**) Crystal structure of the S2P6 Fab (surface rendering) in complex with the SARS-CoV-2 S stem helix peptide (yellow ribbon with side chains rendered as sticks). (**C**) Crystal structure of the B6 Fab (surface rendering) in complex with the SARS-CoV-2 S stem helix peptide (yellow ribbon with side chains rendered as sticks). The star indicates the putative clash between B6 CDRH2 and the stem helix C-terminus, likely explaining the latter region is disordered in the B6-bound structure whereas it is resolved in the S2P6-bound structure. (**D**) Superimposition of the S2P6-bound (purple/magenta) SARS-CoV-2 stem helix (yellow) crystal structure onto the SARS-CoV S post-fusion structure (PDB 6M3W) shows that S2P6 binding would be incompatible due to steric hindrance suggesting S2P6 hinders S fusogenic conformational changes. A low-pass filtered surface generated from the SARS-CoV S post-fusion structure is shown as a transparent gray surface to help visualizing clashes.

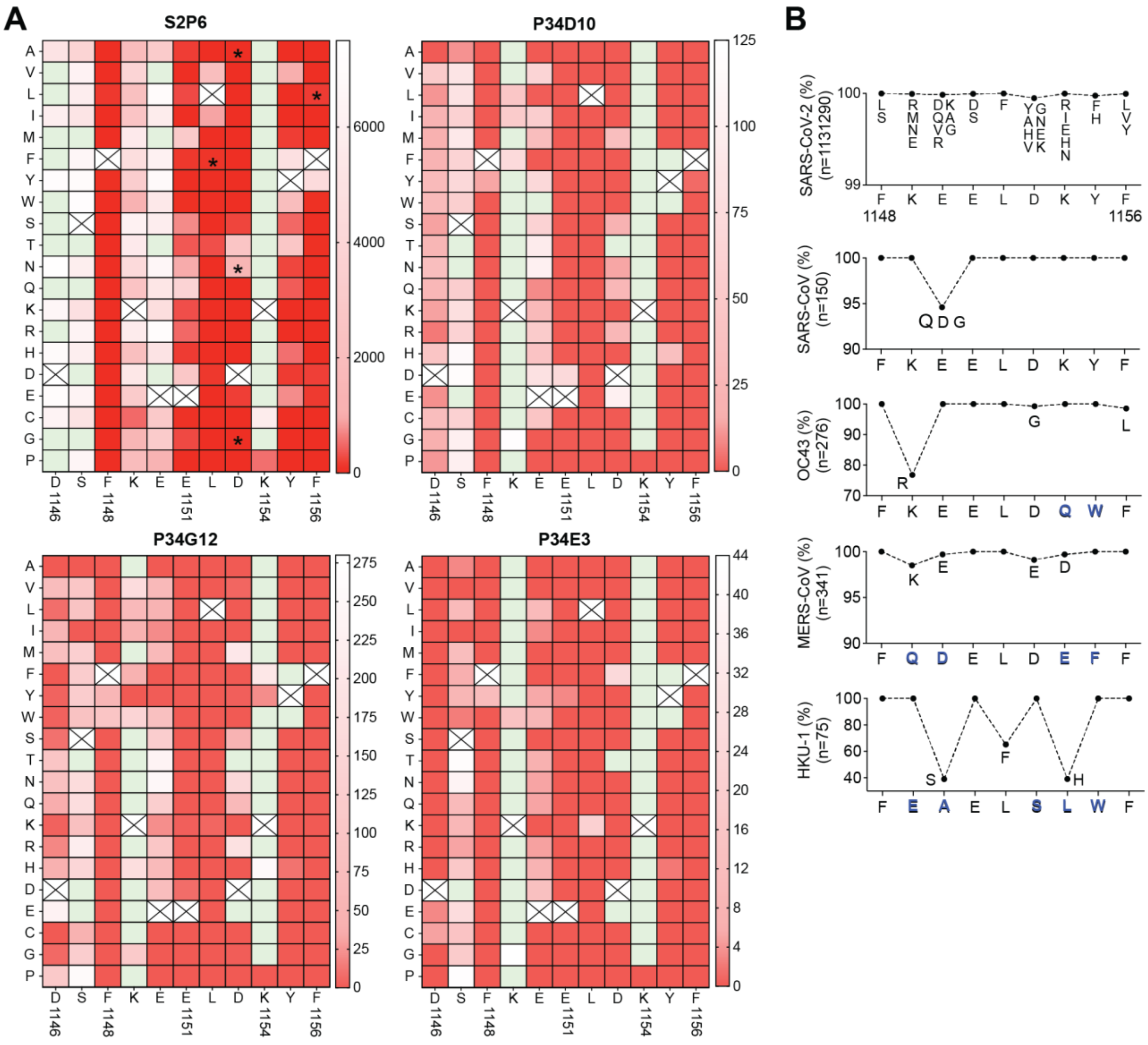

Fig. S5. **Impact of individual SARS-CoV-2 stem helix residue substitution on mAb binding.** (**A**) Heat map showing binding (fluorescence intensity) of S2P6, P34D10, P34G12 and P34E3 to stem helix peptides harboring each possible amino acid substitution. White to red gradient indicates the magnitude of binding attenuation as compared to the native residue shown as a crossed square. Green squares indicate substitutions enhancing binding as compared to the native residue. Asterisks highlight viral escape substitutions identified in vitro for S2P6. (**B**) Epitope conservation among β-coronavirus spike sequences with human and animal hosts retrieved from GISAID. The consensus sequence is reported on x axis and predominant substitutions are indicated by a blue letter.

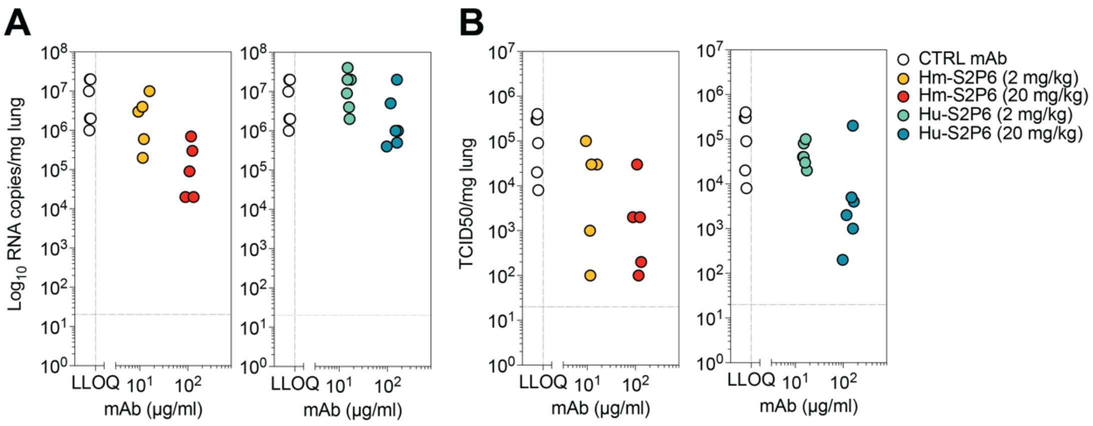

Fig. S6. Relationships between serum S2P6 titers and viral burden. (A-B) Viral RNA copies (A) and replicating virus titers (B) in the lung of Syrian hamsters 4 days post infection with a Wuhan-1 related SARS-CoV-2 isolate plotted as a function of the serum concentration before infection (day 0) of S2P6 harboring a hamster (Hm-S2P6) or a human (Hu-S2P6) constant region.

A

Convalescent donor demographics		
Participants		72
Sex	Female	33
	Male	36
	N/A	3
Age	Average	50
	Range	18-77
Days after symptom onset		
	Range	13-105
Hospitalized		15
	Clinica Luganese Moncucco	3
	Luigi Sacco Hospital	12
Symptomatic		53
	Clinica Luganese Moncucco	7
	Swiss volunteers	20
	US - San Francisco	26
Asymptomatic		4
	Swiss volunteers	2
	US - San Francisco	2

Convalescent donor demographics		
Participants		21
Sex	Female	12
	Male	9
	N/A	0
Age	Average	41
	Range	21-54
Days after PCR positive test		
	Range	207-254
Symptomatic		10
	Ente Ospedaliero Cantonale	
Asymptomatic		11
	Ente Ospedaliero Cantonale	

B

Vaccinated donor demographics		
Participants		46
Sex	Female	18
	Male	28
Age	Average	70
	Range	28-91
SARS-CoV-2	Naïve	37
	Immune	9
Vaccine	Dose 1	3
	Dose 2	43
Cohort	Clinica Luganese Moncucco	17
	Ente Ospedaliero Cantonale	28
	Swiss volunteers	1

Fig. S7. Patient demographics. (**A**) Summary of convalescent patient demographics from which plasma (left table) or memory B cell repertoire (right table) have been analyzed. (**B**) Summary of vaccinated patient demographics from which memory B cell repertoire have been analyzed.

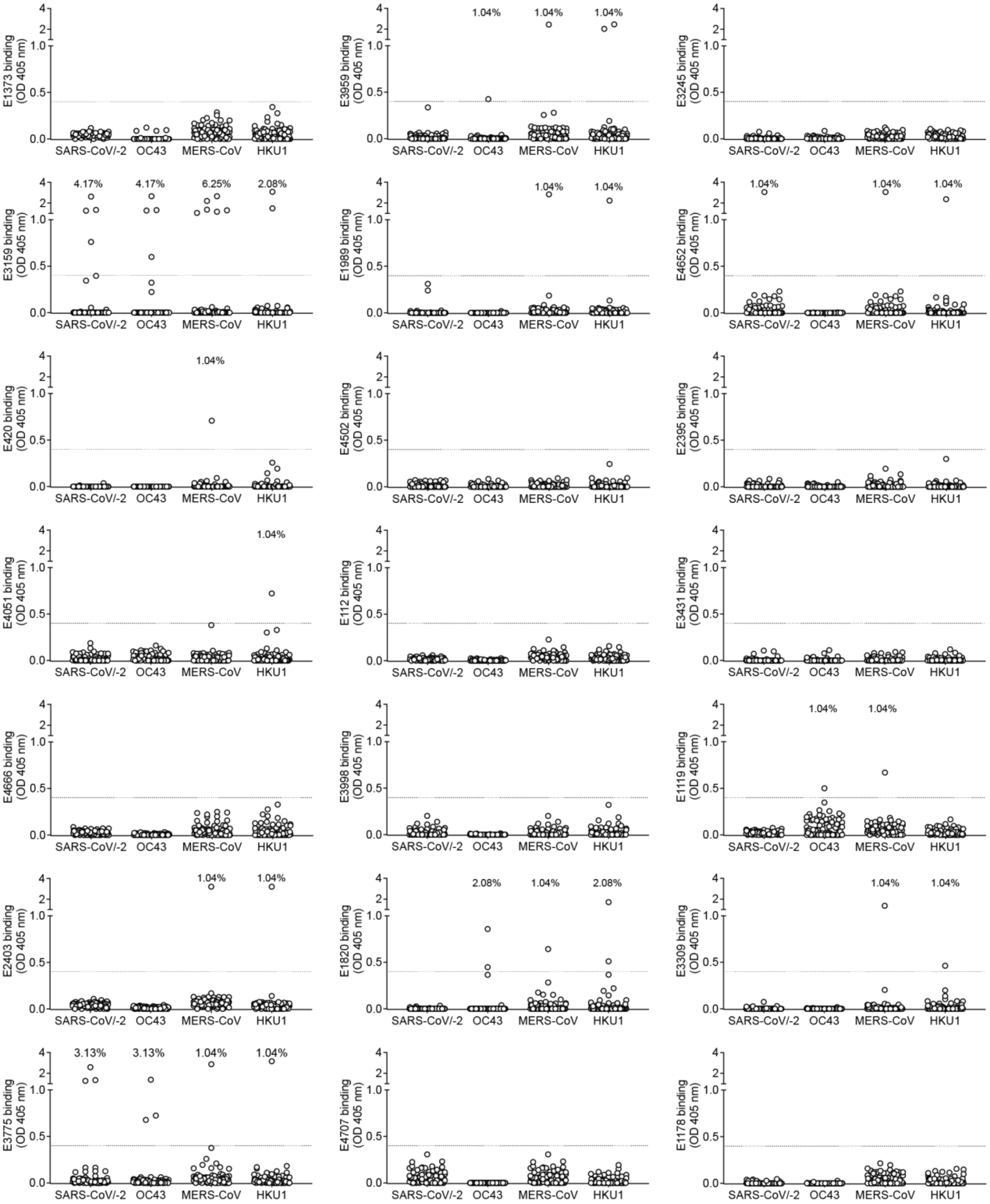

Fig. S8. **Binding of memory B cell culture supernatant from COVID-19 convalescent individuals to β-coronavirus stem helix peptides.** ELISA cut-off (OD=0.4) is indicated by a dotted line and frequencies of positive cultures are reported for each antigen.

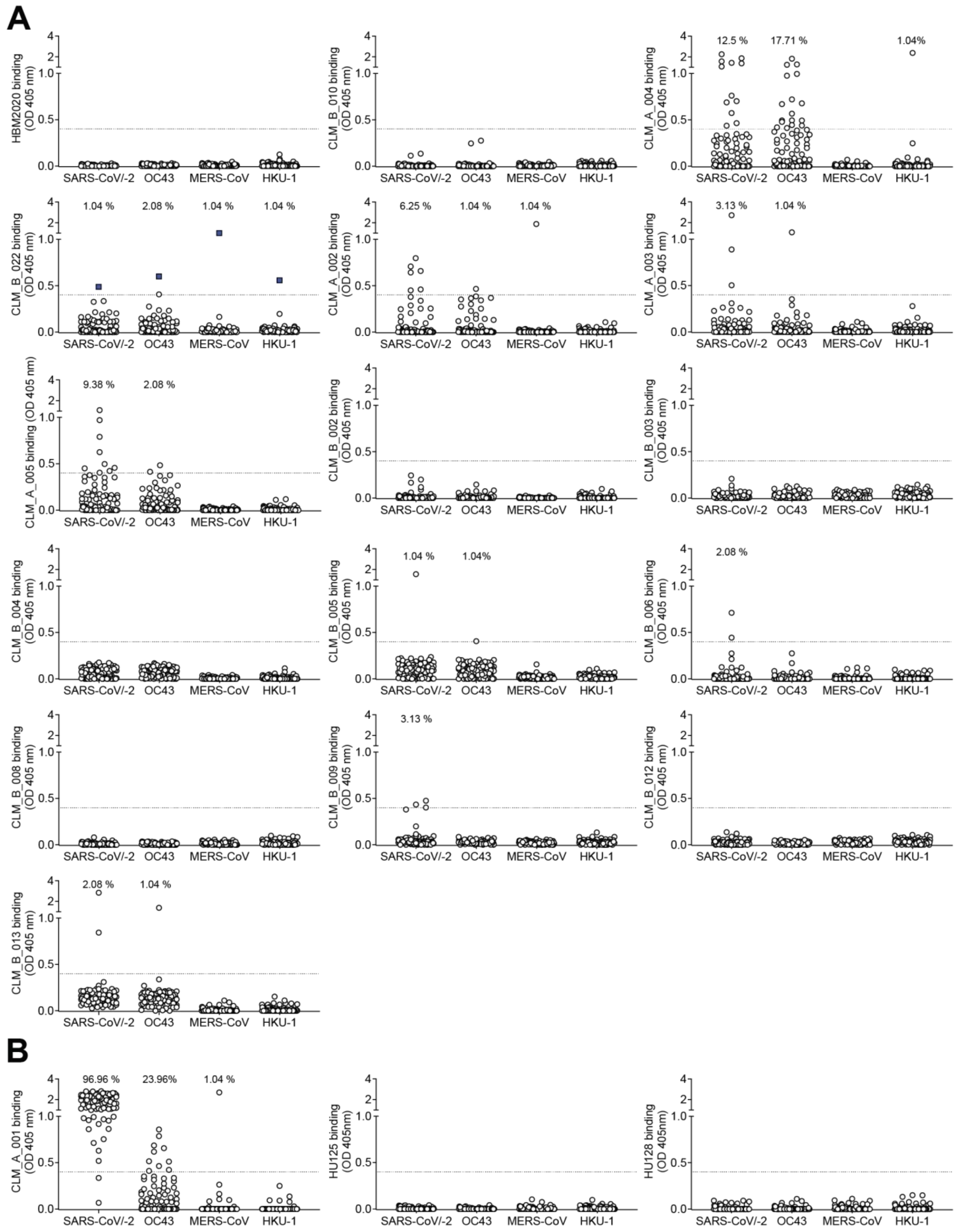

Fig. S9. **Binding of memory B cell culture supernatant from COVID-19 vaccinees to β-coronavirus stem helix peptides.** (**A**) ELISA cut-off (OD=0.4) is indicated by a dotted line and

frequencies of positive cultures are reported for each antigen. (**B**) A previously infected individual who received a first (mRNA) vaccine dose showed high binding responses to SARS-CoV-2 (left). Analysis of two pre-pandemic individuals is shown for comparison (middle and right).

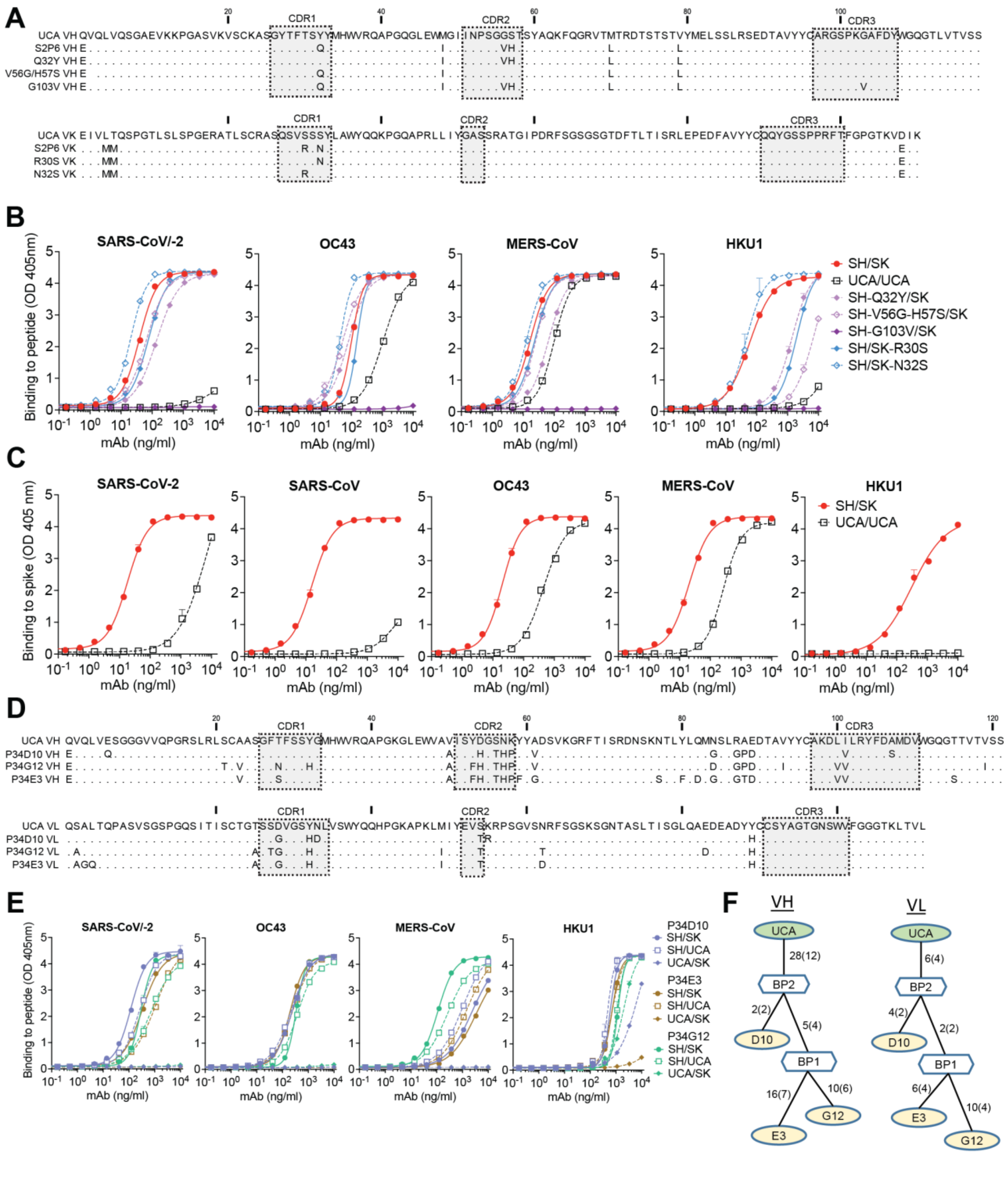

Fig. S10. Analysis of mAbs sequences. (**A**) Alignment of the amino acid sequences of S2P6 UCA and mature VH and VL (top). The CDRs regions are highlighted in gray. (**B**) Binding of S2P6 harboring mature SH or SK with specific germline reverted CDR residues to stem helix peptides. (**C**) Binding of mature (SH/SK) or germline reverted (UCA/UCA) S2P6 to immobilized prefusion-stabilized spike glycoprotein ectodomain trimers. (**D**) Alignment of the amino acid sequences of P34D10, P34G12 and P34E3 UCA and mature VH and VL. (**E**) Binding of mAbs comprising mature heavy chain paired with germline reverted light chain

(SH/UCA) and germline reverted heavy chain paired with mature light chain (UCA/SK) (bottom). (**F**) Ontology trees of VH and VL genes of P34D10, P34G12 and P34E3 shown using AncesTree (*86*). The number of nucleotide and amino acid (in parentheses) mutations from the unmutated common ancestor (UCA) or branch points (BPs) to their descendants are shown.

Supplementary Information

Table S1: CryoEM data collection and refinement statistics.

	S2P6/SARS-CoV-2 S<br>EMD-XXX
**Data collection**	
Magnification	105,000
Voltage (kV)	300
Total exposure (e^{-}/Å^{2})	60
Defocus range (µm)	-0.1 to -5
Pixel size (Å)	0.4215
**Data processing**	
Pixel size (Å)	1.201
Initial particle stack	1,092,473
Final particle stack	81,519
Map resolution (0.143 FSC threshold) (Å)	4.2
Map B-factor	-102.2
Symmetry	C1

Table S2: X-ray crystallography data collection and refinement statistics.

	S2P6/SARS-CoV-2
**Data collection**	
Space group	$P6_522$
Cell dimensions	
a, *b*, *c* (Å)	92.64, 92.64, 223.41
α, β, γ (°)	90.00, 90.00, 120.00
Resolution (Å)	45.84-2.67 (2.77-2.67) *
R_{merge}	0.05 (0.76)
$I / \sigma I$	9.8 (0.83)
Completeness (%)	97.1 (84.9)
Redundancy	2.0 (2.0)
**Refinement**	
Resolution (Å)	45.84-2.67
No. reflections	16,392
R_{work} / R_{free}	0.2349/0.2733
No. atoms	
Protein	3,038
Ligand/ion	20
Water	69
B-factors	
Protein	87.9
Ligand/ion	79.84
Water	65.08
R.m.s. deviations	
Bond lengths (Å)	0.003
Bond angles (°)	0.54

References and Notes

1. M. A. Tortorici, D. Veesler, Structural insights into coronavirus entry. *Adv. Virus Res.* **105**, 93–116 (2019). doi:10.1016/bs.aivir.2019.08.002 Medline

2. A. C. Walls, Y. J. Park, M. A. Tortorici, A. Wall, A. T. McGuire, D. Veesler, Structure, Function, and Antigenicity of the SARS-CoV-2 Spike Glycoprotein. *Cell* **181**, 281–292.e6 (2020). doi:10.1016/j.cell.2020.02.058 Medline

3. D. Wrapp, N. Wang, K. S. Corbett, J. A. Goldsmith, C. L. Hsieh, O. Abiona, B. S. Graham, J. S. McLellan, Cryo-EM structure of the 2019-nCoV spike in the prefusion conformation. *Science* **367**, 1260–1263 (2020). doi:10.1126/science.abb2507 Medline

4. E. C. Thomson, L. E. Rosen, J. G. Shepherd, R. Spreafico, A. da Silva Filipe, J. A. Wojcechowskyj, C. Davis, L. Piccoli, D. J. Pascall, J. Dillen, S. Lytras, N. Czudnochowski, R. Shah, M. Meury, N. Jesudason, A. De Marco, K. Li, J. Bassi, A. O'Toole, D. Pinto, R. M. Colquhoun, K. Culap, B. Jackson, F. Zatta, A. Rambaut, S. Jaconi, V. B. Sreenu, J. Nix, I. Zhang, R. F. Jarrett, W. G. Glass, M. Beltramello, K. Nomikou, M. Pizzuto, L. Tong, E. Cameroni, T. I. Croll, N. Johnson, J. Di Iulio, A. Wickenhagen, A. Ceschi, A. M. Harbison, D. Mair, P. Ferrari, K. Smollett, F. Sallusto, S. Carmichael, C. Garzoni, J. Nichols, M. Galli, J. Hughes, A. Riva, A. Ho, M. Schiuma, M. G. Semple, P. J. M. Openshaw, E. Fadda, J. K. Baillie, J. D. Chodera, ISARIC4C Investigators, COVID-19 Genomics UK (COG-UK) Consortium, S. J. Rihn, S. J. Lycett, H. W. Virgin, A. Telenti, D. Corti, D. L. Robertson, G. Snell, Circulating SARS-CoV-2 spike N439K variants maintain fitness while evading antibody-mediated immunity. *Cell* **184**, 1171–1187.e20 (2021). doi:10.1016/j.cell.2021.01.037 Medline

5. M. McCallum, A. De Marco, F. A. Lempp, M. A. Tortorici, D. Pinto, A. C. Walls, M. Beltramello, A. Chen, Z. Liu, F. Zatta, S. Zepeda, J. di Iulio, J. E. Bowen, M. Montiel-Ruiz, J. Zhou, L. E. Rosen, S. Bianchi, B. Guarino, C. S. Fregni, R. Abdelnabi, S.-Y. C. Foo, P. W. Rothlauf, L.-M. Bloyet, F. Benigni, E. Cameroni, J. Neyts, A. Riva, G. Snell, A. Telenti, S. P. J. Whelan, H. W. Virgin, D. Corti, M. S. Pizzuto, D. Veesler, N-terminal domain antigenic mapping reveals a site of vulnerability for SARS-CoV-2. *Cell* **184**, 2332–2347.e16 (2021). doi:10.1016/j.cell.2021.03.028 Medline

6. K. R. McCarthy, L. J. Rennick, S. Nambulli, L. R. Robinson-McCarthy, W. G. Bain, G. Haidar, W. P. Duprex, Recurrent deletions in the SARS-CoV-2 spike glycoprotein drive antibody escape. *Science* **371**, 1139–1142 (2021). doi:10.1126/science.abf6950 Medline

7. B. Choi, M. C. Choudhary, J. Regan, J. A. Sparks, R. F. Padera, X. Qiu, I. H. Solomon, H. H. Kuo, J. Boucau, K. Bowman, U. D. Adhikari, M. L. Winkler, A. A. Mueller, T. Y. Hsu, M. Desjardins, L. R. Baden, B. T. Chan, B. D. Walker, M. Lichterfeld, M. Brigl, D. S. Kwon, S. Kanjilal, E. T. Richardson, A. H. Jonsson, G. Alter, A. K. Barczak, W. P. Hanage, X. G. Yu, G. D. Gaiha, M. S. Seaman, M. Cernadas, J. Z. Li, Persistence and Evolution of SARS-CoV-2 in an Immunocompromised Host. *N. Engl. J. Med.* **383**, 2291–2293 (2020). doi:10.1056/NEJMc2031364 Medline

8. Z. Liu, L. A. VanBlargan, L.-M. Bloyet, P. W. Rothlauf, R. E. Chen, S. Stumpf, H. Zhao, J. M. Errico, E. S. Theel, M. J. Liebeskind, B. Alford, W. J. Buchser, A. H. Ellebedy, D. H. Fremont, M. S. Diamond, S. P. J. Whelan, Identification of SARS-CoV-2 spike mutations

that attenuate monoclonal and serum antibody neutralization. *Cell Host Microbe* **29**, 477–488.e4 (2021). doi:10.1016/j.chom.2021.01.014 Medline

9. Y. Weisblum, F. Schmidt, F. Zhang, J. DaSilva, D. Poston, J. C. C. Lorenzi, F. Muecksch, M. Rutkowska, H.-H. Hoffmann, E. Michailidis, C. Gaebler, M. Agudelo, A. Cho, Z. Wang, A. Gazumyan, M. Cipolla, L. Luchsinger, C. D. Hillyer, M. Caskey, D. F. Robbiani, C. M. Rice, M. C. Nussenzweig, T. Hatziioannou, P. D. Bieniasz, Escape from neutralizing antibodies by SARS-CoV-2 spike protein variants. *eLife* **9**, e61312 (2020). doi:10.7554/eLife.61312 Medline

10. M. McCallum, J. Bassi, A. De Marco, A. Chen, A. C. Walls, J. Di Iulio, M. A. Tortorici, M.-J. Navarro, C. Silacci-Fregni, C. Saliba, K. R. Sprouse, M. Agostini, D. Pinto, K. Culap, S. Bianchi, S. Jaconi, E. Cameroni, J. E. Bowen, S. W. Tilles, M. S. Pizzuto, S. B. Guastalla, G. Bona, A. F. Pellanda, C. Garzoni, W. C. Van Voorhis, L. E. Rosen, G. Snell, A. Telenti, H. W. Virgin, L. Piccoli, D. Corti, D. Veesler, SARS-CoV-2 immune evasion by the B.1.427/B.1.429 variant of concern. *Science* **373**, 648–654 (2021). doi:10.1126/science.abi7994 Medline

11. A. C. Walls, X. Xiong, Y. J. Park, M. A. Tortorici, J. Snijder, J. Quispe, E. Cameroni, R. Gopal, M. Dai, A. Lanzavecchia, M. Zambon, F. A. Rey, D. Corti, D. Veesler, Unexpected Receptor Functional Mimicry Elucidates Activation of Coronavirus Fusion. *Cell* **176**, 1026–1039.e15 (2019). doi:10.1016/j.cell.2018.12.028 Medline

12. Y. Watanabe, J. D. Allen, D. Wrapp, J. S. McLellan, M. Crispin, Site-specific glycan analysis of the SARS-CoV-2 spike. *Science* **369**, 330–333 (2020). doi:10.1126/science.abb9983 Medline

13. Y. Yang, C. Liu, L. Du, S. Jiang, Z. Shi, R. S. Baric, F. Li, Two Mutations Were Critical for Bat-to-Human Transmission of Middle East Respiratory Syndrome Coronavirus. *J. Virol.* **89**, 9119–9123 (2015). doi:10.1128/JVI.01279-15 Medline

14. M. M. Sauer, M. A. Tortorici, Y.-J. Park, A. C. Walls, L. Homad, O. Acton, J. Bowen, C. Wang, X. Xiong, W. de van der Schueren, J. Quispe, B. G. Hoffstrom, B.-J. Bosch, A. T. McGuire, D. Veesler, Structural basis for broad coronavirus neutralization. bioRxiv 2020.12.29.424482 [Preprint] (2021). https://doi.org/10.1101/2020.12.29.424482.

15. C. Wang, R. van Haperen, J. Gutiérrez-Álvarez, W. Li, N. M. A. Okba, I. Albulescu, I. Widjaja, B. van Dieren, R. Fernandez-Delgado, I. Sola, D. L. Hurdiss, O. Daramola, F. Grosveld, F. J. M. van Kuppeveld, B. L. Haagmans, L. Enjuanes, D. Drabek, B.-J. Bosch, A conserved immunogenic and vulnerable site on the coronavirus spike protein delineated by cross-reactive monoclonal antibodies. *Nat. Commun.* **12**, 1715 (2021). doi:10.1038/s41467-021-21968-w Medline

16. G. Song, W.-t. He, S. Callaghan, F. Anzanello, D. Huang, J. Ricketts, J. L. Torres, N. Beutler, L. Peng, S. Vargas, J. Cassell, M. Parren, L. Yang, C. Ignacio, D. M. Smith, J. E. Voss, D. Nemazee, A. B. Ward, T. Rogers, D. R. Burton, R. Andrabi, Cross-reactive serum and memory B-cell responses to spike protein in SARS-CoV-2 and endemic coronavirus infection. *Nat. Commun.* **12**, 2938 (2021). doi:10.1038/s41467-021-23074-3 Medline

17. N. L. Kallewaard, D. Corti, P. J. Collins, U. Neu, J. M. McAuliffe, E. Benjamin, L. Wachter-Rosati, F. J. Palmer-Hill, A. Q. Yuan, P. A. Walker, M. K. Vorlaender, S. Bianchi, B.

Guarino, A. De Marco, F. Vanzetta, G. Agatic, M. Foglierini, D. Pinna, B. Fernandez-Rodriguez, A. Fruehwirth, C. Silacci, R. W. Ogrodowicz, S. R. Martin, F. Sallusto, J. A. Suzich, A. Lanzavecchia, Q. Zhu, S. J. Gamblin, J. J. Skehel, Structure and Function Analysis of an Antibody Recognizing All Influenza A Subtypes. *Cell* **166**, 596–608 (2016). doi:10.1016/j.cell.2016.05.073 Medline

18. B. F. Haynes, D. R. Burton, J. R. Mascola, Multiple roles for HIV broadly neutralizing antibodies. *Sci. Transl. Med.* **11**, eaaz2686 (2019). doi:10.1126/scitranslmed.aaz2686 Medline

19. Z. Wang, F. Schmidt, Y. Weisblum, F. Muecksch, C. O. Barnes, S. Finkin, D. Schaefer-Babajew, M. Cipolla, C. Gaebler, J. A. Lieberman, T. Y. Oliveira, Z. Yang, M. E. Abernathy, K. E. Huey-Tubman, A. Hurley, M. Turroja, K. A. West, K. Gordon, K. G. Millard, V. Ramos, J. Da Silva, J. Xu, R. A. Colbert, R. Patel, J. Dizon, C. Unson-O'Brien, I. Shimeliovich, A. Gazumyan, M. Caskey, P. J. Bjorkman, R. Casellas, T. Hatziioannou, P. D. Bieniasz, M. C. Nussenzweig, mRNA vaccine-elicited antibodies to SARS-CoV-2 and circulating variants. *Nature* **592**, 616–622 (2021). doi:10.1038/s41586-021-03324-6 Medline

20. L. Piccoli, Y. J. Park, M. A. Tortorici, N. Czudnochowski, A. C. Walls, M. Beltramello, C. Silacci-Fregni, D. Pinto, L. E. Rosen, J. E. Bowen, O. J. Acton, S. Jaconi, B. Guarino, A. Minola, F. Zatta, N. Sprugasci, J. Bassi, A. Peter, A. De Marco, J. C. Nix, F. Mele, S. Jovic, B. F. Rodriguez, S. V. Gupta, F. Jin, G. Piumatti, G. Lo Presti, A. F. Pellanda, M. Biggiogero, M. Tarkowski, M. S. Pizzuto, E. Cameroni, C. Havenar-Daughton, M. Smithey, D. Hong, V. Lepori, E. Albanese, A. Ceschi, E. Bernasconi, L. Elzi, P. Ferrari, C. Garzoni, A. Riva, G. Snell, F. Sallusto, K. Fink, H. W. Virgin, A. Lanzavecchia, D. Corti, D. Veesler, Mapping Neutralizing and Immunodominant Sites on the SARS-CoV-2 Spike Receptor-Binding Domain by Structure-Guided High-Resolution Serology. *Cell* **183**, 1024–1042.e21 (2020). doi:10.1016/j.cell.2020.09.037 Medline

21. M. A. Tortorici, M. Beltramello, F. A. Lempp, D. Pinto, H. V. Dang, L. E. Rosen, M. McCallum, J. Bowen, A. Minola, S. Jaconi, F. Zatta, A. De Marco, B. Guarino, S. Bianchi, E. J. Lauron, H. Tucker, J. Zhou, A. Peter, C. Havenar-Daughton, J. A. Wojcechowskyj, J. B. Case, R. E. Chen, H. Kaiser, M. Montiel-Ruiz, M. Meury, N. Czudnochowski, R. Spreafico, J. Dillen, C. Ng, N. Sprugasci, K. Culap, F. Benigni, R. Abdelnabi, S. C. Foo, M. A. Schmid, E. Cameroni, A. Riva, A. Gabrieli, M. Galli, M. S. Pizzuto, J. Neyts, M. S. Diamond, H. W. Virgin, G. Snell, D. Corti, K. Fink, D. Veesler, Ultrapotent human antibodies protect against SARS-CoV-2 challenge via multiple mechanisms. *Science* **370**, 950–957 (2020). doi:10.1126/science.abe3354 Medline

22. M. Hoffmann, K. Mösbauer, H. Hofmann-Winkler, A. Kaul, H. Kleine-Weber, N. Krüger, N. C. Gassen, M. A. Müller, C. Drosten, S. Pöhlmann, Chloroquine does not inhibit infection of human lung cells with SARS-CoV-2. *Nature* **585**, 588–590 (2020). doi:10.1038/s41586-020-2575-3 Medline

23. M. Hoffmann, H. Kleine-Weber, S. Schroeder, N. Krüger, T. Herrler, S. Erichsen, T. S. Schiergens, G. Herrler, N. H. Wu, A. Nitsche, M. A. Müller, C. Drosten, S. Pöhlmann, SARS-CoV-2 Cell Entry Depends on ACE2 and TMPRSS2 and Is Blocked by a Clinically Proven Protease Inhibitor. *Cell* **181**, 271–280.e8 (2020). doi:10.1016/j.cell.2020.02.052 Medline

24. M. Hoffmann, H. Kleine-Weber, S. Pöhlmann, A Multibasic Cleavage Site in the Spike Protein of SARS-CoV-2 Is Essential for Infection of Human Lung Cells. *Mol. Cell* **78**, 779–784.e5 (2020). doi:10.1016/j.molcel.2020.04.022 Medline

25. Y. Kaname, H. Tani, C. Kataoka, M. Shiokawa, S. Taguwa, T. Abe, K. Moriishi, T. Kinoshita, Y. Matsuura, Acquisition of complement resistance through incorporation of CD55/decay-accelerating factor into viral particles bearing baculovirus GP64. *J. Virol.* **84**, 3210–3219 (2010). doi:10.1128/JVI.02519-09 Medline

26. J. B. Case, P. W. Rothlauf, R. E. Chen, Z. Liu, H. Zhao, A. S. Kim, L. M. Bloyet, Q. Zeng, S. Tahan, L. Droit, M. X. G. Ilagan, M. A. Tartell, G. Amarasinghe, J. P. Henderson, S. Miersch, M. Ustav, S. Sidhu, H. W. Virgin, D. Wang, S. Ding, D. Corti, E. S. Theel, D. H. Fremont, M. S. Diamond, S. P. J. Whelan, Neutralizing Antibody and Soluble ACE2 Inhibition of a Replication-Competent VSV-SARS-CoV-2 and a Clinical Isolate of SARS-CoV-2. *Cell Host Microbe* **28**, 475–485.e5 (2020). doi:10.1016/j.chom.2020.06.021 Medline

27. M. Gui, W. Song, H. Zhou, J. Xu, S. Chen, Y. Xiang, X. Wang, Cryo-electron microscopy structures of the SARS-CoV spike glycoprotein reveal a prerequisite conformational state for receptor binding. *Cell Res.* **27**, 119–129 (2017). doi:10.1038/cr.2016.152 Medline

28. R. N. Kirchdoerfer, N. Wang, J. Pallesen, D. Wrapp, H. L. Turner, C. A. Cottrell, K. S. Corbett, B. S. Graham, J. S. McLellan, A. B. Ward, Stabilized coronavirus spikes are resistant to conformational changes induced by receptor recognition or proteolysis. *Sci. Rep.* **8**, 15701 (2018). doi:10.1038/s41598-018-34171-7 Medline

29. Y. Yuan, D. Cao, Y. Zhang, J. Ma, J. Qi, Q. Wang, G. Lu, Y. Wu, J. Yan, Y. Shi, X. Zhang, G. F. Gao, Cryo-EM structures of MERS-CoV and SARS-CoV spike glycoproteins reveal the dynamic receptor binding domains. *Nat. Commun.* **8**, 15092 (2017). doi:10.1038/ncomms15092 Medline

30. A. C. Walls, M. A. Tortorici, J. Snijder, X. Xiong, B. J. Bosch, F. A. Rey, D. Veesler, Tectonic conformational changes of a coronavirus spike glycoprotein promote membrane fusion. *Proc. Natl. Acad. Sci. U.S.A.* **114**, 11157–11162 (2017). doi:10.1073/pnas.1708727114 Medline

31. Y. Cai, J. Zhang, T. Xiao, H. Peng, S. M. Sterling, R. M. Walsh Jr., S. Rawson, S. Rits-Volloch, B. Chen, Distinct conformational states of SARS-CoV-2 spike protein. *Science* **369**, 1586–1592 (2020). doi:10.1126/science.abd4251 Medline

32. X. Fan, D. Cao, L. Kong, X. Zhang, Cryo-EM analysis of the post-fusion structure of the SARS-CoV spike glycoprotein. *Nat. Commun.* **11**, 3618 (2020). doi:10.1038/s41467-020-17371-6 Medline

33. F. A. Lempp, L. Soriaga, M. Montiel-Ruiz, F. Benigni, J. Noack, Y.-J. Park, S. Bianchi, A. C. Walls, J. E. Bowen, J. Zhou, H. Kaiser, M. Agostini, M. Meury, E. Dellota Jr., S. Jaconi, E. Cameroni, H. W. Virgin, A. Lanzavecchia, D. Veesler, L. Purcell, A. Telenti, D. Corti, Membrane lectins enhance SARS-CoV-2 infection and influence the neutralizing activity of different classes of antibodies. bioRxiv 2021.04.03.438258 [Preprint] (2021). https://doi.org/10.1101/2021.04.03.438258.

34. A. Schäfer, F. Muecksch, J. C. C. Lorenzi, S. R. Leist, M. Cipolla, S. Bournazos, F. Schmidt, R. M. Maison, A. Gazumyan, D. R. Martinez, R. S. Baric, D. F. Robbiani, T. Hatziioannou, J. V. Ravetch, P. D. Bieniasz, R. A. Bowen, M. C. Nussenzweig, T. P. Sheahan, Antibody potency, effector function, and combinations in protection and therapy for SARS-CoV-2 infection in vivo. *J. Exp. Med.* **218**, e20201993 (2021). doi:10.1084/jem.20201993 Medline

35. S. Bournazos, T. T. Wang, J. V. Ravetch, The Role and Function of Fcγ Receptors on Myeloid Cells. *Microbiol. Spectr.* **4**, 4.6.20 (2016). doi:10.1128/microbiolspec.MCHD-0045-2016 Medline

36. S. Bournazos, D. Corti, H. W. Virgin, J. V. Ravetch, Fc-optimized antibodies elicit CD8 immunity to viral respiratory infection. *Nature* **588**, 485–490 (2020). doi:10.1038/s41586-020-2838-z Medline

37. E. S. Winkler, P. Gilchuk, J. Yu, A. L. Bailey, R. E. Chen, Z. Chong, S. J. Zost, H. Jang, Y. Huang, J. D. Allen, J. B. Case, R. E. Sutton, R. H. Carnahan, T. L. Darling, A. C. M. Boon, M. Mack, R. D. Head, T. M. Ross, J. E. Crowe Jr., M. S. Diamond, Human neutralizing antibodies against SARS-CoV-2 require intact Fc effector functions for optimal therapeutic protection. *Cell* **184**, 1804–1820.E16 (2021). doi:10.1016/j.cell.2021.02.026 Medline

38. D. Pinto, Y. J. Park, M. Beltramello, A. C. Walls, M. A. Tortorici, S. Bianchi, S. Jaconi, K. Culap, F. Zatta, A. De Marco, A. Peter, B. Guarino, R. Spreafico, E. Cameroni, J. B. Case, R. E. Chen, C. Havenar-Daughton, G. Snell, A. Telenti, H. W. Virgin, A. Lanzavecchia, M. S. Diamond, K. Fink, D. Veesler, D. Corti, Cross-neutralization of SARS-CoV-2 by a human monoclonal SARS-CoV antibody. *Nature* **583**, 290–295 (2020). doi:10.1038/s41586-020-2349-y Medline

39. R. Boudewijns, H. J. Thibaut, S. J. F. Kaptein, R. Li, V. Vergote, L. Seldeslachts, J. Van Weyenbergh, C. De Keyzer, L. Bervoets, S. Sharma, L. Liesenborghs, J. Ma, S. Jansen, D. Van Looveren, T. Vercruysse, X. Wang, D. Jochmans, E. Martens, K. Roose, D. De Vlieger, B. Schepens, T. Van Buyten, S. Jacobs, Y. Liu, J. Martí-Carreras, B. Vanmechelen, T. Wawina-Bokalanga, L. Delang, J. Rocha-Pereira, L. Coelmont, W. Chiu, P. Leyssen, E. Heylen, D. Schols, L. Wang, L. Close, J. Matthijnssens, M. Van Ranst, V. Compernolle, G. Schramm, K. Van Laere, X. Saelens, N. Callewaert, G. Opdenakker, P. Maes, B. Weynand, C. Cawthorne, G. Vande Velde, Z. Wang, J. Neyts, K. Dallmeier, STAT2 signaling restricts viral dissemination but drives severe pneumonia in SARS-CoV-2 infected hamsters. *Nat. Commun.* **11**, 5838 (2020). doi:10.1038/s41467-020-19684-y Medline

40. D. Pinna, D. Corti, D. Jarrossay, F. Sallusto, A. Lanzavecchia, Clonal dissection of the human memory B-cell repertoire following infection and vaccination. *Eur. J. Immunol.* **39**, 1260–1270 (2009). doi:10.1002/eji.200839129 Medline

41. C. Daniel, R. Anderson, M. J. Buchmeier, J. O. Fleming, W. J. Spaan, H. Wege, P. J. Talbot, Identification of an immunodominant linear neutralization domain on the S2 portion of the murine coronavirus spike glycoprotein and evidence that it forms part of complex tridimensional structure. *J. Virol.* **67**, 1185–1194 (1993). doi:10.1128/jvi.67.3.1185-1194.1993 Medline

42. H. Zhang, G. Wang, J. Li, Y. Nie, X. Shi, G. Lian, W. Wang, X. Yin, Y. Zhao, X. Qu, M. Ding, H. Deng, Identification of an antigenic determinant on the S2 domain of the severe acute respiratory syndrome coronavirus spike glycoprotein capable of inducing neutralizing antibodies. *J. Virol.* **78**, 6938–6945 (2004). doi:10.1128/JVI.78.13.6938-6945.2004 Medline

43. C. M. Poh, G. Carissimo, B. Wang, S. N. Amrun, C. Y. Lee, R. S. Chee, S. W. Fong, N. K. Yeo, W. H. Lee, A. Torres-Ruesta, Y. S. Leo, M. I. Chen, S. Y. Tan, L. Y. A. Chai, S. Kalimuddin, S. S. G. Kheng, S. Y. Thien, B. E. Young, D. C. Lye, B. J. Hanson, C. I. Wang, L. Renia, L. F. P. Ng, Two linear epitopes on the SARS-CoV-2 spike protein that elicit neutralising antibodies in COVID-19 patients. *Nat. Commun.* **11**, 2806 (2020). doi:10.1038/s41467-020-16638-2 Medline

44. H. A. Elshabrawy, M. M. Coughlin, S. C. Baker, B. S. Prabhakar, Human monoclonal antibodies against highly conserved HR1 and HR2 domains of the SARS-CoV spike protein are more broadly neutralizing. *PLOS ONE* **7**, e50366 (2012). doi:10.1371/journal.pone.0050366 Medline

45. Z. Zheng, V. M. Monteil, S. Maurer-Stroh, C. W. Yew, C. Leong, N. K. Mohd-Ismail, S. Cheyyatraivendran Arularasu, V. T. K. Chow, R. T. P. Lin, A. Mirazimi, W. Hong, Y. J. Tan, Monoclonal antibodies for the S2 subunit of spike of SARS-CoV-1 cross-react with the newly-emerged SARS-CoV-2. *Euro Surveill.* **25**, 2000291 (2020). doi:10.2807/1560-7917.ES.2020.25.28.2000291 Medline

46. A. C. Walls, M. A. Tortorici, B. J. Bosch, B. Frenz, P. J. M. Rottier, F. DiMaio, F. A. Rey, D. Veesler, Cryo-electron microscopy structure of a coronavirus spike glycoprotein trimer. *Nature* **531**, 114–117 (2016). doi:10.1038/nature16988 Medline

47. P. Zhou, M. Yuan, G. Song, N. Beutler, N. Shaabani, D. Huang, W.-t. He, X. Zhu, S. Callaghan, P. Yong, F. Anzanello, L. Peng, J. Ricketts, M. Parren, E. Garcia, S. A. Rawlings, D. M. Smith, D. Nemazee, J. R. Teijaro, T. F. Rogers, I. A. Wilson, D. R. Burton, R. Andrabi, A protective broadly cross-reactive human antibody defines a conserved site of vulnerability on beta-coronavirus spikes. bioRxiv 2021.03.30.437769 [Preprint] (2021). https://doi.org/10.1101/2021.03.30.437769.

48. D. Corti, J. Voss, S. J. Gamblin, G. Codoni, A. Macagno, D. Jarrossay, S. G. Vachieri, D. Pinna, A. Minola, F. Vanzetta, C. Silacci, B. M. Fernandez-Rodriguez, G. Agatic, S. Bianchi, I. Giacchetto-Sasselli, L. Calder, F. Sallusto, P. Collins, L. F. Haire, N. Temperton, J. P. Langedijk, J. J. Skehel, A. Lanzavecchia, A neutralizing antibody selected from plasma cells that binds to group 1 and group 2 influenza A hemagglutinins. *Science* **333**, 850–856 (2011). doi:10.1126/science.1205669 Medline

49. D. J. DiLillo, G. S. Tan, P. Palese, J. V. Ravetch, Broadly neutralizing hemagglutinin stalk-specific antibodies require FcγR interactions for protection against influenza virus in vivo. *Nat. Med.* **20**, 143–151 (2014). doi:10.1038/nm.3443 Medline

50. D. Corti, A. L. Suguitan Jr., D. Pinna, C. Silacci, B. M. Fernandez-Rodriguez, F. Vanzetta, C. Santos, C. J. Luke, F. J. Torres-Velez, N. J. Temperton, R. A. Weiss, F. Sallusto, K. Subbarao, A. Lanzavecchia, Heterosubtypic neutralizing antibodies are produced by individuals immunized with a seasonal influenza vaccine. *J. Clin. Invest.* **120**, 1663–1673 (2010). doi:10.1172/JCI41902 Medline

51. F. Sesterhenn, C. Yang, J. Bonet, J. T. Cramer, X. Wen, Y. Wang, C. I. Chiang, L. A. Abriata, I. Kucharska, G. Castoro, S. S. Vollers, M. Galloux, E. Dheilly, S. Rosset, P. Corthésy, S. Georgeon, M. Villard, C. A. Richard, D. Descamps, T. Delgado, E. Oricchio, M. A. Rameix-Welti, V. Más, S. Ervin, J. F. Eléouët, S. Riffault, J. T. Bates, J. P. Julien, Y. Li, T. Jardetzky, T. Krey, B. E. Correia, De novo protein design enables the precise induction of RSV-neutralizing antibodies. *Science* **368**, eaay5051 (2020). doi:10.1126/science.aay5051 Medline

52. M. L. Azoitei, B. E. Correia, Y. E. Ban, C. Carrico, O. Kalyuzhniy, L. Chen, A. Schroeter, P. S. Huang, J. S. McLellan, P. D. Kwong, D. Baker, R. K. Strong, W. R. Schief, Computation-guided backbone grafting of a discontinuous motif onto a protein scaffold. *Science* **334**, 373–376 (2011). doi:10.1126/science.1209368 Medline

53. B. E. Correia, J. T. Bates, R. J. Loomis, G. Baneyx, C. Carrico, J. G. Jardine, P. Rupert, C. Correnti, O. Kalyuzhniy, V. Vittal, M. J. Connell, E. Stevens, A. Schroeter, M. Chen, S. Macpherson, A. M. Serra, Y. Adachi, M. A. Holmes, Y. Li, R. E. Klevit, B. S. Graham, R. T. Wyatt, D. Baker, R. K. Strong, J. E. Crowe Jr., P. R. Johnson, W. R. Schief, Proof of principle for epitope-focused vaccine design. *Nature* **507**, 201–206 (2014). doi:10.1038/nature12966 Medline

54. A. C. Walls, B. Fiala, A. Schäfer, S. Wrenn, M. N. Pham, M. Murphy, L. V. Tse, L. Shehata, M. A. O'Connor, C. Chen, M. J. Navarro, M. C. Miranda, D. Pettie, R. Ravichandran, J. C. Kraft, C. Ogohara, A. Palser, S. Chalk, E. C. Lee, K. Guerriero, E. Kepl, C. M. Chow, C. Sydeman, E. A. Hodge, B. Brown, J. T. Fuller, K. H. Dinnon 3rd, L. E. Gralinski, S. R. Leist, K. L. Gully, T. B. Lewis, M. Guttman, H. Y. Chu, K. K. Lee, D. H. Fuller, R. S. Baric, P. Kellam, L. Carter, M. Pepper, T. P. Sheahan, D. Veesler, N. P. King, Elicitation of Potent Neutralizing Antibody Responses by Designed Protein Nanoparticle Vaccines for SARS-CoV-2. *Cell* **183**, 1367–1382.e17 (2020). doi:10.1016/j.cell.2020.10.043 Medline

55. A. C. Walls, M. C. Miranda, M. N. Pham, A. Schäfer, A. Greaney, P. S. Arunachalam, M.-J. Navarro, M. A. Tortorici, K. Rogers, M. A. O'Connor, L. Shireff, D. E. Ferrell, N. Brunette, E. Kepl, J. Bowen, S. K. Zepeda, T. Starr, C.-L. Hsieh, B. Fiala, S. Wrenn, D. Pettie, C. Sydeman, M. Johnson, A. Blackstone, R. Ravichandran, C. Ogohara, L. Carter, S. W. Tilles, R. Rappuoli, D. T. O'Hagan, R. Van Der Most, W. C. Van Voorhis, J. S. McLellan, H. Kleanthous, T. P. Sheahan, D. H. Fuller, F. Villinger, J. Bloom, B. Pulendran, R. Baric, N. King, D. Veesler, Elicitation of broadly protective sarbecovirus immunity by receptor-binding domain nanoparticle vaccines. bioRxiv 2021.03.15.435528 [Preprint] (2021). https://doi.org/10.1101/2021.03.15.435528.

56. S. Boyoglu-Barnum, D. Ellis, R. A. Gillespie, G. B. Hutchinson, Y.-J. Park, S. M. Moin, O. J. Acton, R. Ravichandran, M. Murphy, D. Pettie, N. Matheson, L. Carter, A. Creanga, M. J. Watson, S. Kephart, S. Ataca, J. R. Vaile, G. Ueda, M. C. Crank, L. Stewart, K. K. Lee, M. Guttman, D. Baker, J. R. Mascola, D. Veesler, B. S. Graham, N. P. King, M. Kanekiyo, Quadrivalent influenza nanoparticle vaccines induce broad protection. *Nature* **592**, 623–628 (2021). doi:10.1038/s41586-021-03365-x Medline

57. M. Kanekiyo, C. J. Wei, H. M. Yassine, P. M. McTamney, J. C. Boyington, J. R. Whittle, S. S. Rao, W. P. Kong, L. Wang, G. J. Nabel, Self-assembling influenza nanoparticle

vaccines elicit broadly neutralizing H1N1 antibodies. *Nature* **499**, 102–106 (2013). doi:10.1038/nature12202 Medline

58. M. Kanekiyo, M. G. Joyce, R. A. Gillespie, J. R. Gallagher, S. F. Andrews, H. M. Yassine, A. K. Wheatley, B. E. Fisher, D. R. Ambrozak, A. Creanga, K. Leung, E. S. Yang, S. Boyoglu-Barnum, I. S. Georgiev, Y. Tsybovsky, M. S. Prabhakaran, H. Andersen, W. P. Kong, U. Baxa, K. L. Zephir, J. E. Ledgerwood, R. A. Koup, P. D. Kwong, A. K. Harris, A. B. McDermott, J. R. Mascola, B. S. Graham, Mosaic nanoparticle display of diverse influenza virus hemagglutinins elicits broad B cell responses. *Nat. Immunol.* **20**, 362–372 (2019). doi:10.1038/s41590-018-0305-x Medline

59. J. Marcandalli, B. Fiala, S. Ols, M. Perotti, W. de van der Schueren, J. Snijder, E. Hodge, M. Benhaim, R. Ravichandran, L. Carter, W. Sheffler, L. Brunner, M. Lawrenz, P. Dubois, A. Lanzavecchia, F. Sallusto, K. K. Lee, D. Veesler, C. E. Correnti, L. J. Stewart, D. Baker, K. Loré, L. Perez, N. P. King, Induction of Potent Neutralizing Antibody Responses by a Designed Protein Nanoparticle Vaccine for Respiratory Syncytial Virus. *Cell* **176**, 1420–1431.e17 (2019). doi:10.1016/j.cell.2019.01.046 Medline

60. J. Tan, B. K. Sack, D. Oyen, I. Zenklusen, L. Piccoli, S. Barbieri, M. Foglierini, C. S. Fregni, J. Marcandalli, S. Jongo, S. Abdulla, L. Perez, G. Corradin, L. Varani, F. Sallusto, B. K. L. Sim, S. L. Hoffman, S. H. I. Kappe, C. Daubenberger, I. A. Wilson, A. Lanzavecchia, A public antibody lineage that potently inhibits malaria infection through dual binding to the circumsporozoite protein. *Nat. Med.* **24**, 401–407 (2018). doi:10.1038/nm.4513 Medline

61. M. A. Tortorici, N. Czudnochowski, T. N. Starr, R. Marzi, A. C. Walls, F. Zatta, J. E. Bowen, S. Jaconi, J. di Iulio, Z. Wang, A. De Marco, S. K. Zepeda, D. Pinto, Z. Liu, M. Beltramello, I. Bartha, M. P. Housley, F. A. Lempp, L. E. Rosen, E. Dellota Jr., H. Kaiser, M. Montiel-Ruiz, J. Zhou, A. Addetia, B. Guarino, K. Culap, N. Sprugasci, C. Saliba, E. Vetti, I. Giacchetto-Sasselli, C. S. Fregni, R. Abdelnabi, S.-Y. C. Foo, C. Havenar-Daughton, M. A. Schmid, F. Benigni, E. Cameroni, J. Neyts, A. Telenti, G. Snell, H. W. Virgin, S. P. J. Whelan, J. D. Bloom, D. Corti, D. Veesler, M. S. Pizzuto, Structural basis for broad sarbecovirus neutralization by a human monoclonal antibody. bioRxiv 2021.04.07.438818 [Preprint] (2021). https://doi.org/10.1101/2021.04.07.438818.

62. M. A. Tortorici, A. C. Walls, Y. Lang, C. Wang, Z. Li, D. Koerhuis, G. J. Boons, B. J. Bosch, F. A. Rey, R. J. de Groot, D. Veesler, Structural basis for human coronavirus attachment to sialic acid receptors. *Nat. Struct. Mol. Biol.* **26**, 481–489 (2019). doi:10.1038/s41594-019-0233-y Medline

63. Y. J. Park, A. C. Walls, Z. Wang, M. M. Sauer, W. Li, M. A. Tortorici, B. J. Bosch, F. DiMaio, D. Veesler, Structures of MERS-CoV spike glycoprotein in complex with sialoside attachment receptors. *Nat. Struct. Mol. Biol.* **26**, 1151–1157 (2019). doi:10.1038/s41594-019-0334-7 Medline

64. T. N. Starr, N. Czudnochowski, F. Zatta, Y.-J. Park, Z. Liu, A. Addetia, D. Pinto, M. Beltramello, P. Hernandez, A. J. Greaney, R. Marzi, W. G. Glass, I. Zhang, A. S. Dingens, J. E. Bowen, J. A. Wojcechowskyj, A. De Marco, L. E. Rosen, J. Zhou, M. Montiel-Ruiz, H. Kaiser, H. Tucker, M. P. Housley, J. di Iulio, G. Lombardo, M.

Agostini, N. Sprugasci, K. Culap, S. Jaconi, M. Meury, E. Dellota, E. Cameroni, T. I. Croll, J. C. Nix, C. Havenar-Daughton, A. Telenti, F. A. Lempp, M. S. Pizzuto, J. D. Chodera, C. M. Hebner, S. P. J. Whelan, H. W. Virgin, D. Veesler, D. Corti, J. D. Bloom, G. Snell, Antibodies to the SARS-CoV-2 receptor-binding domain that maximize breadth and resistance to viral escape. bioRxiv 2021.04.06.438709 [Preprint] (2021). https://doi.org/10.1101/2021.04.06.438709.

65. A. M. Bolger, M. Lohse, B. Usadel, Trimmomatic: A flexible trimmer for Illumina sequence data. *Bioinformatics* **30**, 2114–2120 (2014). doi:10.1093/bioinformatics/btu170 Medline

66. H. Li, Aligning sequence reads, clone sequences and assembly contigs with BWA-MEM. arXiv:1303.3997 [q-bio.GN] (2013).

67. A. Wilm, P. P. K. Aw, D. Bertrand, G. H. T. Yeo, S. H. Ong, C. H. Wong, C. C. Khor, R. Petric, M. L. Hibberd, N. Nagarajan, LoFreq: A sequence-quality aware, ultra-sensitive variant caller for uncovering cell-population heterogeneity from high-throughput sequencing datasets. *Nucleic Acids Res.* **40**, 11189–11201 (2012). doi:10.1093/nar/gks918 Medline

68. P. Cingolani, A. Platts, L. Wang, M. Coon, T. Nguyen, L. Wang, S. J. Land, X. Lu, D. M. Ruden, A program for annotating and predicting the effects of single nucleotide polymorphisms, SnpEff. *Fly* **6**, 80–92 (2012). doi:10.4161/fly.19695 Medline

69. B. S. Pedersen, A. R. Quinlan, Mosdepth: Quick coverage calculation for genomes and exomes. *Bioinformatics* **34**, 867–868 (2018). doi:10.1093/bioinformatics/btx699 Medline

70. P. Danecek, J. K. Bonfield, J. Liddle, J. Marshall, V. Ohan, M. O. Pollard, A. Whitwham, T. Keane, S. A. McCarthy, R. M. Davies, H. Li, Twelve years of SAMtools and BCFtools. *Gigascience* **10**, giab008 (2021). doi:10.1093/gigascience/giab008 Medline

71. P. Ewels, M. Magnusson, S. Lundin, M. Käller, MultiQC: Summarize analysis results for multiple tools and samples in a single report. *Bioinformatics* **32**, 3047–3048 (2016). doi:10.1093/bioinformatics/btw354 Medline

72. P. Di Tommaso, M. Chatzou, E. W. Floden, P. P. Barja, E. Palumbo, C. Notredame, Nextflow enables reproducible computational workflows. *Nat. Biotechnol.* **35**, 316–319 (2017). doi:10.1038/nbt.3820 Medline

73. B. Grüning, R. Dale, A. Sjödin, B. A. Chapman, J. Rowe, C. H. Tomkins-Tinch, R. Valieris, J. Köster, Bioconda Team, Bioconda: Sustainable and comprehensive software distribution for the life sciences. *Nat. Methods* **15**, 475–476 (2018). doi:10.1038/s41592-018-0046-7 Medline

74. P. Emsley, B. Lohkamp, W. G. Scott, K. Cowtan, Features and development of *Coot*. *Acta Cryst.* **D66**, 486–501 (2010). doi:10.1107/S0907444910007493 Medline

75. T. I. Croll, *ISOLDE*: A physically realistic environment for model building into low-resolution electron-density maps. *Acta Cryst.* **D74**, 519–530 (2018). doi:10.1107/S2059798318002425 Medline

76. G. N. Murshudov, P. Skubák, A. A. Lebedev, N. S. Pannu, R. A. Steiner, R. A. Nicholls, M. D. Winn, F. Long, A. A. Vagin, *REFMAC5* for the refinement of macromolecular crystal structures. *Acta Cryst.* **D67**, 355–367 (2011). doi:10.1107/S0907444911001314 Medline

77. D. Liebschner, P. V. Afonine, M. L. Baker, G. Bunkóczi, V. B. Chen, T. I. Croll, B. Hintze, L. W. Hung, S. Jain, A. J. McCoy, N. W. Moriarty, R. D. Oeffner, B. K. Poon, M. G. Prisant, R. J. Read, J. S. Richardson, D. C. Richardson, M. D. Sammito, O. V. Sobolev, D. H. Stockwell, T. C. Terwilliger, A. G. Urzhumtsev, L. L. Videau, C. J. Williams, P. D. Adams, Macromolecular structure determination using X-rays, neutrons and electrons: Recent developments in *Phenix*. *Acta Cryst.* **D75**, 861–877 (2019). doi:10.1107/S2059798319011471 Medline

78. C. Suloway, J. Pulokas, D. Fellmann, A. Cheng, F. Guerra, J. Quispe, S. Stagg, C. S. Potter, B. Carragher, Automated molecular microscopy: The new Leginon system. *J. Struct. Biol.* **151**, 41–60 (2005). doi:10.1016/j.jsb.2005.03.010 Medline

79. D. Tegunov, P. Cramer, Real-time cryo-electron microscopy data preprocessing with Warp. *Nat. Methods* **16**, 1146–1152 (2019). doi:10.1038/s41592-019-0580-y Medline

80. A. Punjani, J. L. Rubinstein, D. J. Fleet, M. A. Brubaker, cryoSPARC: Algorithms for rapid unsupervised cryo-EM structure determination. *Nat. Methods* **14**, 290–296 (2017). doi:10.1038/nmeth.4169 Medline

81. J. Zivanov, T. Nakane, S. H. W. Scheres, A Bayesian approach to beam-induced motion correction in cryo-EM single-particle analysis. *IUCrJ* **6**, 5–17 (2019). doi:10.1107/S205225251801463X Medline

82. S. Chen, G. McMullan, A. R. Faruqi, G. N. Murshudov, J. M. Short, S. H. Scheres, R. Henderson, High-resolution noise substitution to measure overfitting and validate resolution in 3D structure determination by single particle electron cryomicroscopy. *Ultramicroscopy* **135**, 24–35 (2013). doi:10.1016/j.ultramic.2013.06.004 Medline

83. E. F. Pettersen, T. D. Goddard, C. C. Huang, G. S. Couch, D. M. Greenblatt, E. C. Meng, T. E. Ferrin, UCSF Chimera—A visualization system for exploratory research and analysis. *J. Comput. Chem.* **25**, 1605–1612 (2004). doi:10.1002/jcc.20084 Medline

84. L. J. Reed, H. Muench, A Simple Method of Estimating Fifty Per Cent Endpoints. *Am. J. Epidemiol.* **27**, 493–497 (1938). doi:10.1093/oxfordjournals.aje.a118408

85. R. Abdelnabi, R. Boudewijns, C. S. Foo, L. Seldeslachts, L. Sanchez-Felipe, X. Zhang, L. Delang, P. Maes, S. J. F. Kaptein, B. Weynand, G. V. Velde, J. Neyts, K. Dallmeier, Comparing infectivity and virulence of emerging SARS-CoV-2 variants in Syrian hamsters. *EBioMedicine* **68**, 103403 (2021). doi:10.1016/j.ebiom.2021.103403 Medline

86. M. Foglierini, L. Pappas, A. Lanzavecchia, D. Corti, L. Perez, AncesTree: An interactive immunoglobulin lineage tree visualizer. *PLOS Comput. Biol.* **16**, e1007731 (2020). doi:10.1371/journal.pcbi.1007731 Medline
